# Exploring the substrate scope of ferulic acid decarboxylase (FDC1) from *Saccharomyces cerevisiae*

**DOI:** 10.1038/s41598-018-36977-x

**Published:** 2019-01-24

**Authors:** Emma Zsófia Aletta Nagy, Csaba Levente Nagy, Alina Filip, Katalin Nagy, Emese Gál, Róbert Tőtős, László Poppe, Csaba Paizs, László Csaba Bencze

**Affiliations:** 1 Biocatalysis and Biotransformation Research Center, Faculty of Chemistry and Chemical Engineering, Babeș-Bolyai University of Cluj-Napoca, Arany János nr. 11, Cluj-Napoca, RO-400028 Romania; 2 Department of Organic Chemistry and Technology, Budapest University of Technology and Economics, Műegyetem rkp. 3, H-1111 Budapest, Hungary

## Abstract

Ferulic acid decarboxylase from *Saccharomyces cerevisiae* (*Sc*FDC1) was described to possess a novel, prenylated flavin mononucleotide cofactor (prFMN) providing the first enzymatic 1,3-dipolar cycloaddition mechanism. The high tolerance of the enzyme towards several non-natural substrates, combined with its high quality, atomic resolution structure nominates FDC1 an ideal candidate as flexible biocatalyst for decarboxylation reactions leading to synthetically valuable styrenes. Herein the substrate scope of *Sc*FDC1 is explored on substituted cinnamic acids bearing different functional groups (–OCH_3_, –CF_3_ or –Br) at all positions of the phenyl ring (*o*−, *m*−, *p*−)_,_ as well as on several biaryl and heteroaryl cinnamic acid analogues or derivatives with extended alkyl chain. It was found that *E. coli* whole cells expressing recombinant *Sc*FDC1 could transform a large variety of substrates with high conversion, including several bulky aryl and heteroaryl cinnamic acid analogues, that characterize *Sc*FDC1 as versatile and highly efficient biocatalyst. Computational studies revealed energetically favoured inactive binding positions and limited active site accessibility for bulky and non-linear substrates, such as 2-phenylthiazol-4-yl-, phenothiazine-2-yl- and 5-(4-bromophenyl)furan-2-yl) acrylic acids. In accordance with the computational predictions, site-directed mutagenesis of residue I330 provided variants with catalytic activity towards phenothiazine-2-yl acrylic acid and provides a basis for altering the substrate specificity of ScFDC1 by structure based rational design.

## Introduction

Styrenes are valuable building blocks for the synthesis of fine chemicals, polymers and pharmaceutically active compounds. Accordingly biotechnologies for their synthesis continuously emerged, styrene production from glucose through engineered *Escherichia coli* cells, or *Saccharomyces cerevisiae* cells with improved phenotypes have been successfully developed^1,2^. These methodologies rely on the activity of ferulic acid decarboxylase (FDC1) on cinnamic acid, a metabolic intermediate of the shikimate pathway. However, the synthesis of differently functionalized styrenes through metabolic pathways still remains challenging, due to the limited substrate specificity of metabolic enzymes and the complexity of constructing such artificial metabolic pathways^1,2^. Through more convenient biocatalytic procedures, by the use of non-oxidative decarboxylases as whole cells, cell free extracts or isolated biocatalysts, bioproduction of styrene derivatives can also be approached^3–6^. The accessibility of commonly available cinnamic acid derivatives as starting materials, the mild and environmentally friendly reaction conditions render the decarboxylation approach appealing.

Regardless to the mechanism of action and the nature of the employed cofactor, currently, four distinct types of non-oxidative decarboxylases acting on aromatic acids have been described. Phenolic acid decarboxylases form *Enterobacter sp*., *Bacillus pumilus* and *Lactobacillus sp*. do not require cofactor and have strict substrate specificity to cinnamic acid derivatives possessing the 4-OH functional group^7–9^. Members of amidohydrolase superfamily (AHS), a diverse group of metallo-dependent enzymes, with broad range of catalytic diversity, hydrolyzing C-O, P-O, P-S, C-N, C-S, and C-Cl bonds^10–14^, were also shown to act on C-C bond cleavage of substituted benzoic acids. 5-Carboxyvanillate decarboxylase (LigW) was reported to catalyze the nonoxidative C−C bond cleavage of 5-carboxyvanillate (5-CV)^15^, while benzoic acid decarboxylases, showing high protein sequence homology with amidohydrolases^16^, decarboxylate diverse hydroxybenzoic acids^17^.

Phenylacrylic acid decarboxylases (PADs) are flavoproteins with a non-covalently bound flavin mononucleotide. Their most known representative is PAD1 from *E. coli*, also known as UbiX, which catalyse the decarboxylation of 3-octaprenyl-4-hydroxybenzoate in the ubiquinone biosynthesis^18^. In *E. coli* besides UbiX, another decarboxylase, UbiD, is also known to be involved in ubiquinone biosynthesis^18^. The homologues of UbiX and UbiD in *Saccharomyces cerevisiae* are PAD1 and FDC1, respectively, which were found to be employed in the decarboxylation of aromatic carboxylic acids, like ferulic acid, *p*-coumaric acid or cinnamic acid, both *pad1* and *fdc1* genes being required for the decarboxylation activity^19^.

Recently, FDC1 from *Aspergillus niger* and *S. cerevisiae* was shown to possess a novel prenylated flavin mononucleotide cofactor (prFMN), while PAD1 was found to play role in the formation of the catalytically active, modified FMN-cofactor of FDC1^6^. The mechanism of the FDC1 catalysed decarboxylation was the first example for an enzymatic 1,3-dipolar cycloaddition^6,20^. Importantly, from biocatalytic point of view, several differently substituted cinnamic acid derivatives were shown to be good or moderate substrates of *Sc*FDC1^21^. The presumably broad substrate tolerance nominates *Sc*FDC1 as potential biocatalyst for decarboxylations leading to synthetically valuable styrenes. Moreover the availability of high quality, atomic resolution structures with the bound prFMN and various inhibitors^6^ enables structure-based rational modification of *Sc*FDC1. The tedious isolation process of the enzyme, requiring the bacterial co-expression with truncated *Sc*PAD1 necessary for the production of the active prenylated FMN cofactor of FDC1, can be avoided by using as biocatalyst *E. coli* whole cells harbouring only the *fdc1* gene, since UbiX of the host *E. coli* substitutes *Sc*PAD1 in its role to provide the prFMN^22^.

Herein, in our aim to develop biocatalytic routes to various styrene derivatives we explored the substrate scope of *Sc*FDC1, using whole-cells of *E. coli* harbouring the *fdc1* gene of *S. cerevisiae* as biocatalyst. Since earlier studies focused mostly on cinnamic acid derivatives with functional groups at the 4-position of the phenyl group^21^, we investigated whether *Sc*FDC1 accepts differently (*o*-,*m*-,*p*-) substituted phenyl-, bulky heteroaryl- or biaryl-analogues of cinnamic acid.

## Results and Discussion

### Generation of substrate library and decarboxylation activity assay

**Figure 1:**
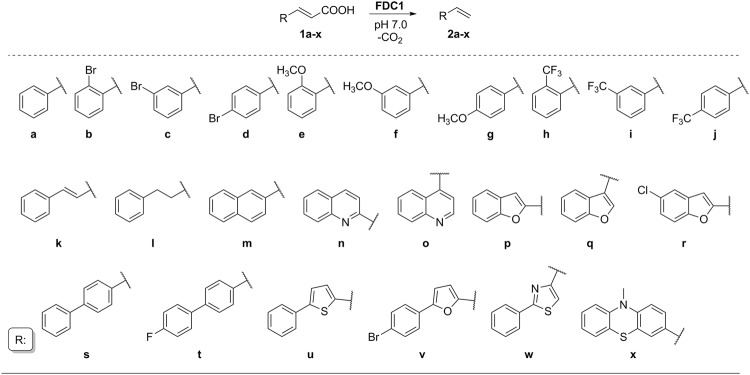
The FDC1 catalyzed decarboxylation reaction of cinnamic acid analogues 1**a**–**x**.

The tested substrate library was obtained through Knoevenagel–Doebner reaction (see further details in ESI) and included (*i*) cinnamic acid analogues with functional groups (–Br, –OCH_3_ and –CF_3_) in different positions (*o−, m−, p−*) of the aromatic ring (**1a**–**j**), or (*ii*) substrate analogues with extended alkenyl or alkyl chains (styrylacrylate **1k** and 5-phenylpent-2-enoic acid **1l**) as well as (*iii*) several biaryl and heteroaryl analogues of cinnamic acid (**1m**–**x**) (Fig. 1). While *p*-bromo, and *m*-, *p*-methoxy- cinnamic acids (**1d,f,g**) are known substrates of *Sc*FDC1^21^, to our best knowledge no reports exist on the activity of *Sc*FDC1 with other bulky biaryl- or heteroaryl analogues of cinnamic acids or with the extended alk(en)yl chain-containing cinnamic acid analogues (**1a**–**c**,**e**,**h**-**x**).

Whole cells of *E. coli* BL21 (DE3) pLysS expressing *Sc*FDC1 from plasmid *pTfdc1Sc*^1^ after proper induction were used as biocatalyst to perform the biotransformations of **1a**–**x** to **2a**–**x** (Fig. 1). The conversions in *Sc*FDC1-catalysed biotransformations were determined by monitoring substrate depletion using reversed-phase HPLC, with *o*-anisol as internal standard calibrated to authentic cinnamic acid derivatives **1a**–**x** (Figs S1–S24). Formation of the corresponding styrene derivatives was confirmed by GC-MS (Figs S25–S60).

### Initial screening for the decarboxylation of the substrate library

Initial screening of the substrate panel for decarboxylation activity of *Sc*FDC1 was performed with whole cell suspensions reaching optical density (OD_600_) of 1 at 30 °C, pH 7.0 and 1 mM substrate concentration. *Sc*FDC1 could decarboxylate a broad range of variously substituted cinnamic acid analogues (**1a**–**j**) as biaryl (naphthyl- or biphenyl-) **1m,s,t** or heteroaryl (quinolinyl, benzofuranyl and 5-phenylthiophenyl) **1n–r,u** acrylates (Table S1). Styrylacrylate **1k**, with extended conjugation and chain length was also transformed with high conversion by *Sc*FDC1, while in case of its non-conjugated analogue **1l** was inert. However, *Sc*FDC1 could not decarboxylate bulky phenothiazine-2-ylacrylate **1x**, 3-(5-(4-bromophenyl)furan-2-yl)acrylic acid **1v**, or (2-phenylthiazol-4-yl)acrylate **1w**. In accordance with the conversions from **1a**–**k** and **1m–u** indicated by HPLC, styrene derivatives **2a**–**k** and **2m–u** were detected as products by GC-MS (Figs S27–S68). Neither by HPLC nor by GC-MS could be observed products corresponding to structures **2l,v,w,x**. Control experiments performed in the absence of biocatalyst or using *E. coli* expression host cells lacking the *pTfdc1Sc* plasmid supported the requirement of FDC1 for product formation and excluded spontaneous background reactions (Figs S25,S26).

### Optimization of the whole-cell biotransformations

**Figure 2:**
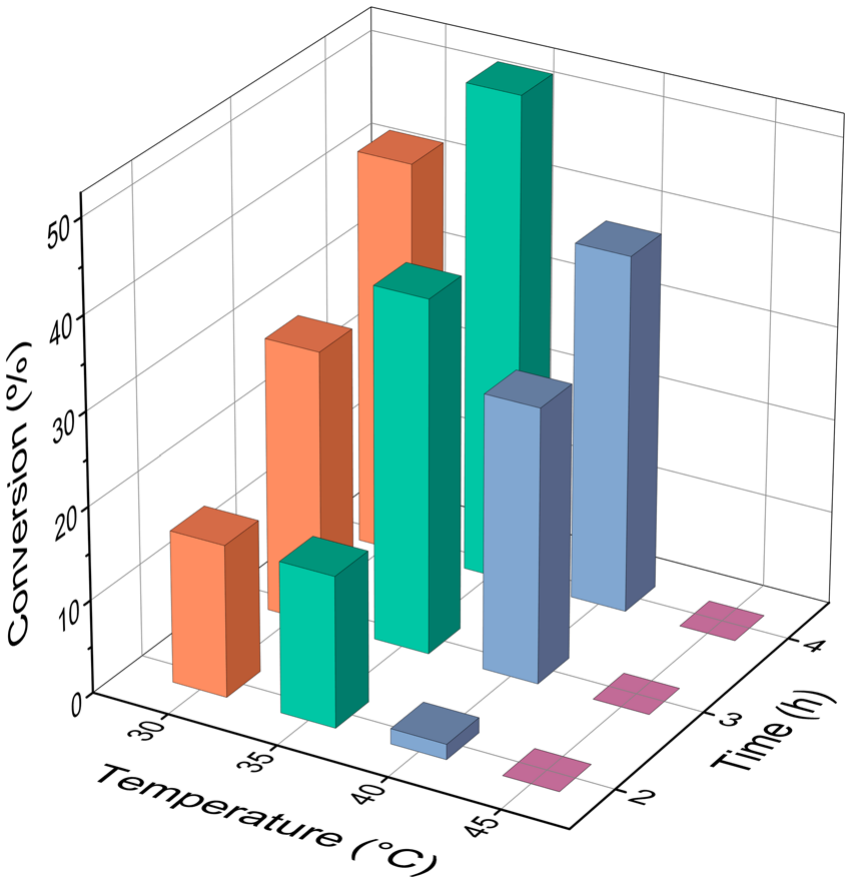
The effect of the temperature upon the conversion of 1**i** in the *Sc*FDC1-mediated decarboxylation.

Next, the reaction conditions of whole-cell biotransformations were optimized focusing on the effect of pH, temperature and biocatalysts/substrate ratio upon conversion, using (3-(3-(trifluoromethyl)phenyl)acrylic acid (**1i**) as model substrate. The study for biotransformations in buffers of various pH values ranging from 6.0–8.0 revealed the highest degree of conversion at pH values of 6.5 and 7.0 (Fig. S70), in accordance with the reported pH optimum for the purified FDC1 enzyme^21^. The optimal temperature was found to be 35 °C, at lower temperatures conversion values significantly decreased, while at 45 °C no product formation was observed (Fig. 2).

The effect of biocatalyst/substrate ratio on conversion values was tested by using different amounts of whole-cell biocatalysts (OD_600_ of 1,2 or 3) at a fixed, 2 mM substrate concentration of **1i**. Expectedly, shorter reaction times were achieved with increasing amount of whole-cell *Sc*FDC1 biocatalyst (the reaction after 4 h at OD_600_ = 1, 2 and 3 resulted in conversion of 42%, 76% and ~100%, respectively). In further experiments, comparison of the *Sc*FDC1 activity with different substrates was performed at moderate cell densities (OD_600_ ≤ 2) to provide sufficiently long reaction time for precise monitoring of the time-course profile.

**Figure 3:**
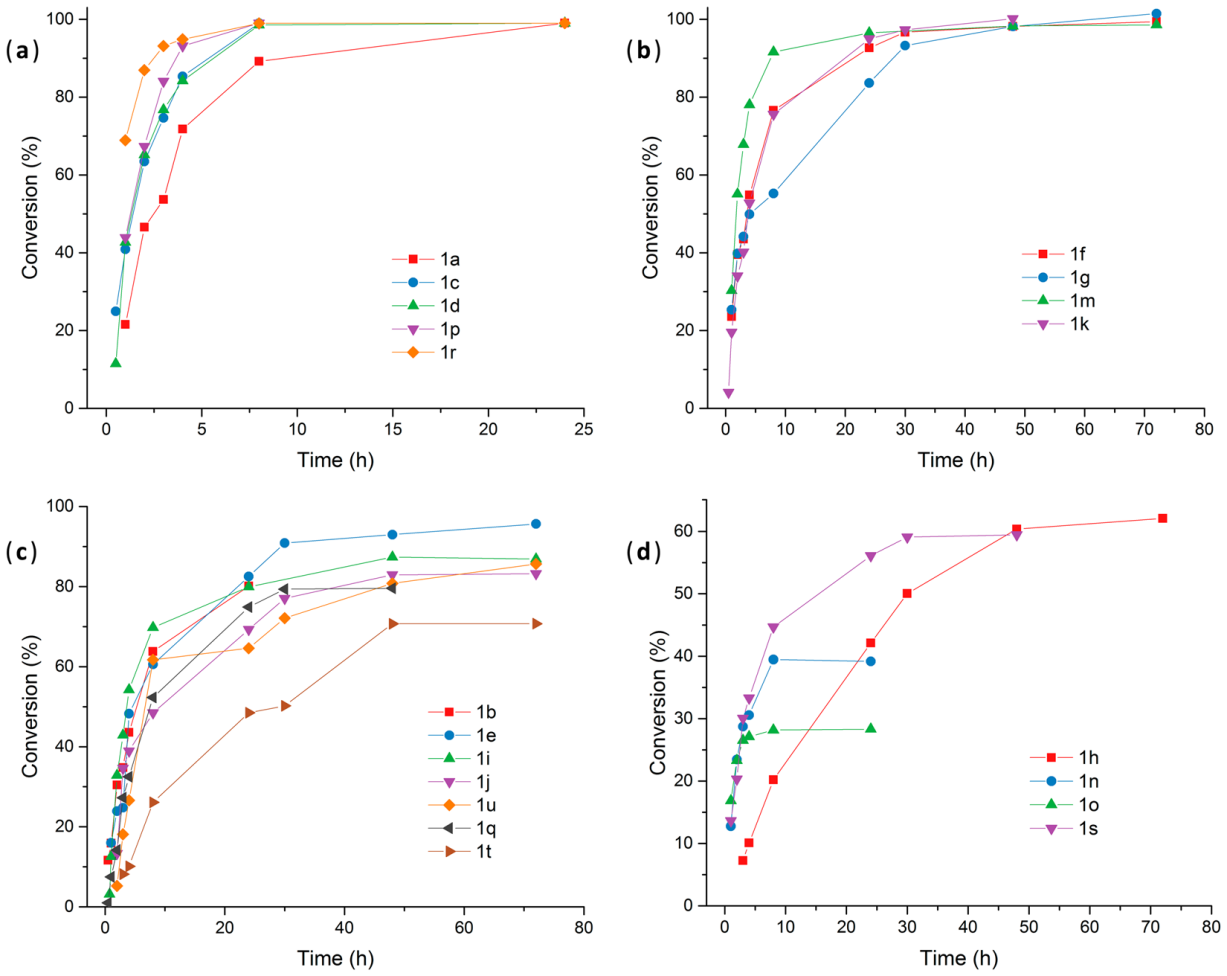
The conversion progression curves of the whole-cell FDC1 decarboxylation reactions of (**a**) substrates **1a**,**c**,**d**,**r**,**p** providing complete conversion in relatively short reaction time (under 24 h), (**b**) substrates **1f**,**g**,**m**,**k** providing complete conversions in longer reaction time (over 24–72 h), (**c**) substrates **1b**,**e**,**i**,**j**,**q**,**t**,**u**, with incomplete, but high conversions and (**d**) substrates **1h**,**n**,**o**,**s** providing moderate or low conversion within 72 h reaction time.

Finally, the FDC1-catalyzed reactions of the entire substrate panel (**2a–x**) were performed under the optimal reaction conditions (100 mM sodium phosphate buffer pH 7.0, cells OD_600_ of 1, 35 °C), monitoring the conversions over longer time period (Table 1, Fig. 3a–d).

**Table 1:** *Sc*FDC1-containing whole-cell biotransformations of **1a–x**: maximal conversions obtained under optimized conditions (100 mM sodium phosphate buffer pH 7.0, cells OD_600_ of ~1, 35 °C).

Substrate		t (h)	c^*^ (%)	E_b_^**^ (kcal/mol)	LUMO (eV)	Planarity^***^
cinnamic acid	**1a**	24	>99	−6.6	1.681	1
(*E*)-3-(2-bromophenyl)acrylic acid	**1b**	48	83^[a]^	−6.4	1.502	0
(*E*)-3-(3-bromophenyl)acrylic acid	**1c**	8	>99	−5.9	1.305	1
(*E*)-3-(4-bromophenyl)acrylic acid	**1d**	24	>99	−5.2	1.313	1
(*E*)-3-(2-methoxyphenyl)acrylic acid	**1e**	48	92^[a]^	−5.9	1.579	1
(*E*)-3-(3-methoxyphenyl)acrylic acid	**1f**	48	>99	−5.5	1.638	1
(*E*)-3-(4-methoxyphenyl)acrylic acid	**1g**	72	>99	−5.7	1.376	1
(*E*)-3-(2-(trifluoromethyl)phenyl)acrylic acid	**1h**	72	62^[a]^	−6.2	1.212	0
(*E*)-3-(3-(trifluoromethyl)phenyl)acrylic acid	**1i**	48	87^[a]^	−5.0	1.170	1
(*E*)-3-(4-(trifluoromethyl)phenyl)acrylic acid	**1j**	48	82^[a]^	−4.3	0.931	1
(2*E*,4*E*)-5-phenylpenta-2,4-dienoic acid	**1k**	48	>99	−4.4	0.957	1
(*E*)-5-phenylpent-2-enoic acid	**1l**	72	<1	−5.7^[c]^	1.450	0
(*E*)-3-(naphthalen-2-yl)acrylic acid	**1m**	48	>99	−4.7	0.924	1
(*E*)-3-(quinolin-2-yl)acrylic acid	**1n**	8	39^[b]^	−2.5	0.701	0
(*E*)-3-(quinolin-4-yl)acrylic acid	**1o**	8	28^[b]^	−6.0	0.638	0
(*E*)-3-(benzofuran-2-yl)acrylic acid	**1p**	8	>99	−4.9	1.222	1
(*E*)-3-(benzofuran-3-yl)acrylic acid	**1q**	30	79^[a]^	−5.4	1.421	1
(*E*)-3-(5-chlorobenzofuran-2-yl)acrylic acid	**1r**	8	>99	−3.2	0.909	1
(*E*)-3-([1,1′-biphenyl]-4-yl)acrylic acid	**1s**	30	59^[a]^	−3.8	0.822	0
(*E*)-3-(4′-fluoro-[1,1′-biphenyl]-4-yl)acrylic acid	**1t**	48	70^[a]^	−3.7	0.775	0
(*E*)-3-(5-phenylthiophen-2-yl)acrylic acid	**1u**	72	85^[a]^	−3.2	0.654	0
(*E*)-3-(5-(4-bromophenyl)furan-2-yl)acrylic acid	**1v**	72	<1	−3.6^[d]^	0.556	1
(*E*)-3-(2-phenylthiazol-4-yl)acrylic acid	**1w**	72	<1	−2.8^[c]^	0.429	1
(*E*)-3-(10-methyl-10*H*-phenothiazin-2-yl)acrylic acid	**1x**	72	<1	−^[e]^	1.059	0

^[a]^Complete conversion reached after additional 24 h reaction time with fresh cell batch; ^[b]^no conversion increase after additional 24 h reaction time with fresh cell batch; ^[c]^unfavourable binding pose of the acrylic double bond related to the prFMN cofactor; ^[d]^unfavourable, reverse binding pose – the substrate’s carboxyl group located distant from R175; ^[e]^positioning within the catalytic site was not found, substrate binding occurs in a surface pocket close to the catalytic site; *Determined through monitoring the substrate depletion by HPLC; **E_b_ – binding energy; ***1 indicates planar structure of substrate and 0 otherwise.

While substrates **1a,c,d,f,g,k,m,p,r** were fully converted within short (<24 h, Fig. 3a, Table 1) or moderate (<72 h, Fig. 3b, Table 1) reaction time, in case of substrates **1b,e,i,j,q,t,u** the reactions ceased at high, but not full conversions after 72 h (Fig. 3c, Table 1). Moderate or low conversion values were obtained with substrates **1h,n,o,s**, (Fig. 3d, Table 1) while using substrates **1l,v,w,x** neither substrate depletion or product formation could be detected (Table 1).

To study the effect of cell viability loss over long reaction times, to the reactions not reaching full conversion within 72 h (Fig. 3c,d, Table 1) were added additional batch of fresh cells (OD_600_ = 1) when the reaction ceased. In this way the reactions, initially providing moderate to good conversions (substrates **1b,e,h,i,j,q,s,t,u)**, proceeded with complete transformation within additional 24 h after supplementing the reaction with fresh cells. This behaviour indicated cell viability issues over prolonged reaction times. However, in case of substrates **1n,1o** supplementation with further cells led to only moderate increase of conversion indicating product inhibition or product toxicity upon cells.

### Computational studies

**Figure 4:**
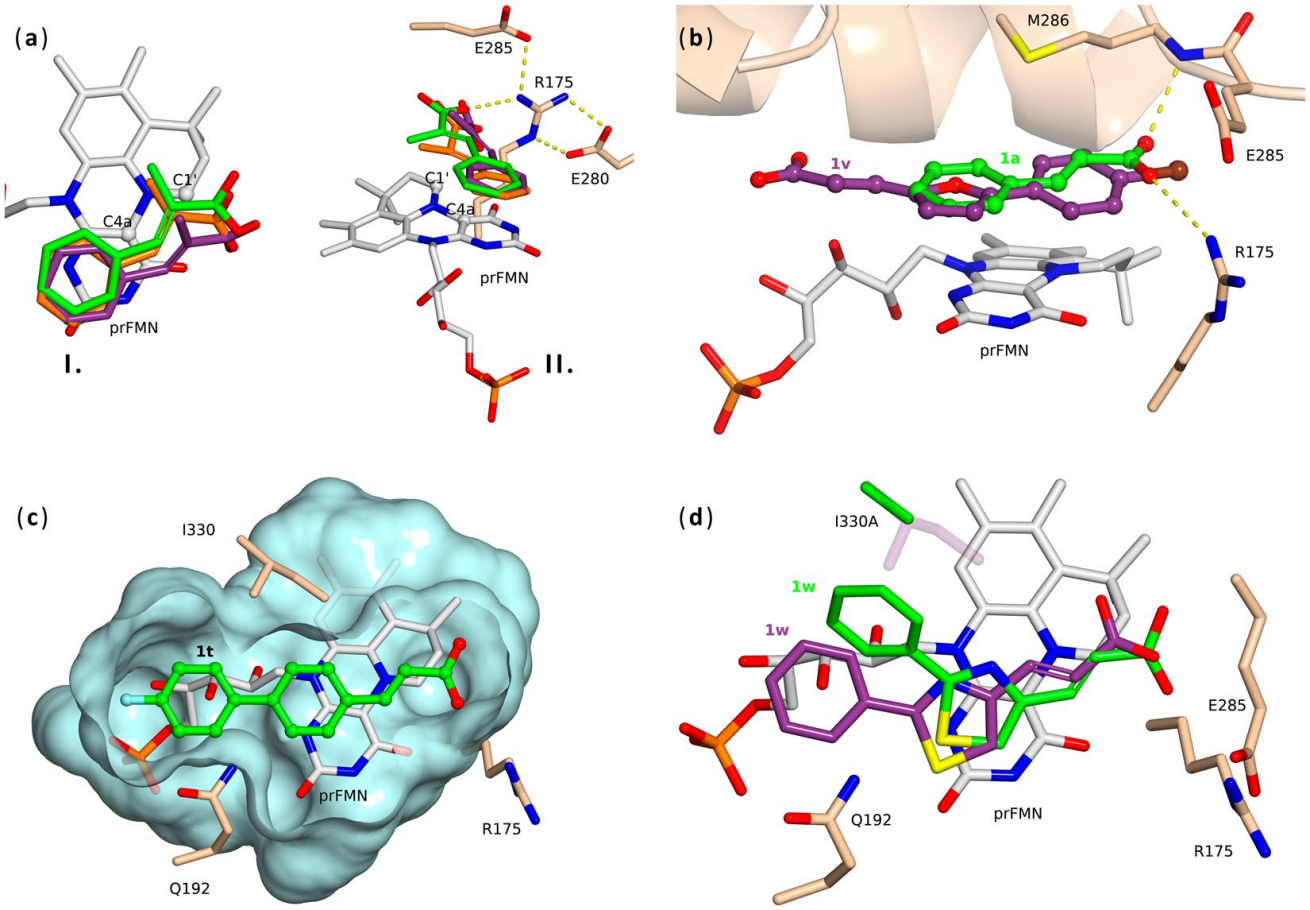
(**a**) Comparison of ligand (α-methyl *trans*-cinnamate) conformations taken from the 4ZA7 crystal structure (violet) with the lowest energy docking pose (green), and with the reported transition state geometry^24^ (orange) of 1,3-dipolar cycloaddition. **I**. – top view - the alignment of the double bond with respect to the prFMN cofactor in the docking conformation is in good agreement with the transition state geometry obtained by QM/MM study; **II**. – front view – substrate orientation related to prFMN and key residues R175, E280 and E285. (**b**) The energetically favoured, inactive orientation of **1v** (purple) (similar positioning obtained also in case of **1w**) - with E285 and prFMN distant from carboxyl group and the acrylic double bond of the substrate – compared to the active orientation of **1a** (green). (**c**) Surface representation (blue) of the FDC1 binding pocket with substrate **1t**, that displays the role of residues Q192 and I330 in narrowing the binding site in the proximity of the substrate’s aryl group; (**d**) binding of **1w** in the active site of mutant I330A mutant (green), with the double bond in favourable position related to the C1′ and C4a of prFMN, compared to its inactive positioning in the *wt-*FDC1 (deep purple); steric hindrance between residue I330 (purple, paled) and the aryl group of the properly positioned **1w** (green) occurs and is removed through mutation I330A.

As both experimental^6,20,21^ and theoretical^23,24^ results confirmed, the FDC1-catalyzed decarboxylation follows a so far unprecendented enzymatic 1,3-dipolar cycloaddition mechanism involving the formation of a covalent substrate-prFMN adduct. While the inductive effect of substituents of the dipolarophile substrate are known to increase the 1,3-cycloaddition reaction rate of nitrogen ylide dipoles, such as prFMN, in case of FDC1 catalyzed reaction it was shown that the presence of an extended π-system associated with the aromatic ring of the substrate also has an important role in stabilization of the transition state^21^, supporting the assumption that π-stacking interaction exists between the planar cofactor and the aromatic moiety of substrate. Therefore, proper binding interaction implies the location of the α,β-double bond of the substrate in proximity to the C1′ and C4a atoms of the cofactor (Fig. 4a). The substrate orientation is further facilitated by R175, interacting with the carboxyl group of the substrate, while the other key residues are E285, acting as acid-base in the reaction mechanism, and E280, which presumably tunes the pKa of R175 and in turn E285 (Fig. 4a)^25^. Accordingly, the reaction rates are influenced by multiple substrate-related factors, such as inductive effects of substituents, presence of extended conjugation, substrate orientation related to the prFMN and within the catalytic site, influenced by both size and planarity of substrate.

Therefore, to rationalize the different rates of conversion for substrates **1a**–**x**, the possibility of planar arrangement of their molecular skeleton, their LUMO energies and orientation in the active site of FDC1 were computationally explored.

Crystal structures of FDC1 (PDB code: 4ZA7, 4ZA8) disclose that the ring system of prFMN adopts a planar conformation to facilitate substrate binding^6^. Consequently, the planarity of the substrates was explored computationally using symmetry/geometry constraints (Table 1). Imaginary frequencies confirmed that planar conformations of structures **1b,h,l,n,o,s,t,u,x** correspond to transition states, while their lowest energy conformations deviate from planarity (e.g. at the carboxylic group in case of *ortho-*substituted ligands **1b,h** or between the two aromatic rings in case of biphenyl acrylic acids **1s,t**). These results were in agreement with the experiments indicating that maximal conversion could not be achieved with non-planar substrates and in some cases even no conversions could be observed (Table 1). On the other hand, substrates **1a**,**c**–**g,i,j,k**,**m**,**p**,**q**,**r** with planar ground state conformation showed high decarboxylation rates, suggesting a good correlation between substrate planarity and reaction rates (Table 1).

The LUMO energies of the dipolarophile substrates provided further insights into the FDC1 reaction. In case of substrates bearing similar heteroaryl moieties, such as benzofuranyl acrylic acids **1p,q,r**, quinolin derivatives **1n,o** or biphenyacrylates **1s,t**, the conversion increased with decreased LUMO energies (Table 1). In case of mono-substituted cinnamic acid analogues **1a–j** no significant correlation between LUMO energy levels and conversion rates could be observed, which correlated with the reported negative slope of the Hammet plot obtained for the FDC1-catalyzed reactions of *para*-substituted cinnamic acids^21^, supporting the significant influence of electron delocalization (besides the inductive effects) in transition state stabilization.

Docking results (Table 1) revealed the best binding affinities for substrates **1a–j** containing one aromatic ring. Importantly, in case of bulky substrates inactive binding poses were also identified. In case of bulky ligands **1v,w,x** substrate binding occurs in a surface pocket close to the catalytic site when using large grid box of 16 or 20 Å (illustrated for **1x** in Fig. S72). In case of compounds **1v,w** higher energy active site binding poses were also obtained within the 14 Å grid box (Table 1), with distant arrangement of the catalytically important E285, R175 residues and the prFMN cofactor from the substrate’s carboxyl group and the acrylic double bond, correspondingly (Fig. 4b). While in case of 5-(4-bromophenyl)furan-2-yl)acrylic acid **1v** presumably the length of the substrate exceeds the limits of the catalytic site for active binding position, in case of (2-phenylthiazol-4-yl)acrylic acid **1w** positioning with proper orientation of the carboxyl group towards residue E285 and R175 was also obtained, however the arrangement of the α,β-double bond with respect to the C1′ and C4a atoms of the prFMN cofactor was unfavourable (Fig. S73). In case of **1l**, the lack of the extended π-system and the unfavourable arrangement of the acrylic double bond due to the non-planar molecular skeleton contributed to the negligible reactivity (Fig. S73).

To extend the computational investigations of substrates showing no conversion, the ground state of the substrate-cofactor covalent intermediate resulting after the 1,3-cycloaddition of various substrates to prFMN was computed in gas phase. The obtained gas phase geometries were overlaid on the prFMN crystal structure and clashes between the substrate and active site residues were evaluated. Comparison and agreement of this calculation method for *trans*-cinnamic acid **1a** to the reported gas-phase^6^ and QM/MM^23^ results validated this mode of modelling. The result in case of **1v** adduct indicated, that although the ground state conformation of the intermediate is favourable and is aligned along the linear catalytic site, the short distance between the bromine and enzyme backbone prevents the substrate from fitting into the active site (Fig. S74). Fitting the covalent intermediate forming from **1w** and prFMN into the enzyme revealed severe steric clashes between the substrate sulphur atom and residue I330 (Fig. S75). Accordingly, it seems that I330 and Q192 narrows the active site in proximity to the substituent placed on the phenyl group of substrate, forming a gate, which can be passed by bulky, but only linearly oriented substrates, such as 3-([1,1′-biphenyl]-4-yl)acrylic acid **1s** and 3-(4′-fluoro-[1,1′-biphenyl]-4-yl)acrylic acid **1t** (Fig. 4c). The non-linear ligands such as (2-phenylthiazol-4-yl)acrylic acid **1w** show steric clash either with the gate-forming residues (Fig. S75) or, in case of 3-(5-(4-bromophenyl)furan-2-yl)acrylic acid **1v**, with a backbone carbonyl (Fig. S74). Interestingly, in case of the less bulky bicyclic aryl substrates **1m**–**r**, the gate-forming residues (I330 and Q192) do not hinder the active orientation of the ligand (Fig. S76).

To validate the interactions observed in modelling, residues Q192 and I330 were replaced by smaller residues using site-directed mutagenesis. Since Q192 is also involved in cofactor binding through hydrogen bonding with the ribitol tail of prFMN (Fig. S77), mutation Q192N was envisaged. While catalytic activity of the Q192N mutant towards **1a** was maintained (92% conversion after 24 h reaction time), this mutant remained inactive with substrates **1v,w** (Table 2). On the other hand, the I330V and I330A mutants displayed activity towards **1w**, resulting in moderate conversions (5 and 15% with mutants I330V and I330A, respectively) after 24 h reaction time (Table 2). Besides the improved binding energy of **1w** to the active site of FDC1 I330A in comparison with the one obtained within the active site of *wt*-enzyme (E_b (F133A FDC1)_ = –3.5 kcal/mol and E_b (*wt*-FDC1)_ = –2.8 kcal/mol), the arrangement of the acrylic double bond compared to prFMN also changed to the favourable position (Fig. 4d). Similarly to the *wt*-FDC1, none of the mutants exhibited activity towards 3-(5-(4-bromophenyl)furan-2-yl)acrylic acid **1v** and 3-(10-methyl-10*H*-phenothiazin-2-yl)acrylic acid **1x** (Table 2), providing further support for docking results indicating that bulky **1v,x** exceed the volume of the active site (Figs S72,S74).

**Table 2:** Conversions of decarboxylation reactions from **1a,v,w** catalysed by *wild-type* (*wt*)-*Sc*FDC1 and *Sc*FDC1 mutants after 24 h.

Substrate		*wt-Sc*FDC1	*Sc*FDC1 mutant		
			I330A	I330V	Q192N
		Conversion (%)			
cinnamic acid	**1a**	>99	>99	>99	92
(*E*)-3-(5-(4-bromophenyl)furan-2-yl)acrylic acid	**1v**	<1	<1	<1	<1
(*E*)-3-(2-phenylthiazol-4-yl)acrylic acid	**1w**	<1	15	5	0
(*E*)-3-(10-methyl-10*H*-phenothiazin-2-yl)acrylic acid	**1x**	<1	<1	<1	<1

The results demonstrate that *Sc*FDC1 possess broad substrate tolerance. Substrate planarity is beneficial for the decarboxylation reaction and is imposed by the flat active site, but also by the formation of the 1,3-cycloaddition adduct with the prFMN cofactor. The revealed limits of substrate tolerance of FDC1 are dictated by the length and flatness of the active site, but also by the passage formed by residues Q192 and I330, which narrows the active site and provides access to its full length only for bulky substrates with planar and linear structure.

## Materials and Methods

### Materials

The commercial chemicals and solvents were products of Sigma Aldrich or Alfa-Aesar. 5-(4-Bromophenyl)furan-2-carbaldehyde was synthesized using the procedure described by us^26^. 2-Phenylthiazole-4-carbaldehyde was synthesized as described by Silberg^27^. 5-Phenylthiophene-2-carbaldehyde, and 4′-fluoro-(1,1′-biphenyl)-4-carbaldehyde were synthesized as described by Bussolari^28^. The plasmid pTfdc1Sc was a kind gift from Prof. David R. Nielsen (Addgene plasmid # 78287)^1^. For site-directed mutagenesis the *fdc1* gene, subcloned into pCDF-Duet1 expression vector through SalΙ and HindIII restriction sites (plasmid pCFDfdc1), was used as template DNA.

### Instrumentation and analytical methods

^1^H- and ^13^C-NMR spectra were obtained using Bruker (Billerica, MA, USA) Avance spectrometers operating at 400 MHz and 101 MHz/600 MHz and 151 MHz, respectively. All spectra were recorded at 25 °C in MeOD-*d*_4_, CDCl_3_ or DMSO-*d*_6_. ^1^H and ^13^C NMR spectra were referenced internally to the solvent signal.

The production of styrenes was confirmed through gas chromatography-mass spectrometry (GC-MS) analysis. The samples were prepared by extracting the biotransformations (see section 3.4.) with *n*-hexane or *tert*-butyl methyl ether and drying the extract over anhydrous sodium sulphate. The GC-MS analyses were performed using a Shimadzu QP 2010 PLUS Mass Spectrometer coupled with Gas Chromatograph (Shimadzu). The mass spectrometry was performed in the electron impact mode (MS/EI) at 70 eV. Peak identification was carried out by analogy of mass spectra with those of the mass library (NIST 2.0 and Wiley). The system was configured as detailed in Table S2.

The HPLC determination of conversion was performed on Agilent 1200 and/or 1260 series high performance liquid chromatography (HPLC) using Gemini NX-C18 150 × 4.5 mm or Zorbax SB-C8 50 × 2.1 mm columns, flow rate: 1 mL/min. The quantification of the conversion values was based on the determination of the consumption of acrylic acid substrates **1a-x** using anisole as internal standard. The details of the HPLC methods used to determine the conversions are described in Table S3.

### Synthesis of (*E*)-arylacrylic acids

The synthesis of acrylic derivatives **1a–j,l–z** was performed by using the corresponding aldehydes **3a–j,l–z** as starting material with the Knoevenagel-Doebner reaction (Fig. S69). Synthesis of styrylacrylate **1k** was performed as earlier reported^29^.

#### Synthesis of compounds 1a–j,l–x

The corresponding aldehyde **3a–j,l–x** (3 mmol), malonic acid (6 mmol, 2 equiv., 0.62 g) and piperidine (2 mol%, 0.06 mmol, 0.06 µl) were dissolved in DMSO (20 mL) and the mixture was heated at 100 °C for 8 h. The solution was diluted with 5% aqueous HCl solution (40 mL), the solid precipitate was filtered, washed with water (20 mL), aqueous Na_2_CO_3_ (10%, 20 mL), water (20 mL), and finally with acetone (20 mL) or diethyl-ether (20 mL), then dried under vacuum.

#### Synthesis of compounds 1g,m

The corresponding aldehyde **3g,m** (3 mmol) and malonic acid (6 mmol, 2 equiv.) were dissolved in pyridine (20 mL) and the mixture was heated under reflux for 6 h, and further stirred at 80 °C overnight. After cooling, the reaction mixture was concentrated to dryness and the remained solid was washed with 5% aqueous HCl solution (20 mL), diethyl-ether (20 mL) and finally dried to afford pure acrylic acids **1g,m** (yields: 82% for **1g** and 78% for **1m**).

The synthesized known compounds **1a–x** were characterized by ^1^H and ^13^C NMR measurements (for NMR data see ESI, Chapter 3), while further HPLC measurements (see ESI, Chapter 2) confirmed their high purity.

### Biotransformation with whole-cell FDC1

#### General procedure

Seed cultures of *E. coli* BL21(DE3) harbouring the corresponding recombinant plasmids, were prepared in 100 mL LB broth and grown overnight. Shake flasks (2 L) containing 500 mL of LB were inoculated with 5 mL of seed culture. Cultures were grown at 37 °C until an OD_600_ of ~0.6 was reached, at which point the cultures were induced by IPTG addition at a final concentration of 0.2 mM. Cultures were incubated for an additional 4.5 h (resulting in an OD_600_ of ~3) before cells were collected and centrifuged at 6000 rpm for 15 min. The pellet was washed with 100 mM sodium phosphate buffer, pH 7.0, followed by resuspension to a final OD_600_ of ~1, aliquoting, centrifugation and storage at –20 °C. Expression of *Sc*FDC1 protein was confirmed by the SDS-PAGE analysis (see Fig. S78).

*Sc*FDC1 activity assays were performed in 1.5 mL glass vials sealed with PTFE septum, with a reaction volume of 1 mL using buffers with different pH (see section “*The effect of pH on biotransformation*”). Stock solutions of substrates were prepared in DMSO. Assay contained substrates at various final concentrations between 0.5–2 mM. The reactions were incubated at different temperatures (see section “*The effect of temperature on biotransformation*”). The samples were taken after 24 h.

Samples from biotransformations were diluted with acetonitrile and analysed by HPLC and GC-MS as described in ESI, Chapter 2.

#### The effect of pH on biotransformations

The influence of pH (6.5, 7.0, 7.5, 8.0) on the FDC1-catalyzed decarboxylation reaction was tested with 3-(3-(trifluoromethyl)phenyl)acrylic acid (**1i**) using 100 mM sodium phosphate buffer. The enzymatic reactions were performed in 1.5 mL glass vials sealed with PTFE septum, with a reaction volume of 1 mL using 100 mM sodium phosphate buffer at pH 6.0, 6.5, 7, 7.5, 8.0, 2 mM substrate concentration and *Sc*FDC1-containing whole cells with OD_600_ of ~1. The reactions were incubated at 30 °C. The samples were taken after 15 h and analysed by HPLC to determine the conversions.

#### The effect of temperature on biotransformations

The influence of temperature (30, 35, 40, 45 °C) on the *Sc*FDC1-catalyzed decarboxylation reaction was tested with 3-(3-(trifluoromethyl)phenyl)acrylic acid (**1i**). The enzymatic reactions were performed in 1.5 mL glass vials sealed with PTFE septum, with a reaction volume of 1 mL using 100 mM sodium phosphate buffer at pH 7.0., 2 mM substrate concentration and *Sc*FDC1-containing whole cells with OD_600_ of ~1. The reactions were incubated at different temperatures (30, 35, 40, 45 °C). The samples were taken after 15 h, and analysed by HPLC to determine the conversions.

#### Effect of cells quantity on biotransformation

The screening contained 3-(3-(trifluoromethyl)phenyl) acrylic acid (**1i**, 2 mM) and *Sc*FDC1-containing whole cells whole cells with OD_600_ of 1, 2 and 3. The enzymatic reactions were performed in 1.5 mL glass vials sealed with PTFE septum, with a reaction volume of 1 mL using 100 mM sodium phosphate buffer at pH 7.0. The reactions were incubated at 35 °C. The samples were taken after 15 h and analysed by HPLC to determine the conversions.

#### Time conversion profile using the optimized procedure

The *Sc*FDC1-catalyzed reactions were performed with the entire substrate panel (**1a–x**) under the optimal reaction conditions. The enzymatic reactions were performed in 1.5 mL glass vials sealed with PTFE septum, with a reaction volume of 1 mL using 100 mM sodium phosphate buffer at pH 7.0, 2 mM substrate concentration and *Sc*FDC1-containing whole cells with OD_600_ of ~1. The reactions were incubated at 35 °C. Samples for the time conversion profile were taken after 1, 2, 3, 4, 8, 24, 30, 48, 72 hours.

In case of the reactions of substrates **1b,e,h,i,j,n,o,q,s,t,u** after reaching the stationary phase of conversions (Fig. 3c,d, Table 1) the reactions were supplemented with additional batch of fresh cells (OD_600_ = 1) and were monitored by HPLC for an additional 24 h.

#### Site-directed mutagenesis

The site-directed mutagenesis was performed following the protocol described by Naismith and Liu^30^. The PCR reaction contained 2–4 ng of template DNA (recombinant plasmid pCDFDuet_*Sc*FDC1), 1 μM solution of primer pair (Table S4, entry 1–8), 200 μM dNTPs and 3 units of Pfu DNA polymerase, filled up to 50 μL with water. The PCR cycles were initiated at 95 °C for 5 min, followed by 15 amplification cycles. Each amplification cycle consisted of 95 °C for 1 min, at temperature of T_m_^no^−5 °C for 1 min and 72 °C for 15 min. The PCR cycles were finished with an annealing step at T_m_^pp^−5 °C for 1 min and the final extension step at 72 °C for 30 min. The PCR products were treated with 5 units of *Dpn*I at 37 °C for 2 h and then 10 μL of each PCR reactions was analyzed by agarose gel electrophoresis. An aliquot of 3 μL from the above PCR product was transformed into 100 μL suspension of *E. coli* XL1-Blue competent cells (OD_600_ 2.2) by heat shock. The transformed cells were spread on a Luria-Bertani (LB) plate containing streptomycin (25 µg/mL) and tetracyclin (12.5 µg/mL) and incubated at 37 °C for 16 h. 2–4 Colonies from each plate were grown and their plasmid DNAs were isolated. To verify the mutations, 400 ng of each extracted plasmid DNA was mixed with 50 pmol of sequencing primers (Table S4, entry 9–11) in a final volume of 15 μL. DNA sequencing was carried out using the sequencing service of Cemia (Larissa, Greece). The plasmids containing the envisaged mutations were transformed into *E. coli* BL21(DE3) pLysS host cells and further used in biotransformations.

### Computational studies

Ground state geometries of substrates **1a**–**x** were obtained at the DFT level of theory, employing the B3LYP functional and the 6–311++G(d,p) basis set. Harmonic vibrational frequencies, obtained at the same level of theory, confirmed that the stationary points are true local minima. All DFT calculations were performed using the Gaussian 09 package^31^.

Molecular docking was performed using the structure of ligand-bound FDC1 from *Aspergillus niger* (PDB code: 4ZA7)^6^, based on the high structural similarity of the active residues of *Sc*FDC1 and *An*FDC1 (Fig. S71). The presence of *pr*FMN cofactor and α-methyl cinnamate in *An*FDC1 supports an active conformation, compared to the impaired *Sc*FDC1 structures (*i*) without cofactor^32^ (PDB code: 4S13), (*ii*) with the catalytically essential glutamate E285 in inactive conformation^6^ (PDB code: 4ZAC) or (*iii*) of mutant E285D (PDB code: 6EVF) with 37 fold decreased k_cat_ value^25^, and (*iv*) with mutation R175A (PDB code: 6EVE), altering hydrogen bonding network implied in substrate fixation and hence inactivating the enzyme^32^. The search space was defined as a cubic box centered at the binding site, with an edge length of 20 Å. This grid box also incorporates a large surface pocket, where the best binding modes of large substrates were observed. Therefore, smaller grid boxes ranging from 10 to 16 Å were also used in docking studies to restrict the search space to the catalytic site and find favourable, although energetically higher, binding poses. Autodock Vina^33^ version 1.1.2 was used to perform rigid receptor docking. The center of the grid box was obtained from the central atom of the co-crystalized α-Me-cinnamic acid within the structure of *An*FDC1 (PDB code: 4ZA7)^6^.

Cofactor-substrate covalent intermediates geometries were evaluated for substrates **1a**,**v**,**w** using the B3LYP/6–31 G(d,p), including the D3 Grimme’s dispersion correction with Becke-Johnson damping^34^ to account for stacking interactions. In case of the covalent adduct between **1a** and prFMN, the geometry was refined with the B3LYP/6–311++G(d,p) and the obtained result was in excellent agreement with the reported data^6^.

## Conclusions

Our study exploring the substrate scope of *Sc*FDC1 using different cinnamic acid analogues revealed that the large cavity of the enzyme active site accepts, besides the –OCH_3_, –CF_3_ or –Br-substituted cinnamic acids, several bulky biaryl or heteroaryl substrates, as well as styrylacrylate.

Computational studies indicated that substrate planarity is beneficial for the decarboxylation reaction and is determined by the narrow active site as well as by the formation of the 1,3-cycloaddition adduct with the prFMN cofactor. It was shown that substrate preference of *Sc*FDC1 was further determined by a channel bottlenecked by gatekeeper residues residues Q192 and I330 and also by the limited volume of the substrate-binding pocket, restricting the access of bulky, non-linear substrates.

The results demonstrate that whole-cells of *E. coli* harbouring the *fdc1* gene are efficient catalysts for the production of a wide variety of styrene derivatives, furthermore display the substrate profile of FDC1 and provide perspectives for the rational design driven expansion of its substrate tolerance.

## Supplementary information

Supplementary Information
