# Supplementary material for: Exploring the substrate scope of ferulic acid decarboxylase (FDC1) from Saccharomyces cerevisiae
Source: Sci Rep. 2019 Jan 24;9:647. doi: 10.1038/s41598-018-36977-x (PMC6345843; doi:10.1038/s41598-018-36977-x)
Supplement: Supplementary file 1 — Supplementary Information [file 41598_2018_36977_MOESM1_ESM.docx]

**Electronic Supplementary Information**

**Exploring the substrate scope of ferulic acid decarboxylase (FDC1) from *Saccharomyces cerevisiae***

Emma Zsófia Aletta Nagy, Levente Csaba Nagy, Alina Filip, Katalin Nagy, Emese Gál, Róbert Tőtős, László Poppe, Csaba Paizs, László Csaba Bencze

**Table of Contents**

[1. Results of initial screening experiments 3](#_Toc525287527)

[2. Cromatographic data and representative HPLC and GC-MS results 4](#_Toc525287528)

[2.1. GC-MS experimental parameters 4](#_Toc525287529)

[2.2. HPLC methods 4](#_Toc525287530)

[2.3. Representative HPLC chromatograms from the separation of 1a-x and anisole 5](#_Toc525287531)

[2.4. GC-MS chromatograms of the FDC1 catalyzed decarboxylation reaction for demonstrating the formation of styrenes as product 17](#_Toc525287532)

[3. Synthesis of acrylic acid derivatives 1a-x 40](#_Toc525287533)

[4. The effect of pH upon the conversion values of biotransformation of 1i 44](#_Toc525287534)

[5. Molecular docking 45](#_Toc525287535)

[7. Site-directed mutagenesis 49](#_Toc525287536)

# 1. Results of initial screening experiments

**Table S1.** *Sc*FDC1-containing whole-cell biotransformations of **1a-x**: conversions from initial screening, after 24 h reaction time

| Substrate | | c* (%) |
| --- | --- | --- |
| cinnamic acid | **1a** | 75 |
| (*E*)-3-(2-bromophenyl)acrylic acid | **1b** | 80 |
| (*E*)-3-(3-bromophenyl)acrylic acid | **1c** | >99 |
| (*E*)-3-(4-bromophenyl)acrylic acid | **1d** | >99 |
| (*E*)-3-(2-methoxyphenyl)acrylic acid | **1e** | 82 |
| (*E*)-3-(3-methoxyphenyl)acrylic acid | **1f** | 92 |
| (*E*)-3-(4-methoxyphenyl)acrylic acid | **1g** | 86 |
| (*E*)-3-(2-(trifluoromethyl)phenyl)acrylic acid | **1h** | 42 |
| (*E*)-3-(3-(trifluoromethyl)phenyl)acrylic acid | **1i** | 80 |
| (*E*)-3-(4-(trifluoromethyl)phenyl)acrylic acid | **1j** | 69 |
| (2*E*,4*E*)-5-phenylpenta-2,4-dienoic acid | **1k** | 95 |
| (*E*)-5-phenylpent-2-enoic acid | **1l** | <1 |
| (*E*)-3-(naphthalen-2-yl)acrylic acid | **1m** | 96 |
| (*E*)-3-(quinolin-2-yl)acrylic acid | **1n** | 31 |
| (*E*)-3-(quinolin-4-yl)acrylic acid | **1o** | 33 |
| (*E*)-3-(benzofuran-2-yl)acrylic acid | **1p** | >99 |
| (*E*)-3-(benzofuran-3-yl)acrylic acid | **1q** | 75 |
| (*E*)-3-(5-chlorobenzofuran-2-yl)acrylic acid | **1r** | 93 |
| (*E*)-3-([1,1'-biphenyl]-4-yl)acrylic acid | **1s** | 26 |
| (*E*)-3-(4'-fluoro-[1,1'-biphenyl]-4-yl)acrylic acid | **1t** | 48 |
| (*E*)-3-(5-phenylthiophen-2-yl)acrylic acid | **1u** | 82 |
| (*E*)-3-(5-(4-bromophenyl)furan-2-yl)acrylic acid | **1v** | <1 |
| (*E*)-3-(2-phenylthiazol-4-yl)acrylic acid | **1w** | <1 |
| (*E*)-3-(10-methyl-10H-phenothiazin-2-yl)acrylic acid | **1x** | <1 |

# 2. Cromatographic data and representative HPLC and GC-MS results

## 2.1. GC-MS experimental parameters

**Table S2.** GC-MS experimental parameters**.**

| **Parameter** | **Setting** |
| --- | --- |
| Carrier gas | Helium (6.0) |
| GC inlet, split | 250 °C, split ratio=20:1, constant flow (rate= 1.5 mL/min) |
| GC-column | HP-5MS crosslinked 5% phenyl methylsiloxane film  (30 m × 0.25 mm, 0.25 µm film) |
| GC temperature program | 40°C (5) − 220°C (5) at 10°C/min |
| GC-MS injector temperature | 250 °C |
| MS ionization | electron impact |
| MS full scanning mass range | 35−500 amu |

## 2.2. HPLC methods

The HPLC methods for conversion determination of the FDC1 catalyzed enzymatic reactions were performed on Agilent 1200 and/or 1260 series high performance liquid chromatography (HPLC) using Gemini NX-C18 150x4.5 mm or Zorbax SB-C8 50x2.1 mm columns, flow rate: 1mL/min. Quantification of the conversion was based on determining the consumption of the acrylic acid substrates **2a**-**x** using anisol as internal standard.

**Table S3.** HPLC methods used for the conversion value determinations

| **Compound** | **Eluent*[% B]** | **Retention time (min)** | | | **Wavelength used for UV detection** |
| --- | --- | --- | --- | --- | --- |
|  |  | **1a-x** | | **anisole** |  |
| **1a**** | 0 to 100 in 13 min | 9.1 | 10.5 | | 280 |
| **1b** | 0 to 55 in 12 min | 9.3 | 8.4 | | 270 |
| **1c** | 0 to 55 in 12 min | 9.6 | 8.4 | | 270 |
| **1d** | 0 to 55 in 12 min | 9.6 | 8.4 | | 270 |
| **1e**** | 0 to 85 in 13 min | 10.3 | 11.6 | | 270 |
| **1f**** | 0 to 85 in 13 min | 10.3 | 11.5 | | 270 |
| **1g**** | 0 to 85 in 13 min | 10 | 11.5 | | 270 |
| **1h** | 0 to 55 in 12 min | 9.7 | 8.5 | | 270 |
| **1i** | 0 to 55 in 12 min | 10.1 | 8.5 | | 270 |
| **1j** | 0 to 55 in 12 min | 10.1 | 8.5 | | 270 |
| **1k** | 0 to 55 in 12 min | 9.3 | 8.3 | | 230 |
| **1l** | 0 to 55 in 12 min | 9.3 | 8.3 | | 230 |
| **1m** | 0 to 55 in 12 min | 10.2 | 8.4 | | 270 |
| **1n**** | 0 to 80 in 10 min | 6.7 | 10.5 | | 270 |
| **1o**** | 0 to 80 in 10 min | 5.9 | 10.5 | | 270 |
| **1p** | 0 to 55 in 12 min | 9.4 | 8.4 | | 280 |
| **1q** | 0 to 55 in 12 min | 9.0 | 8.4 | | 270 |
| **1r** | 0 to 55 in 12 min | 10.7 | 8.5 | | 280 |
| **1s** | 0 to 55 in 12 min | 11.3 | 8.5 | | 270 |
| **1t** | 0 to 55 in 12 min | 11.4 | 8.4 | | 270 |
| **1u** | 0 to 55 in 12 min | 11.2 | 8.4 | | 250 |
| **1v** | 0 to 55 in 12 min | 11.9 | 8.4 | | 250 |
| **1w** | 0 to 55 in 12 min | 10.0 | 8.5 | | 250 |
| **1x** | 0 to 55 in 12 min | 11.7 | 8.4 | | 280 |

*Mobile phase: A: water + 0.1% v/v TFA / B: acetonitrile + 0.1% v/v TFA; flow rate: 1.0 mL min^-1^, measurements performed at 20 °C, using Zorbax SB-C8 (2.1 × 50mm; 5 µm); ** measurements performed at 25 °C, using Gemini NX-C18 column (150 × 4.5 mm; 5 µm).

## 2.3. Representative HPLC chromatograms from the separation of 1a-x and anisole


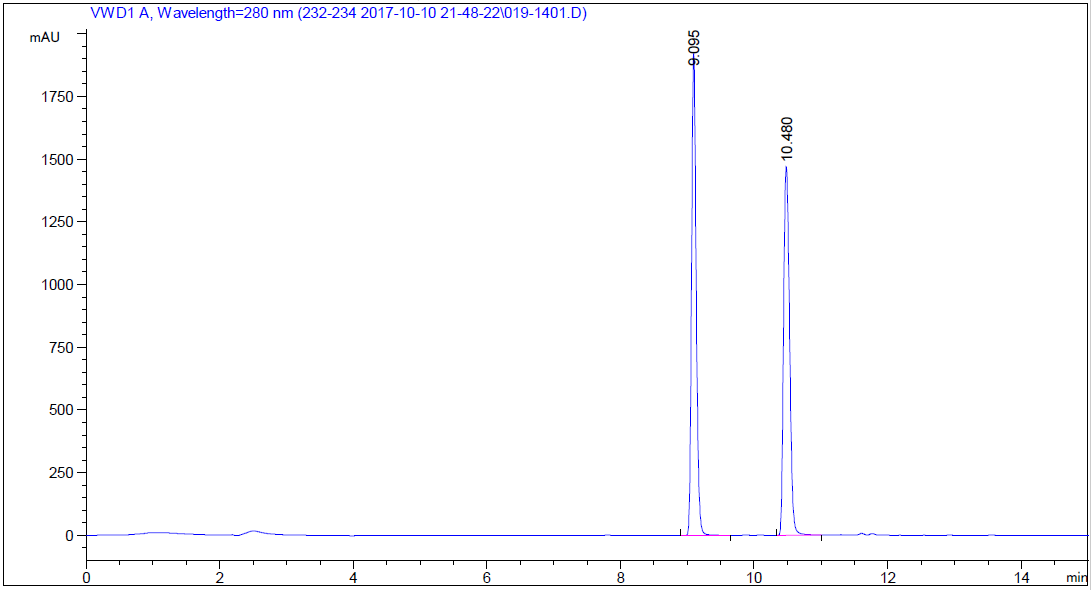


**Figure S1.** HPLC chromatogram from the separation of **1a** and **anisole**


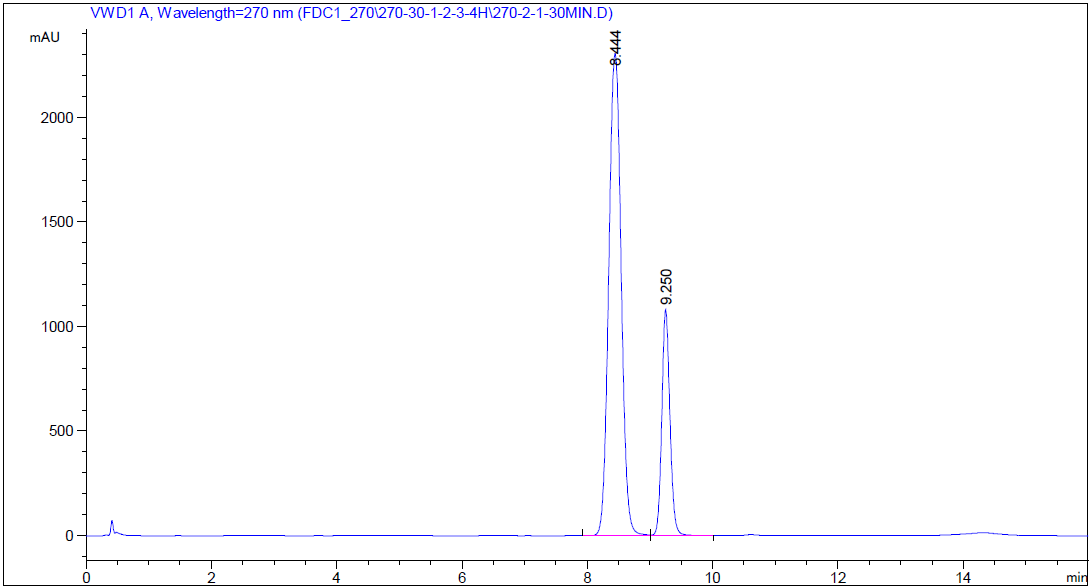


**Figure S2.** HPLC chromatogram from the separation of **1b** and **anisole**


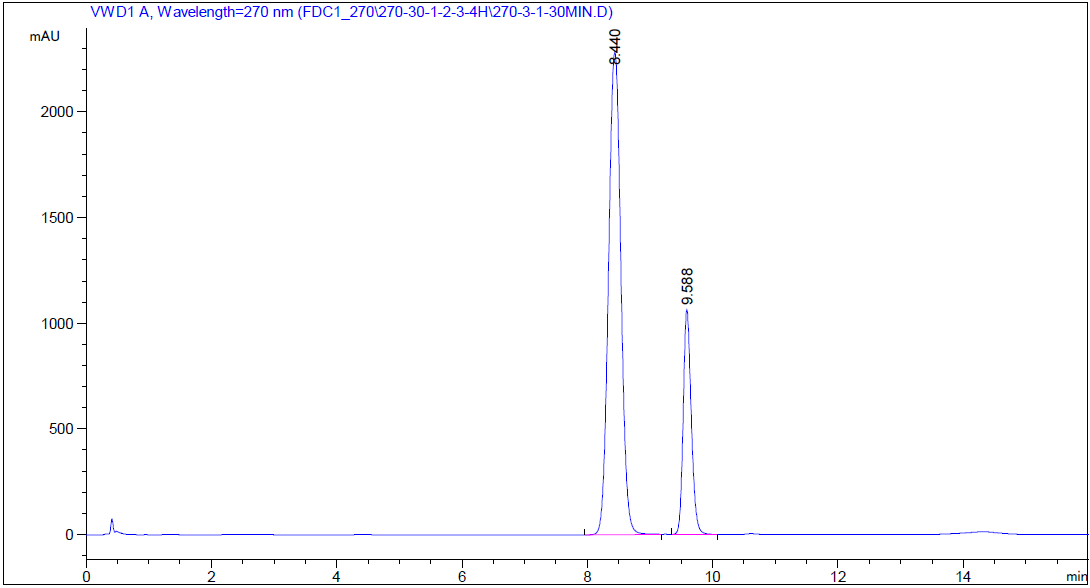


**Figure S3.** HPLC chromatogram from the separation of **1c** and **anisole**


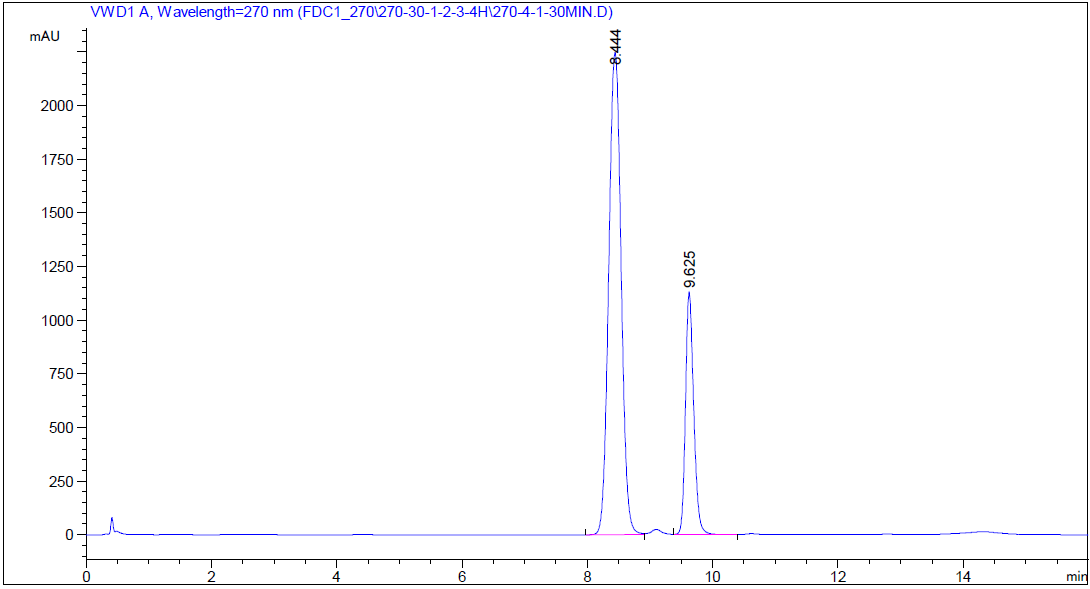


**Figure S4.** HPLC chromatogram from the separation of **1d** and **anisole**


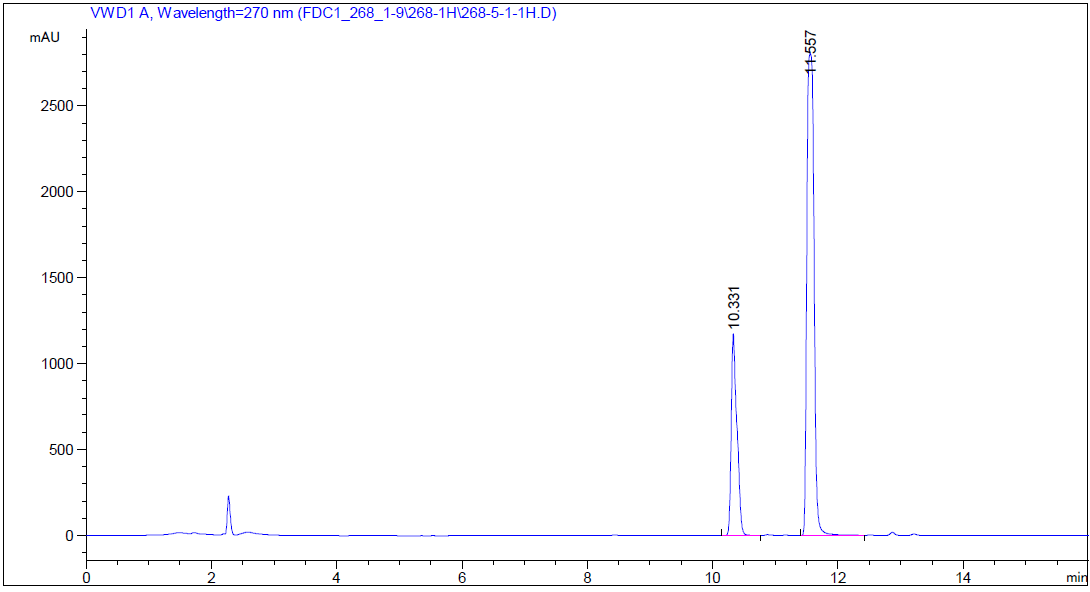


**Figure S5.** HPLC chromatogram from the separation of **1e** and **anisole**


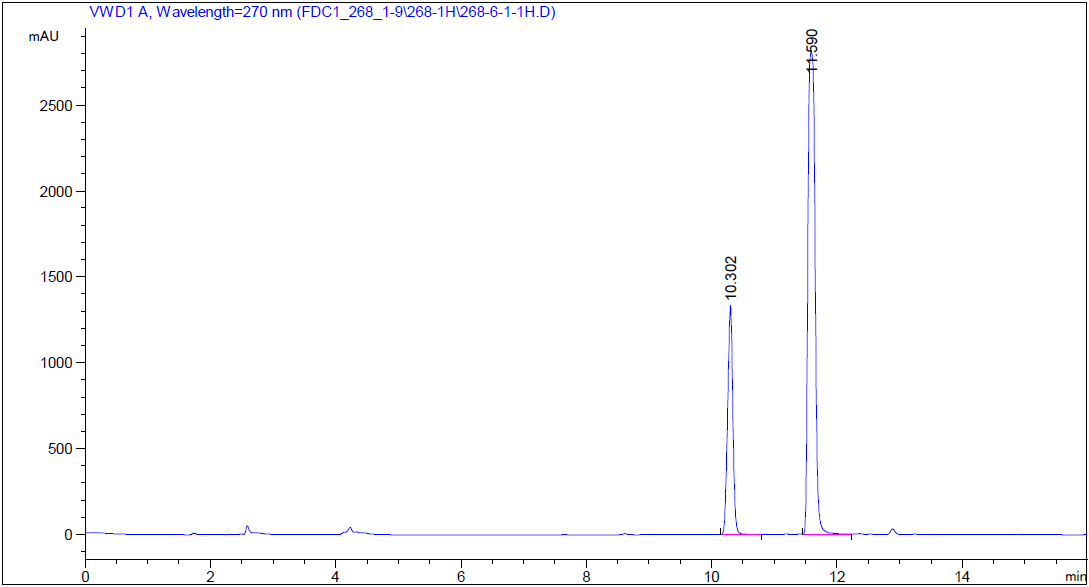


**Figure S6.** HPLC chromatogram from the separation of **1f** and **anisole**


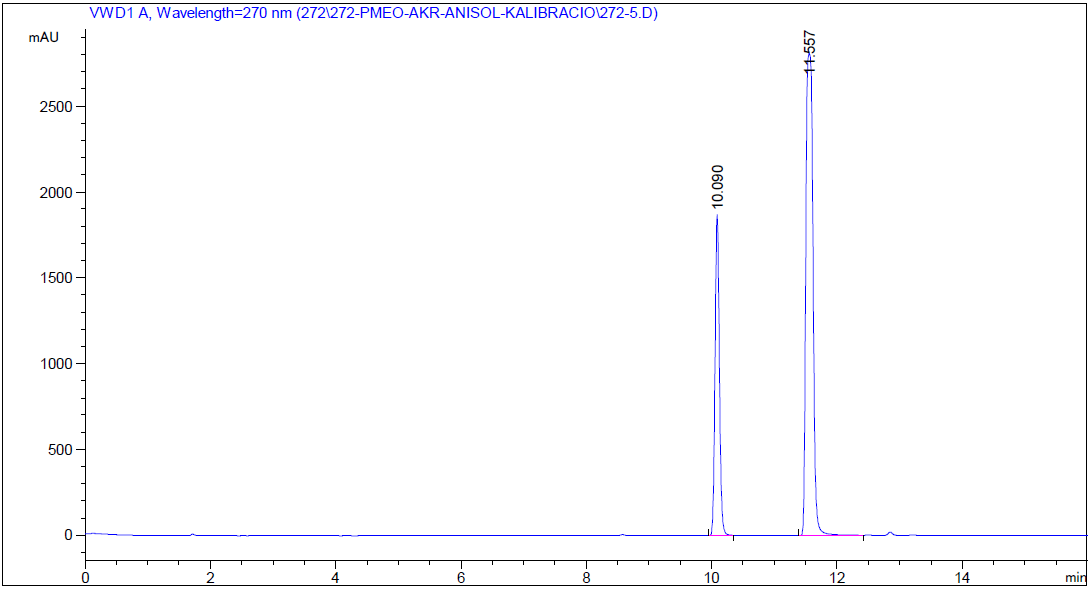


**Figure S7.** HPLC chromatogram from the separation of **1g** and **anisole**


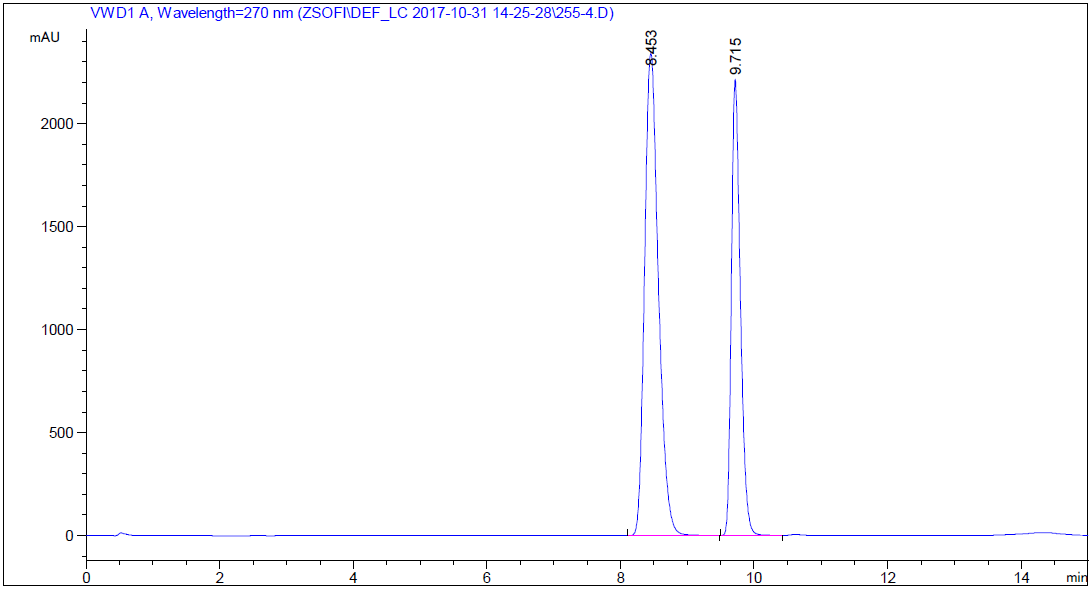


**Figure S8.** HPLC chromatogram from the separation of **1h** and **anisole**


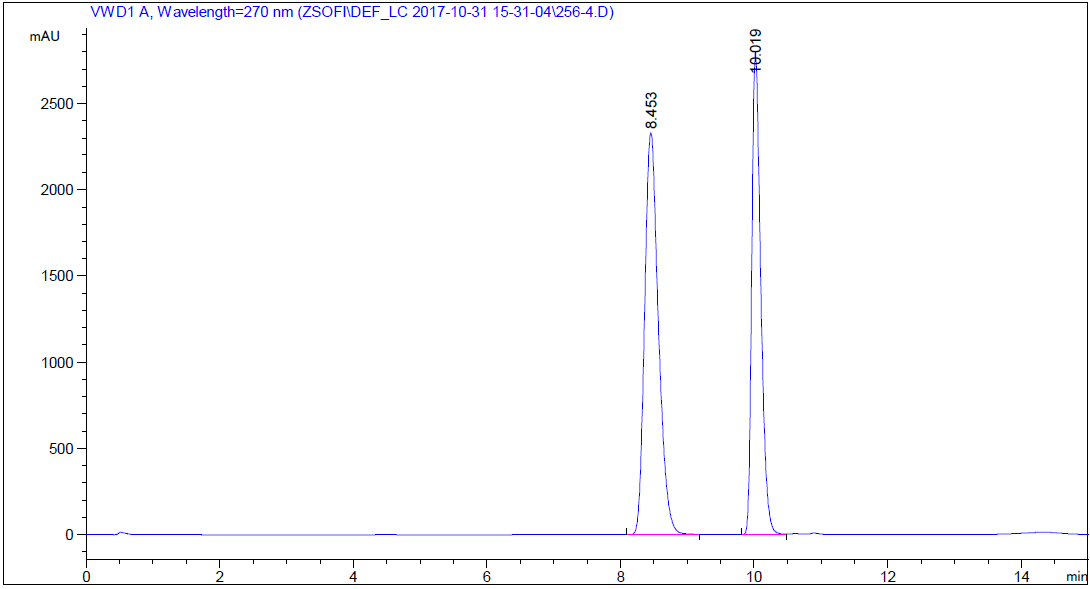


**Figure S9.** HPLC chromatogram from the separation of **1i** and **anisole**


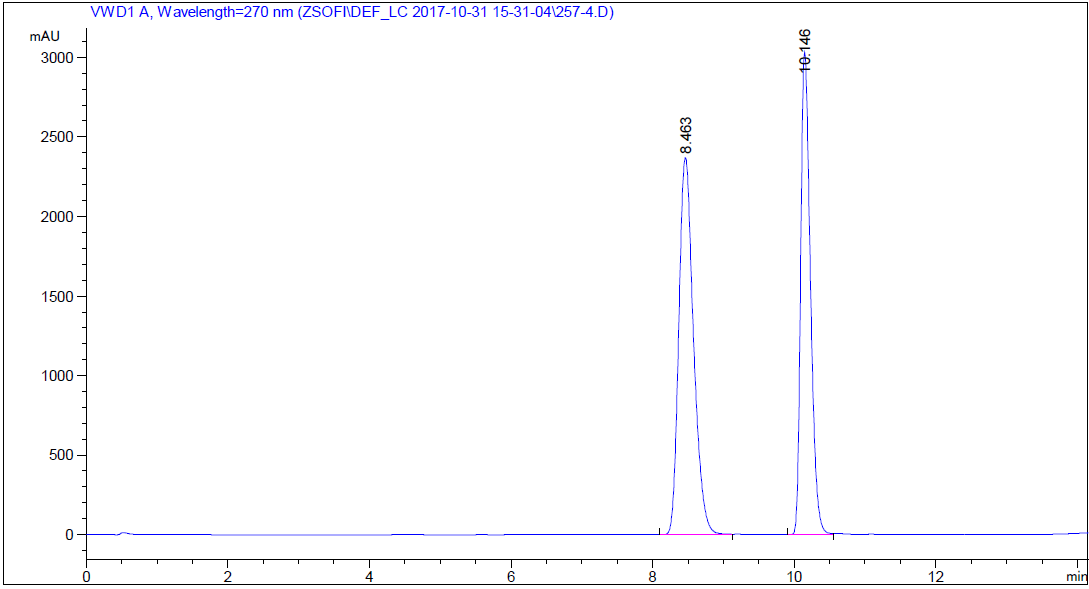


**Figure S10.** HPLC chromatogram from the separation of **1j** and **anisole**


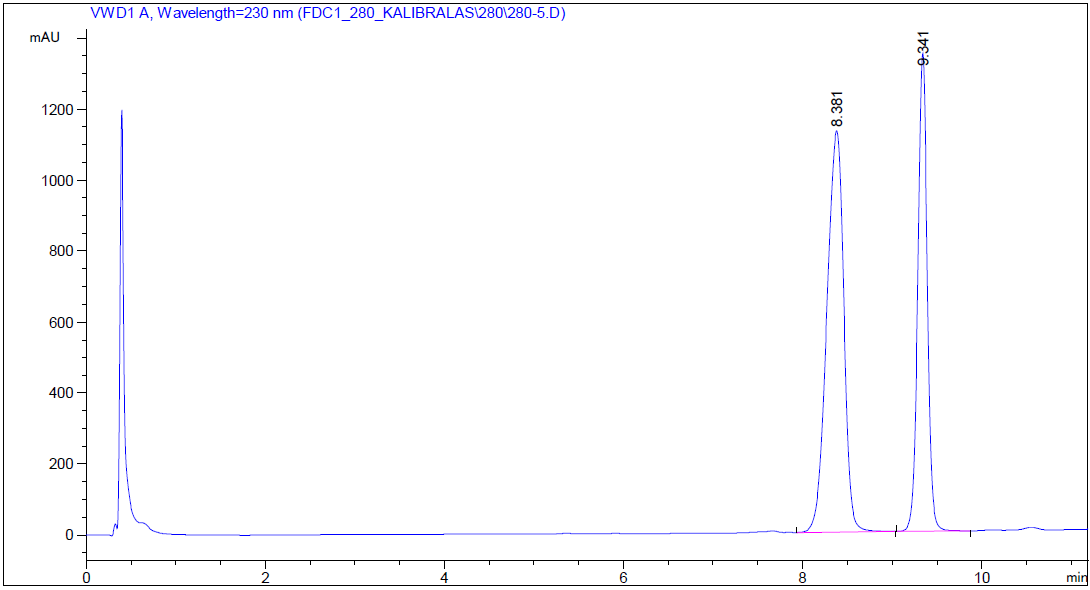


**Figure S11.** HPLC chromatogram from the separation of **1k** and **anisole**


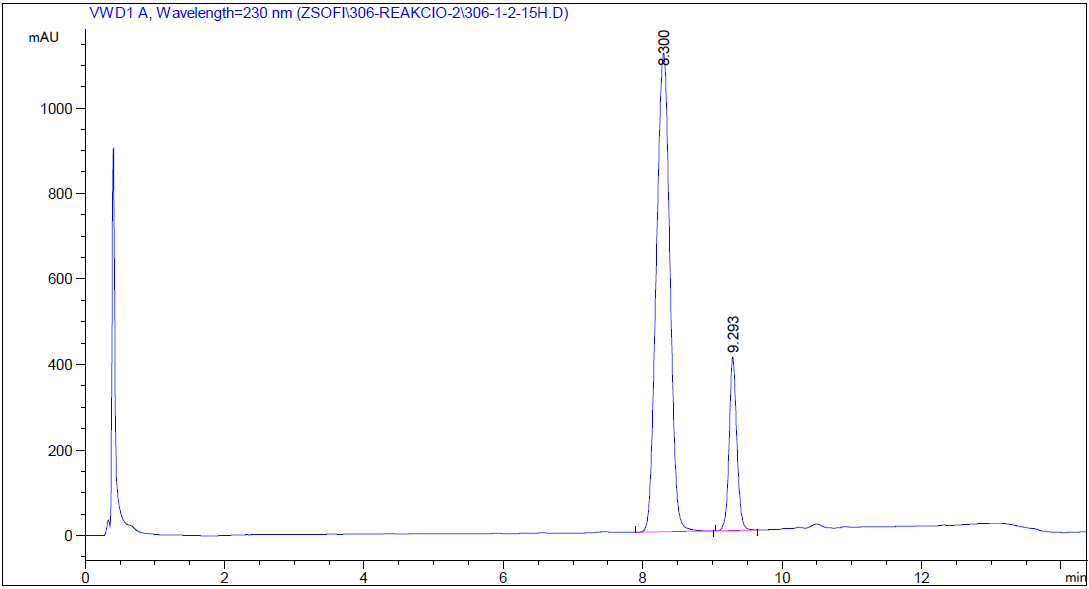


**Figure 12.** HPLC chromatogram from the separation of **1l** and **anisole**


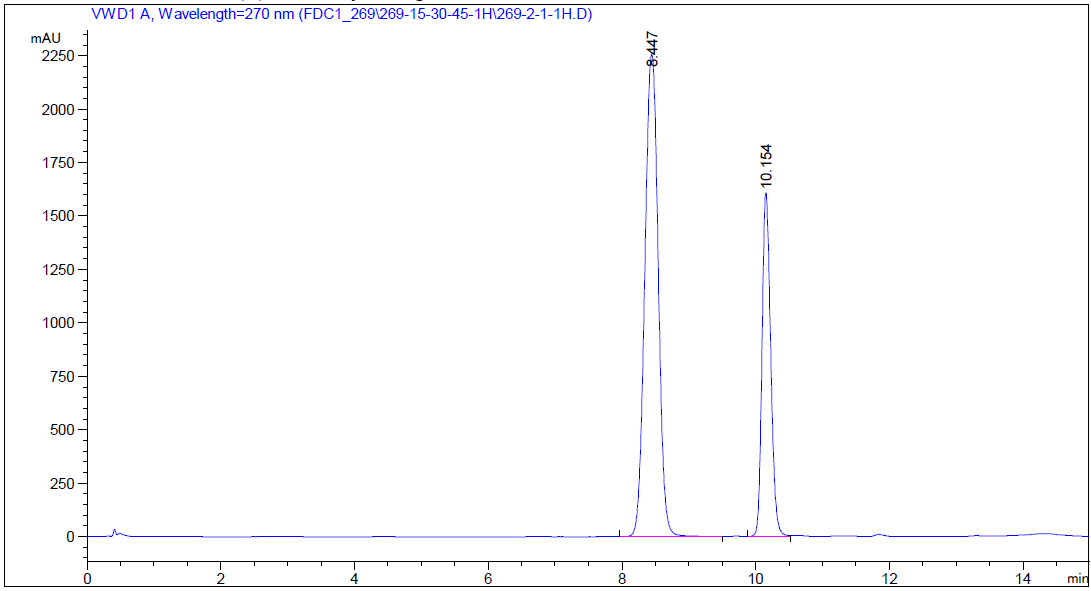


**Figure S13.** HPLC chromatogram from the separation of **1m** and **anisole**


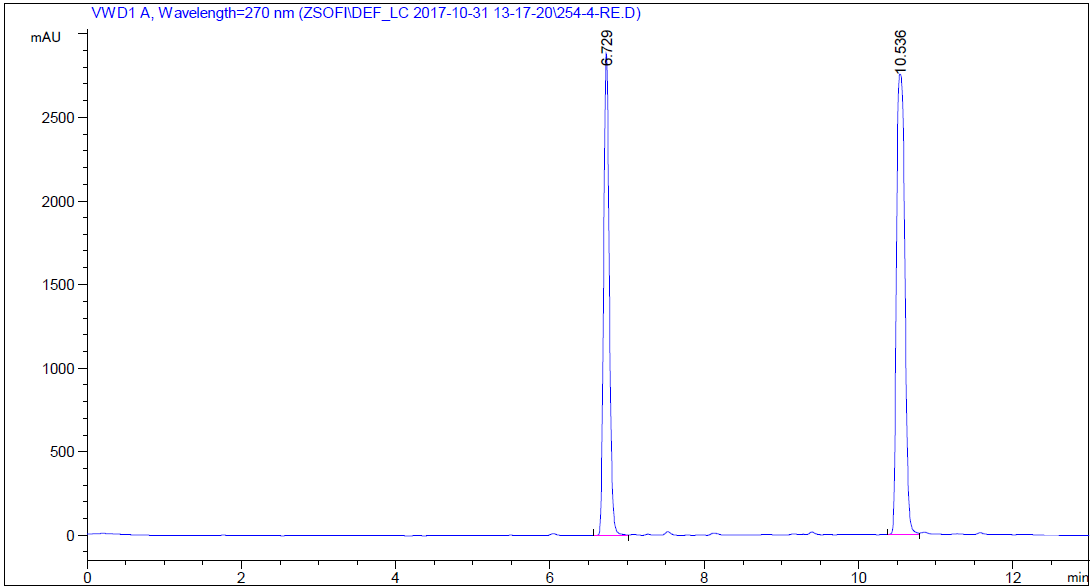


**Figure S14.** HPLC chromatogram from the separation of **1n** and **anisole**


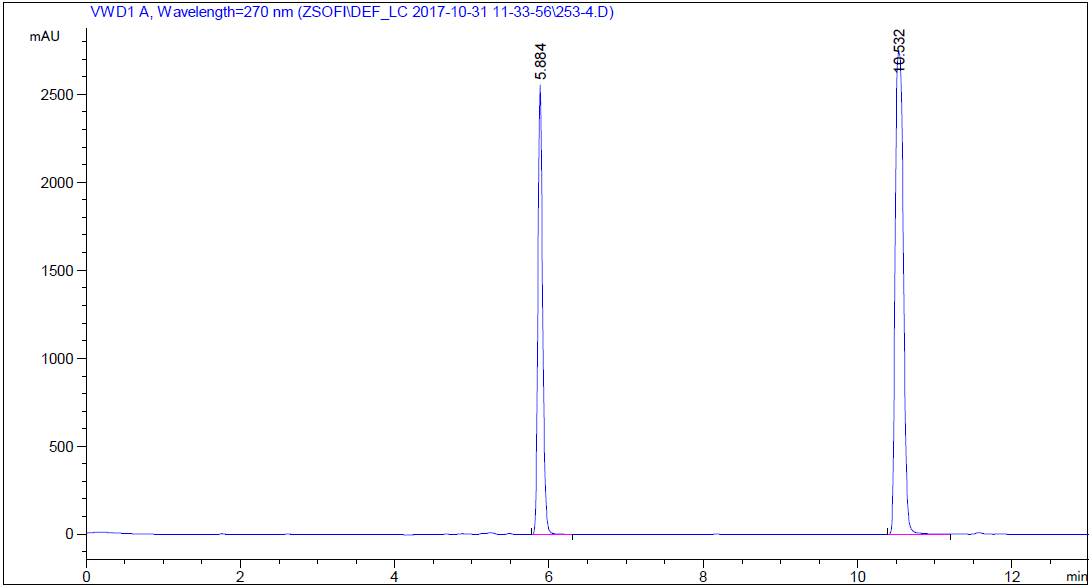


**Figure S15.** HPLC chromatogram from the separation of **1o** and **anisole**


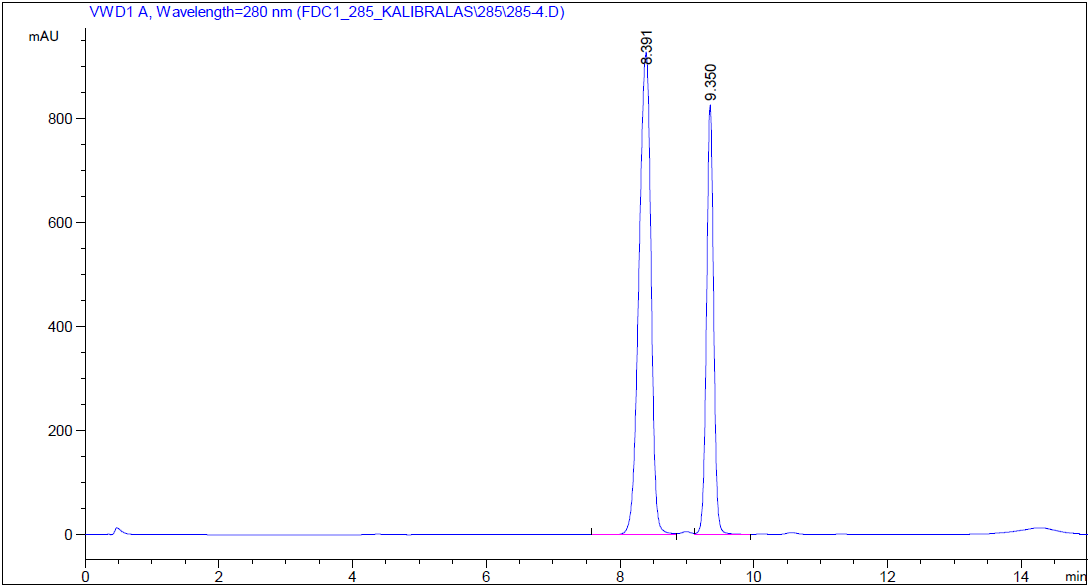


**Figure S16.** HPLC chromatogram from the separation of **1p** and **anisole**


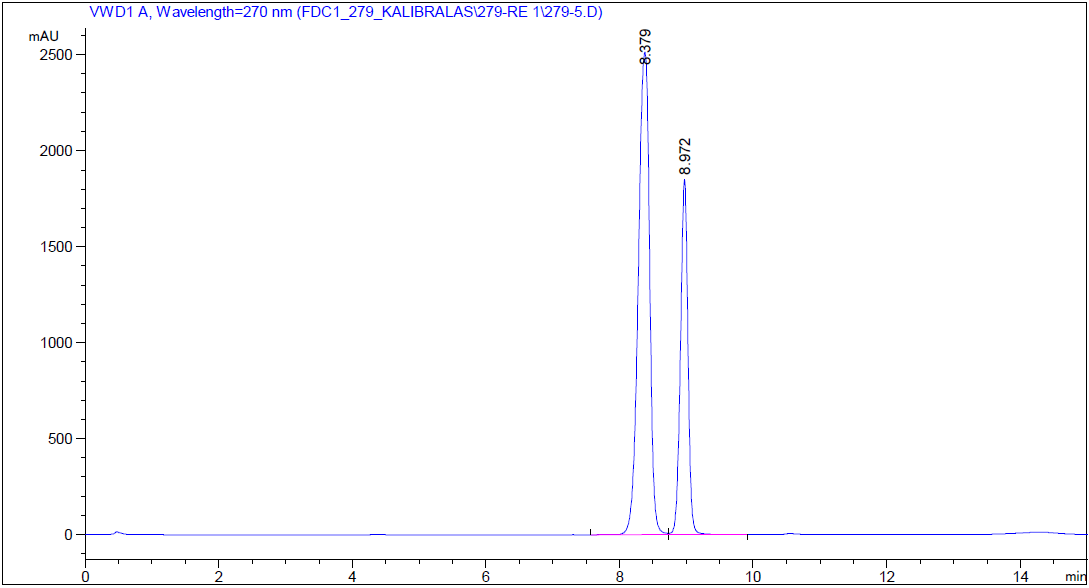


**Figure S17.** HPLC chromatogram from the separation of **1q** and **anisole**


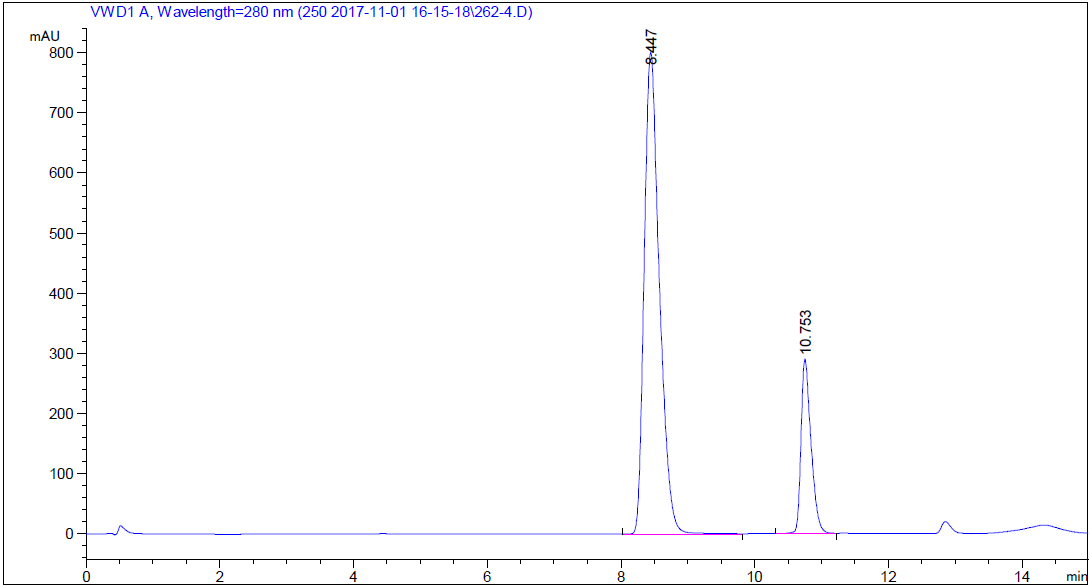


**Figure S18.** HPLC chromatogram from the separation of **1r** and **anisole**


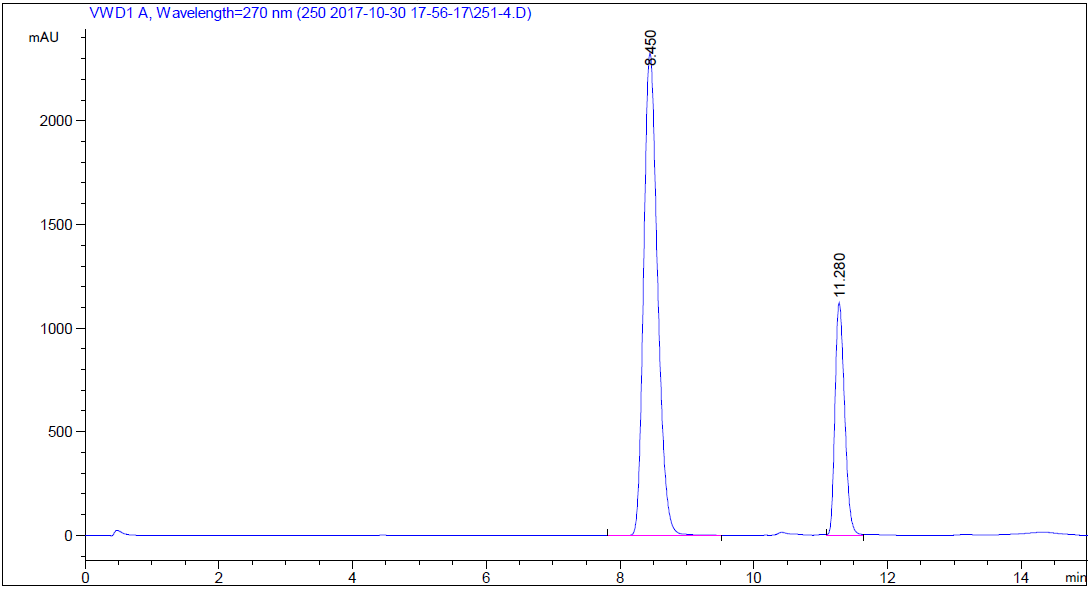


**Figure S19.** HPLC chromatogram from the separation of **1s** and **anisole**


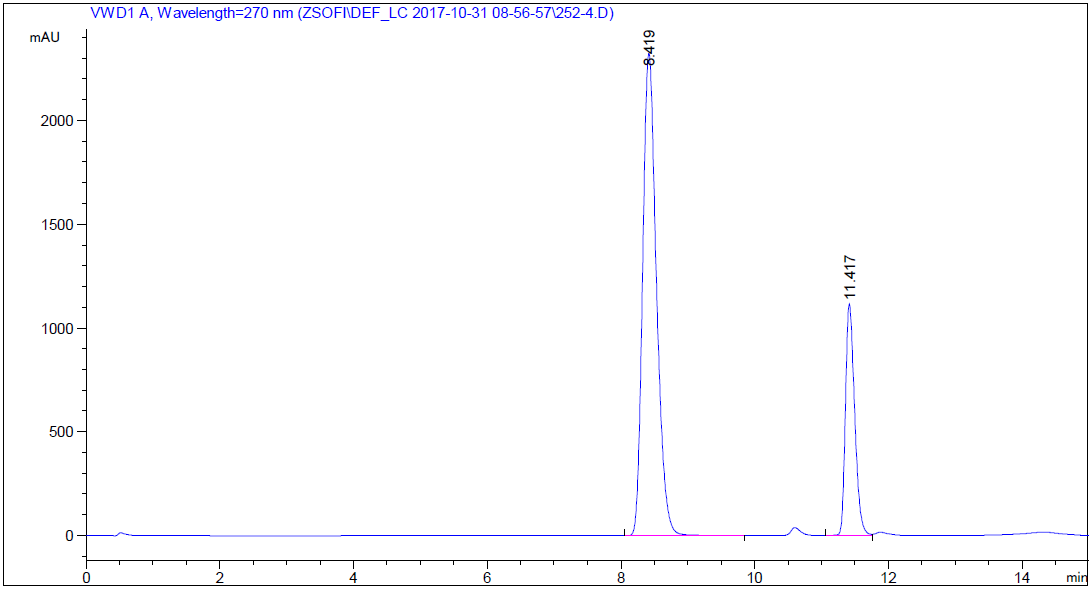


**Figure S20.** HPLC chromatogram from the separation of **1t** and **anisole**


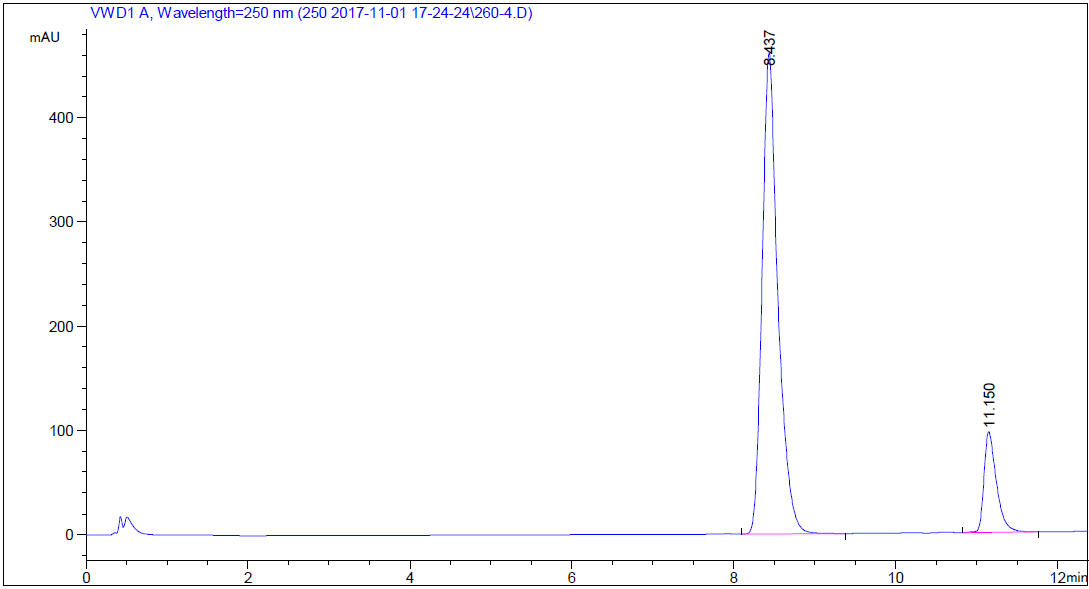


**Figure S21.** HPLC chromatogram from the separation of **1u** and **anisole**


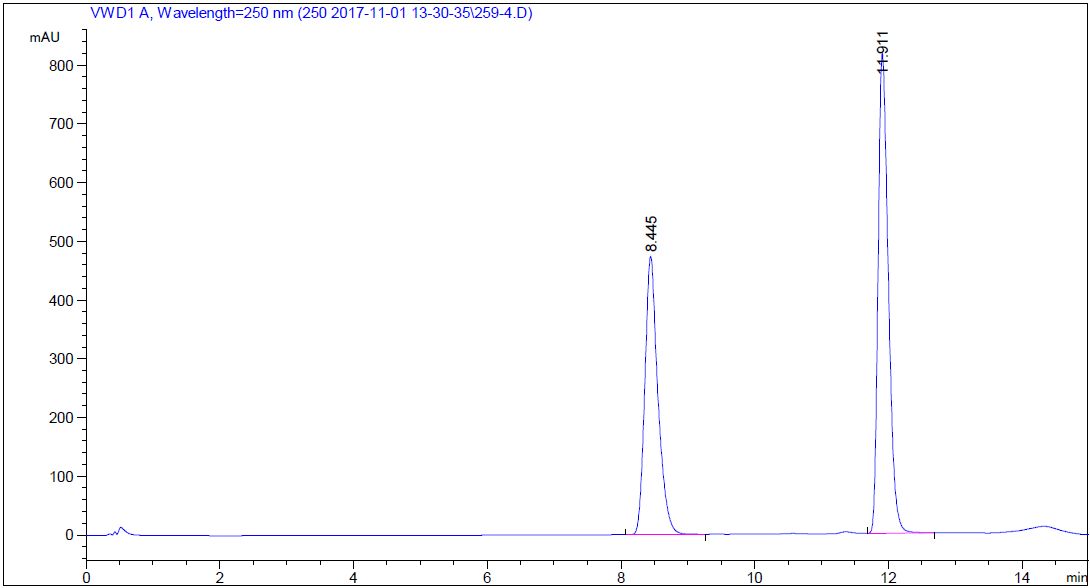


**Figure S22.** HPLC chromatogram from the separation of **1v** and **anisole**


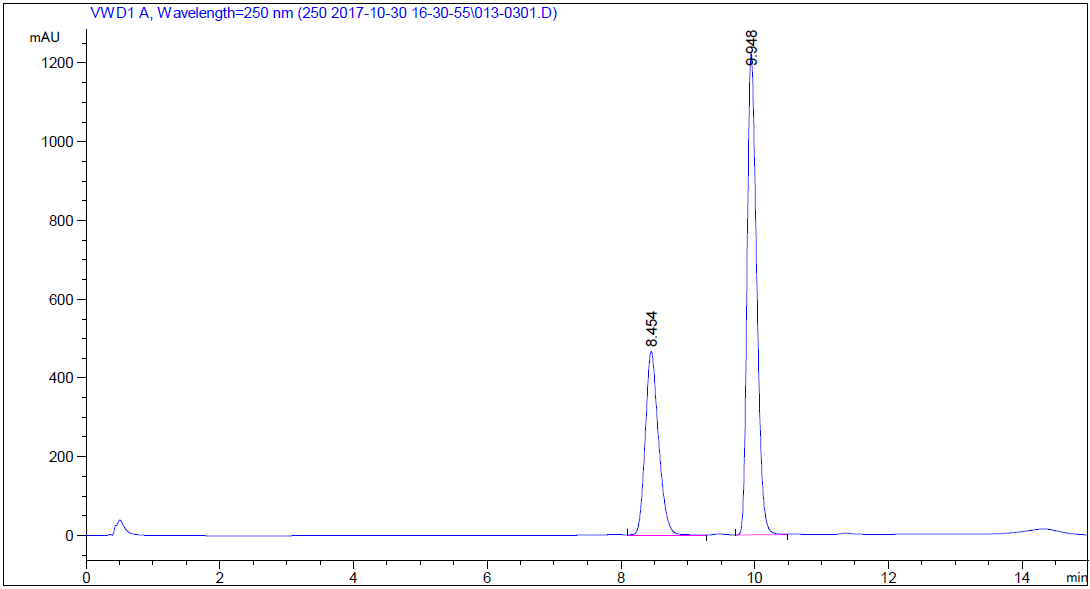


**Figure S23.** HPLC chromatogram from the separation of **1w** and **anisole**


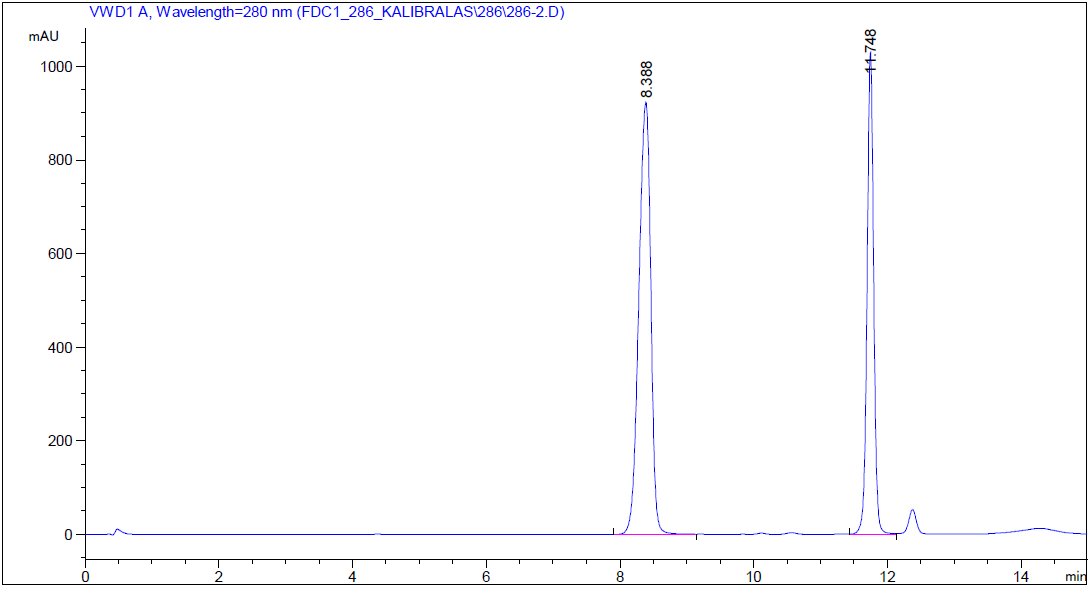


**Figure S24.** HPLC chromatogram from the separation of **1x** and **anisole**

## 2.4. GC-MS chromatograms of the FDC1 catalyzed decarboxylation reaction for demonstrating the formation of styrenes as product

The production of styrenes were confirmed through GC-MS analysis. The samples were prepared by extracting the biotransformations (see main manuscris section 3.4.) with *n*-hexane or *tert*-butyl methyl ether and dried on anhydrous sodium sulfate. The gas chromatography-mass spectrometry (GC-MS) analyses were performed using a Shimadzu QP 2010 PLUS Mass Spectrometer coupled with Gas Chromatograph (Shimadzu). The mass spectras were recorded in the electron impact mode (MS/EI) at 70 eV. Peak identification was carried out by analogy of mass spectra with those of the mass library (NIST 2.0 and Wiley).


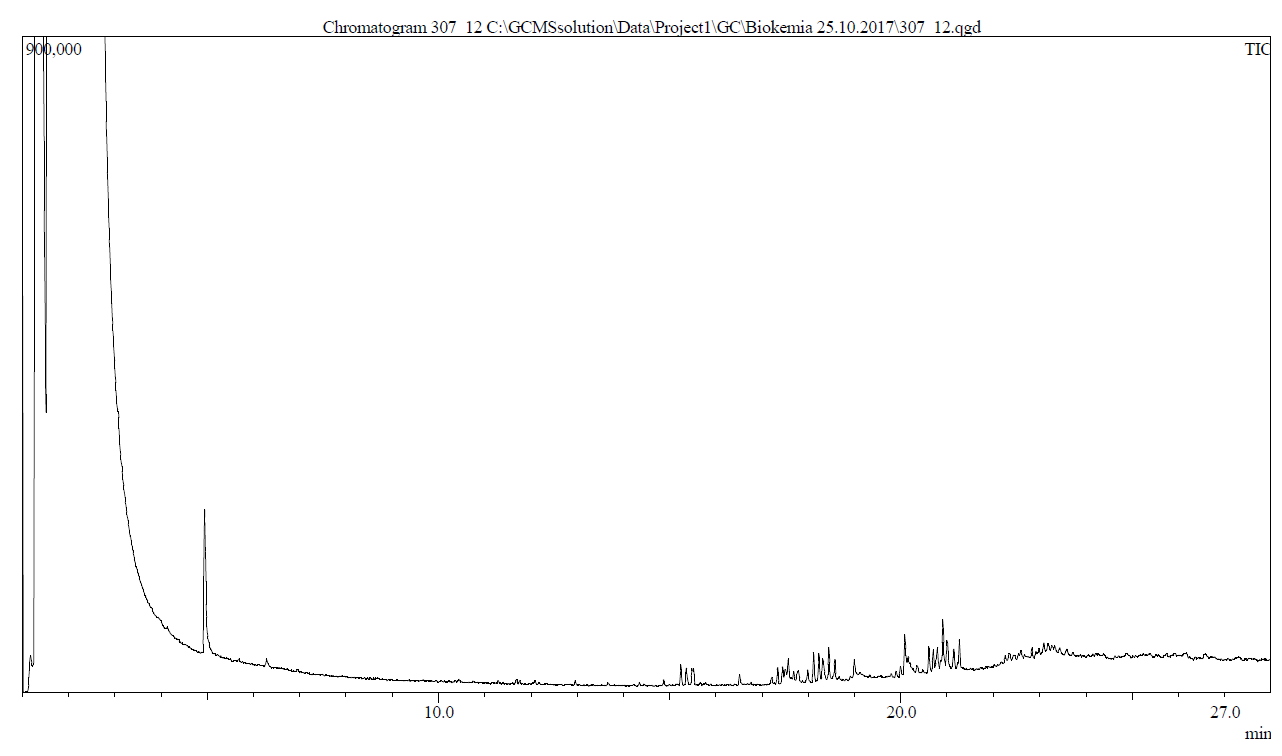


**Figure S25.** GC chromatogram of n-hexane extract of blank matrix (reaction mixture without substrate)


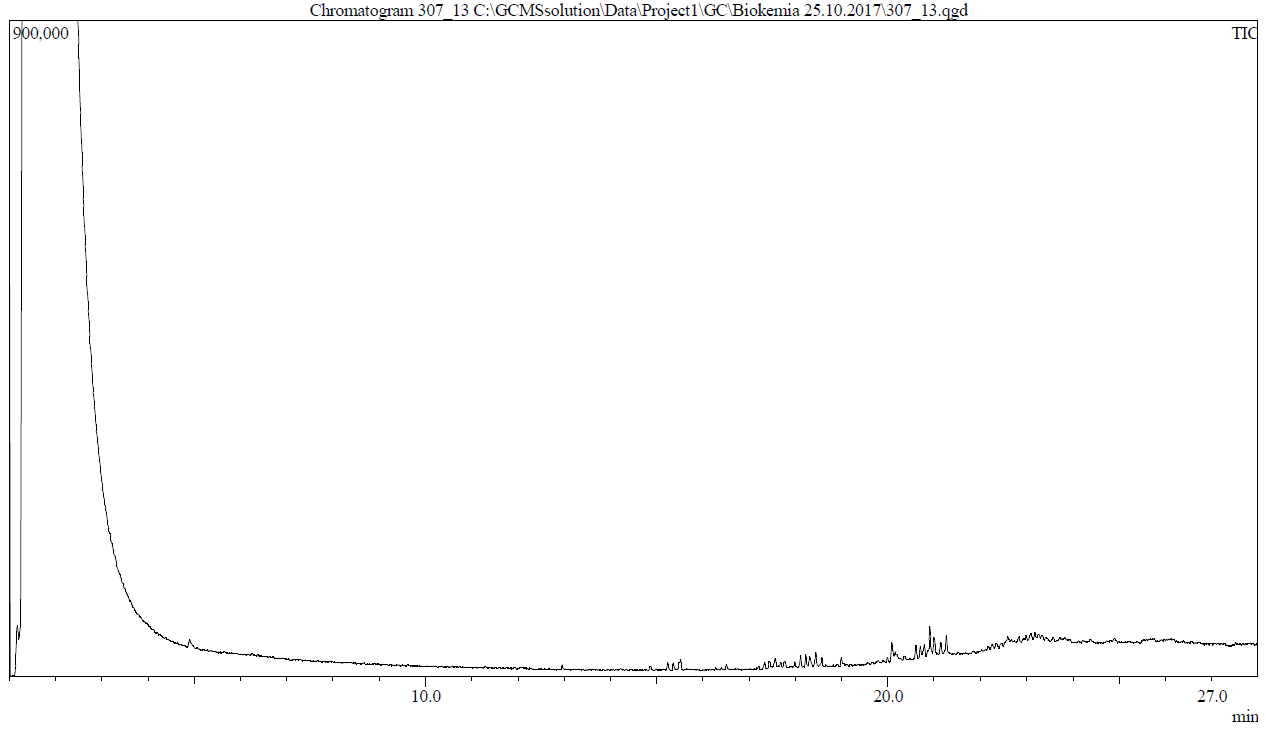


**Figure S26.** GC chromatogram of tert-butyl methyl ether extract of blank matrix (reaction mixture without substrate)


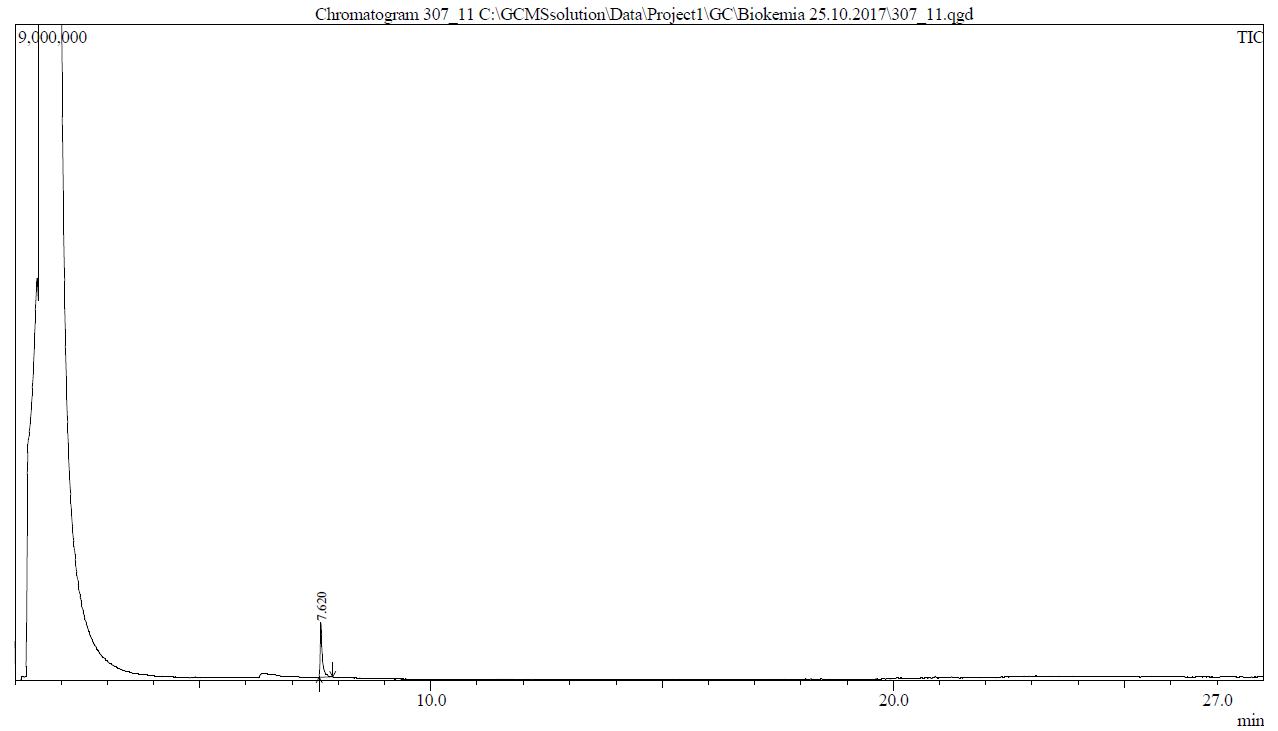


**Figure S27.** GC chromatogram of the styrene **2a** extracted with n-hexane


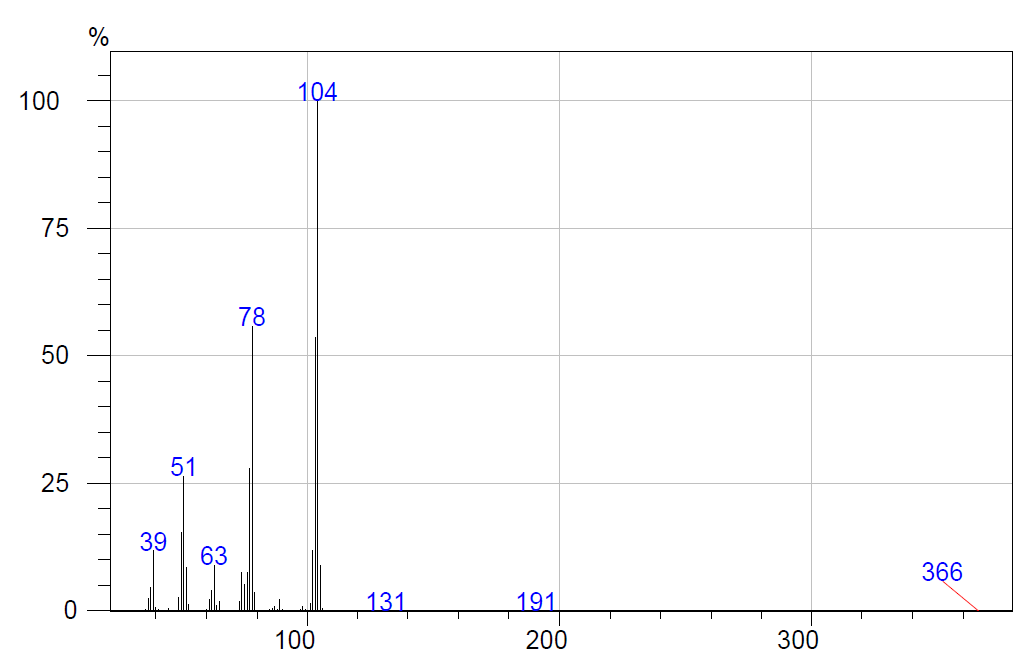

**Figure S28.** The MS spectrum for the product **2a**


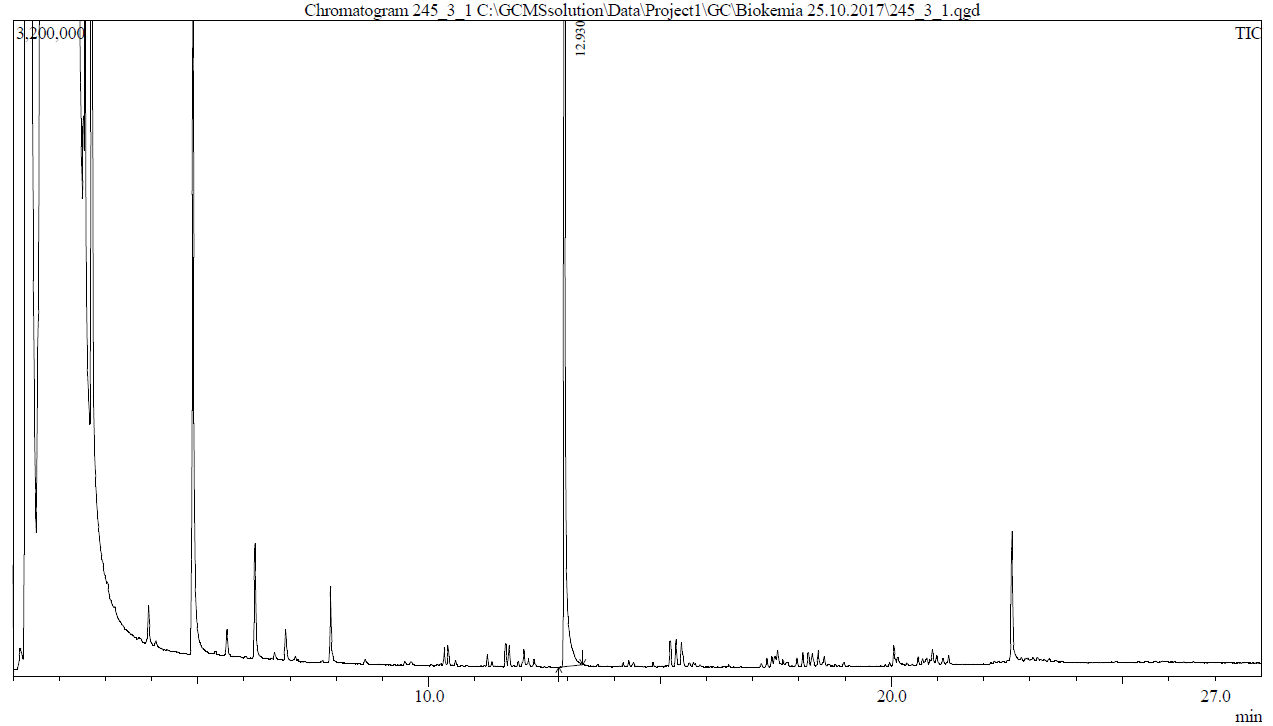


**Figure S29.** GC chromatogram of the styrene **2b** extracted with n-hexane


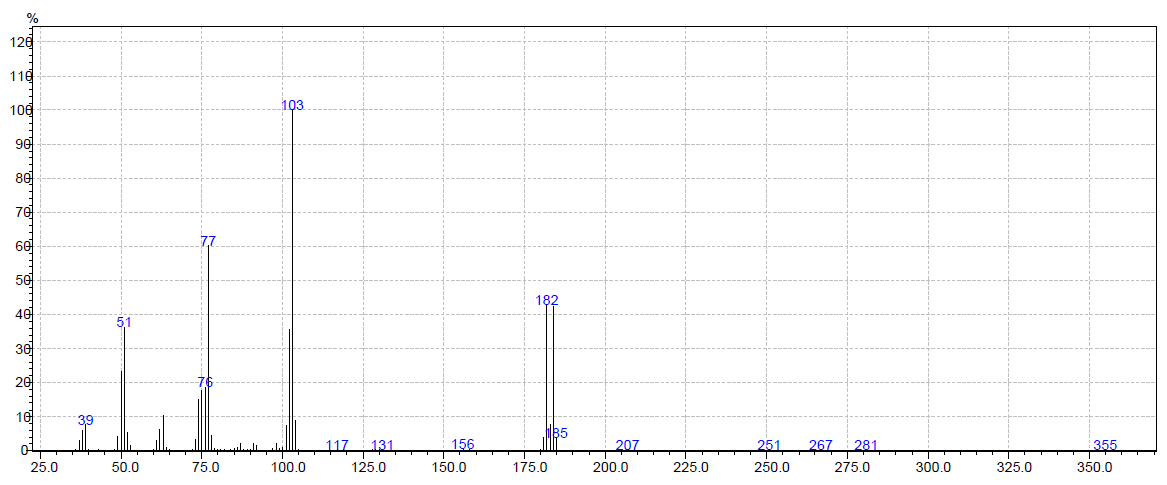

**Figure S30.** The MS spectrum for the product **2b**


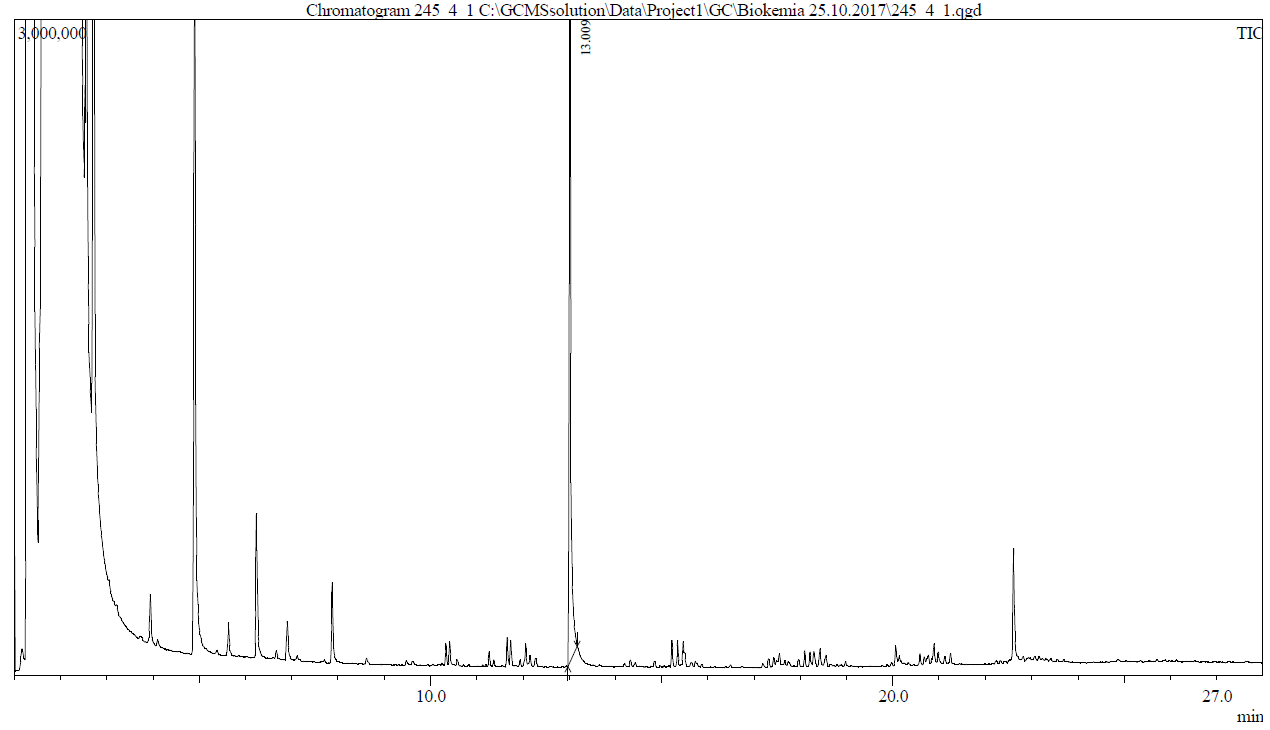


**Figure S31.** GC chromatogram of the styrene **2c** extracted with n-hexane


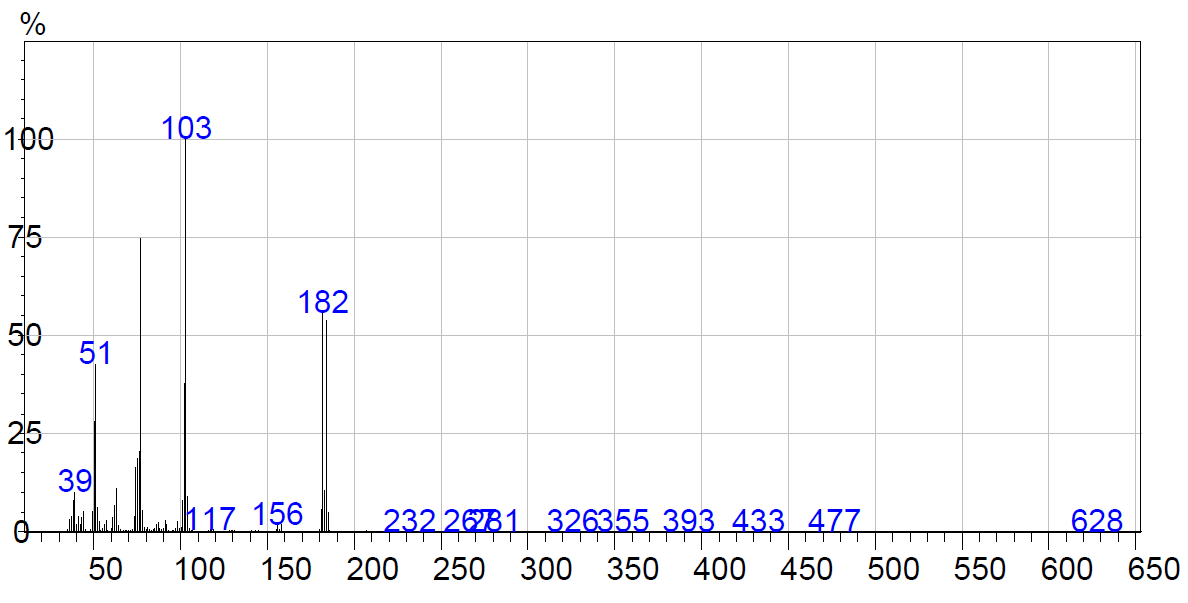

**Figure S32.** The MS spectrum for the product **2c**


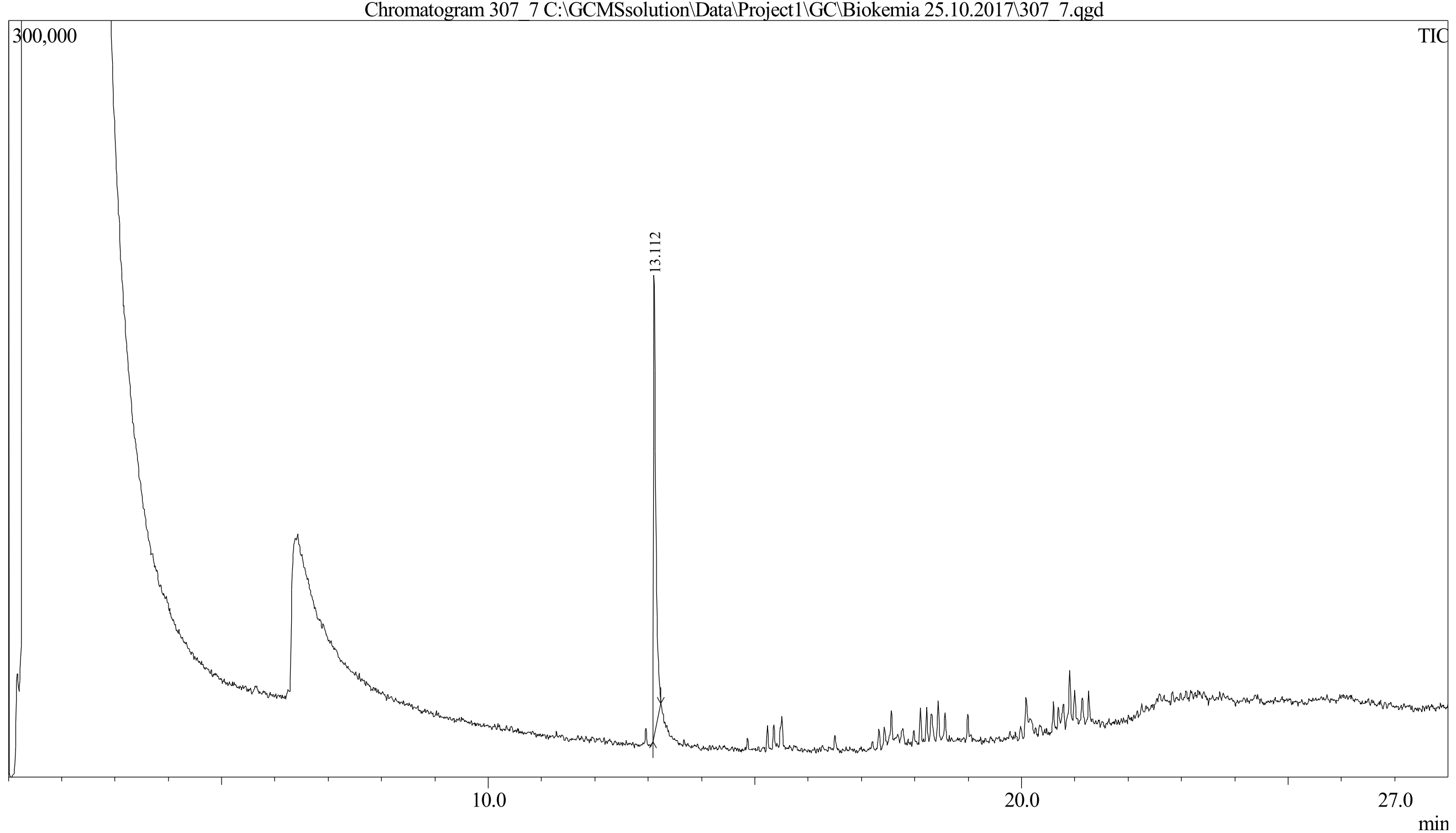


**Figure S33.** GC chromatogram of the styrene **2d** extracted with n-hexane


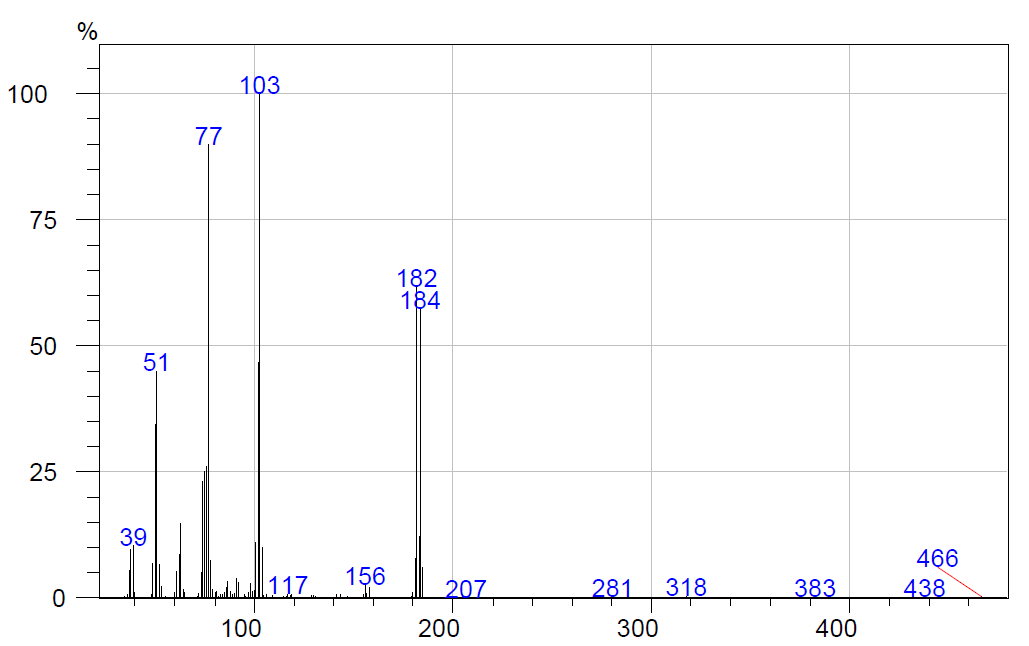

**Figure S34.** The MS spectrum for the product **2d**


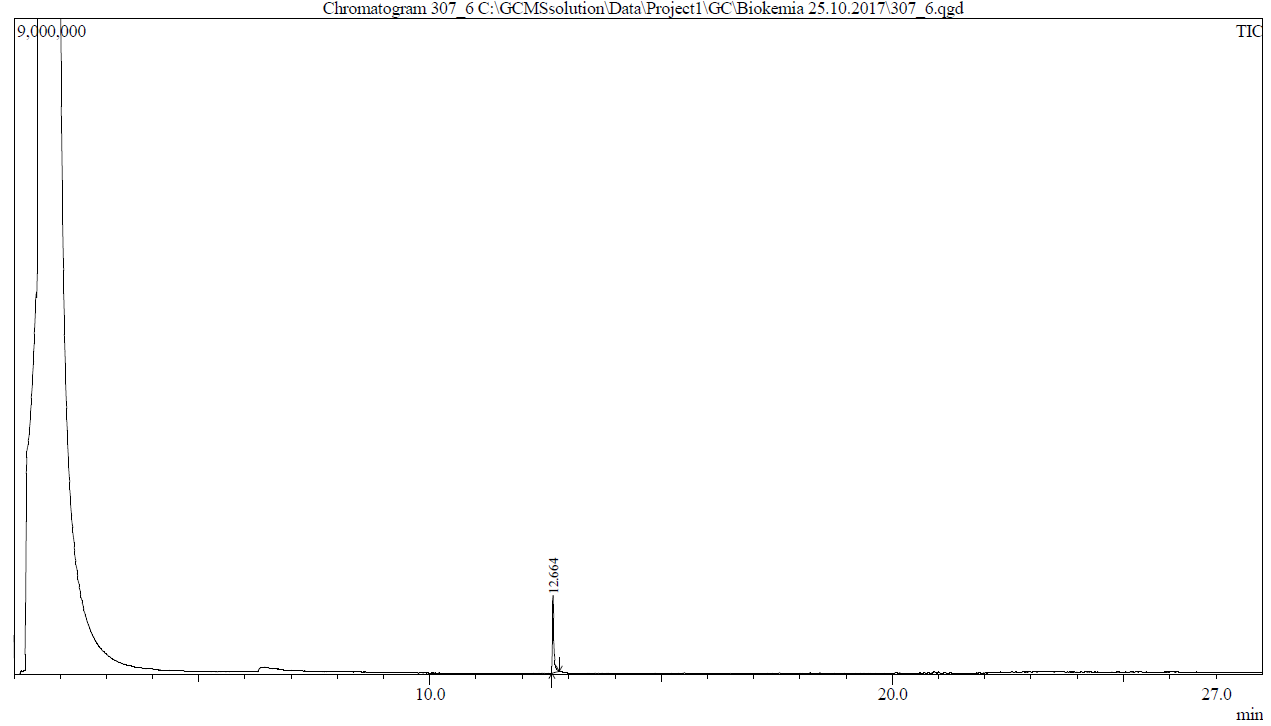


**Figure S35.** GC chromatogram of the styrene **2e** extracted with tert-butyl methyl ether


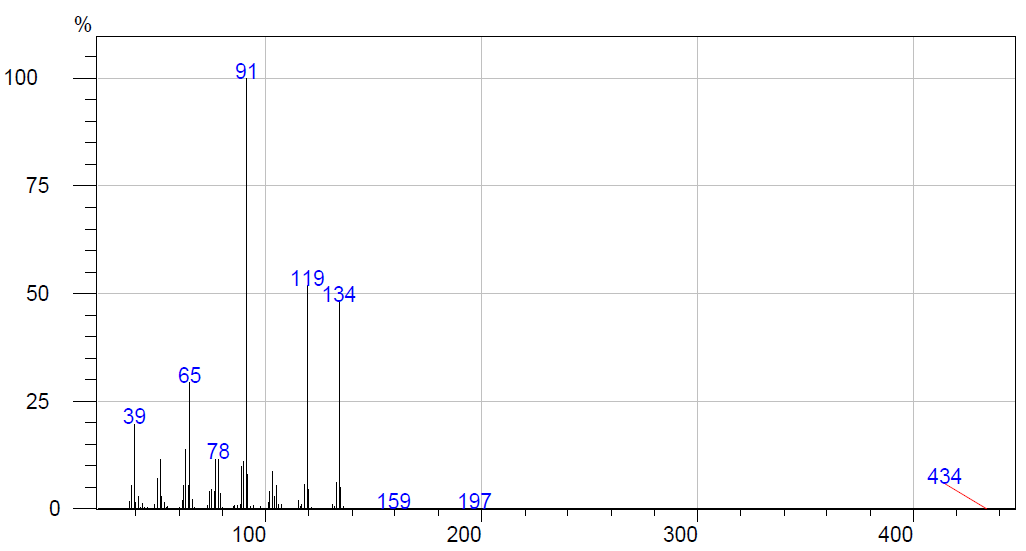

**Figure S36.** The MS spectrum for the product **2e**


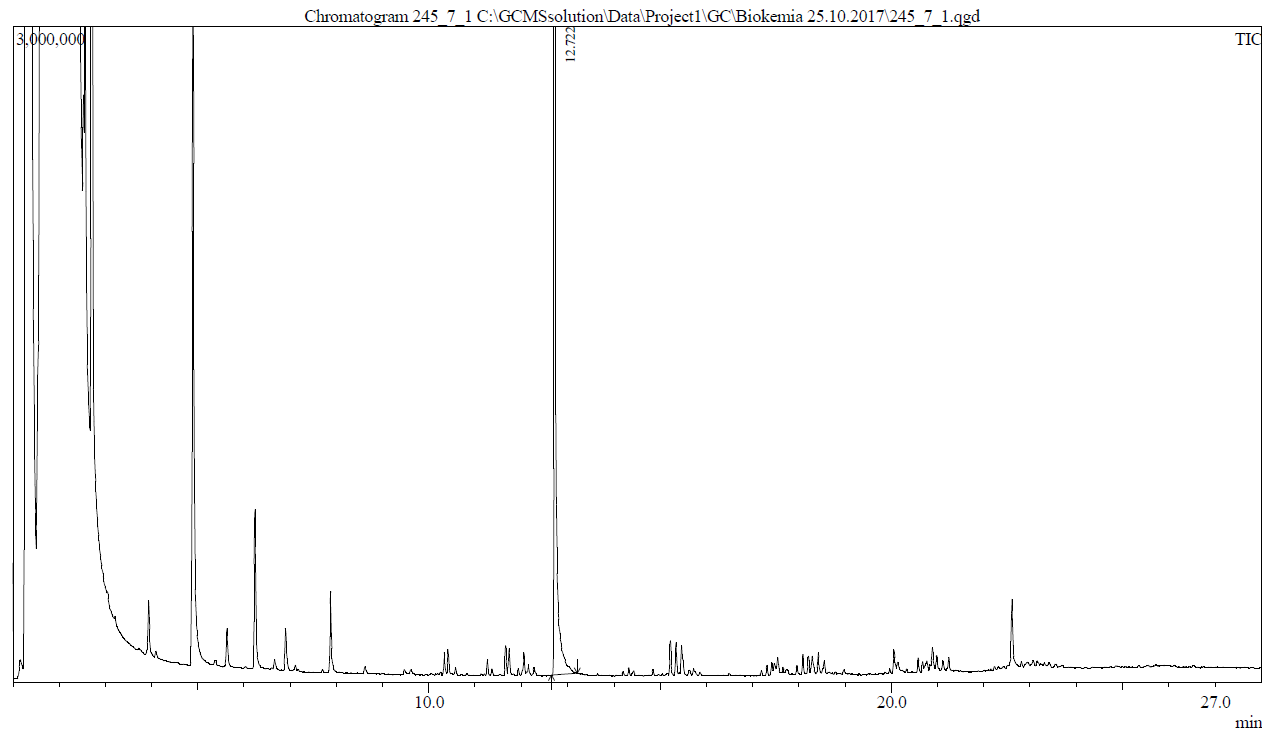


**Figure S37.** GC chromatogram of the styrene **2f** extracted with n-hexane


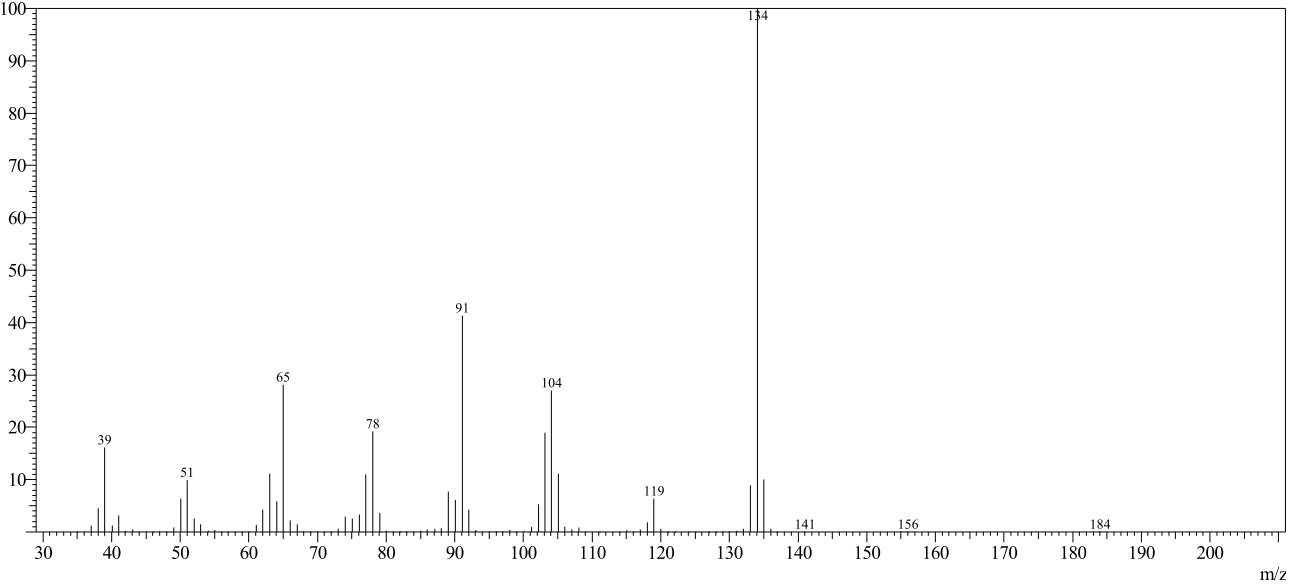

**Figure S38.** The MS spectrum for the product **2f**


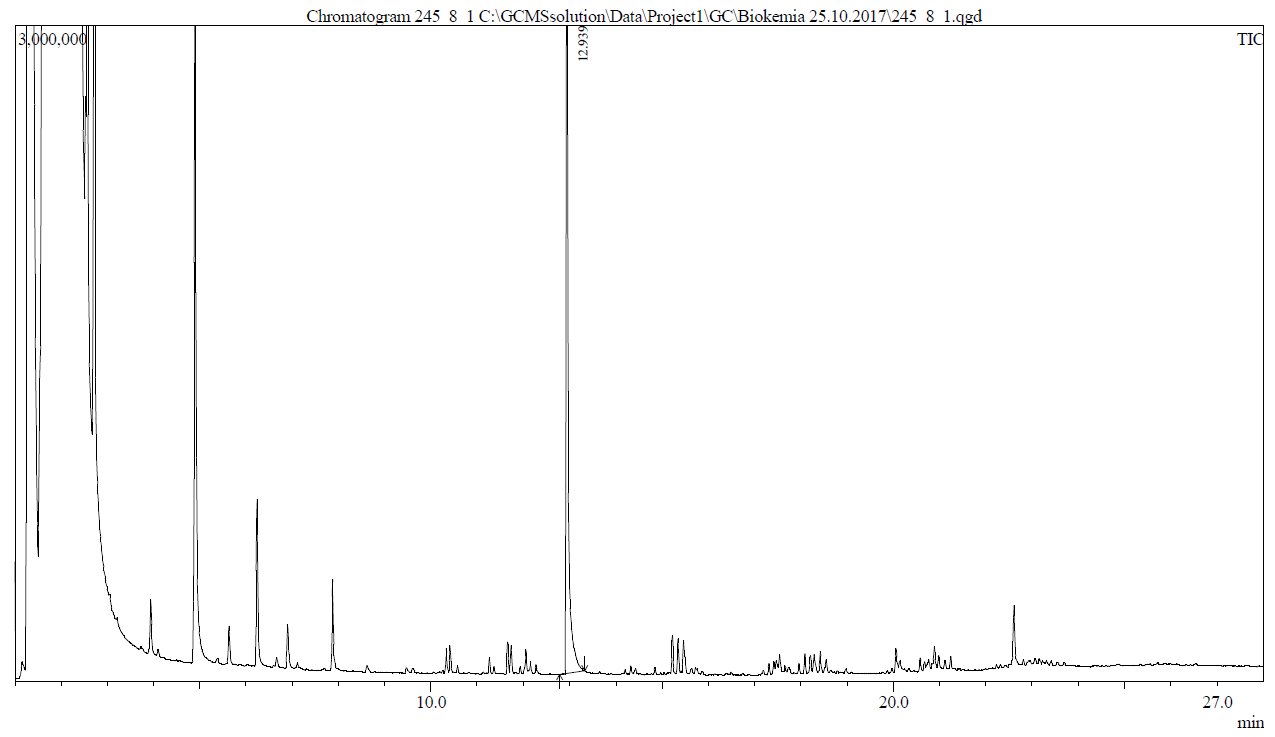


**Figure S39.** GC chromatogram of the styrene **2g** extracted with n-hexane


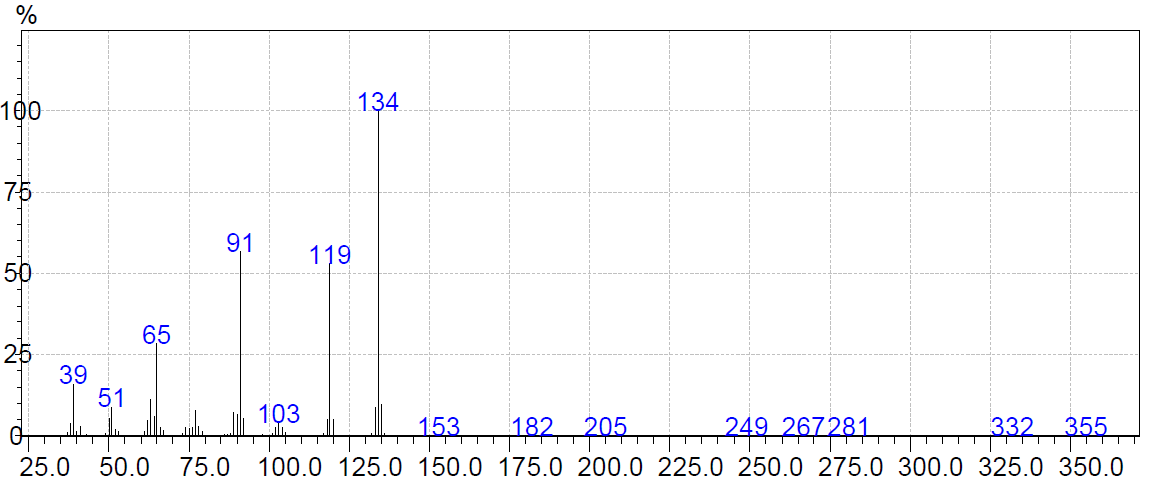

**Figure S40.** The MS spectrum for the product **2g**


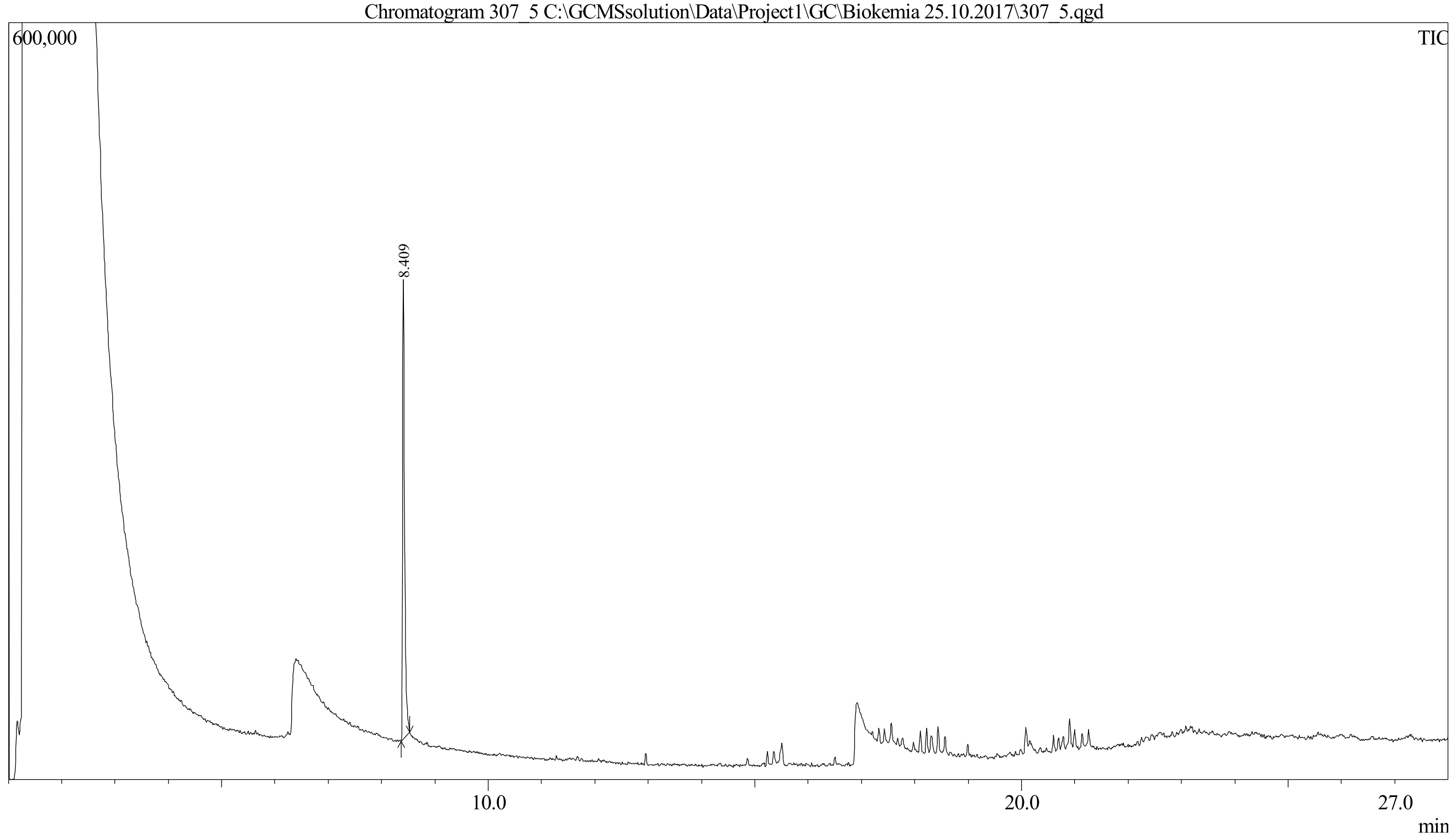


**Figure S41.** GC chromatogram of the styrene **2h** extracted with tert-butyl methyl ether


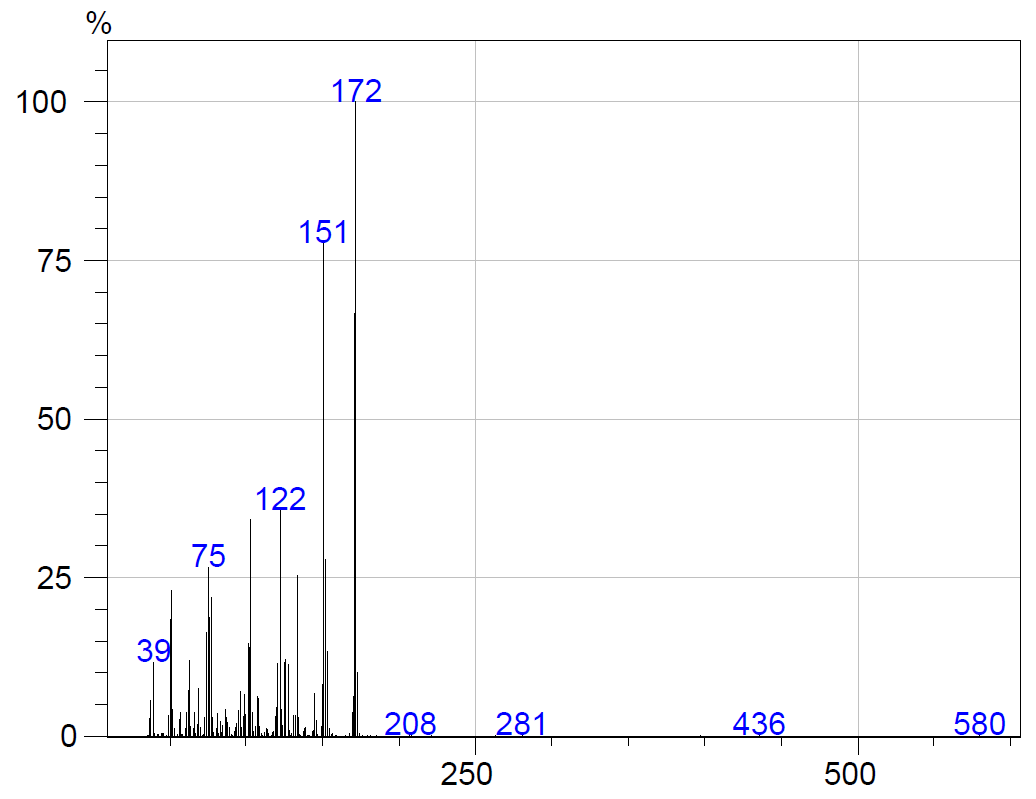

**Figure S42.** The MS spectrum for the product **2h**


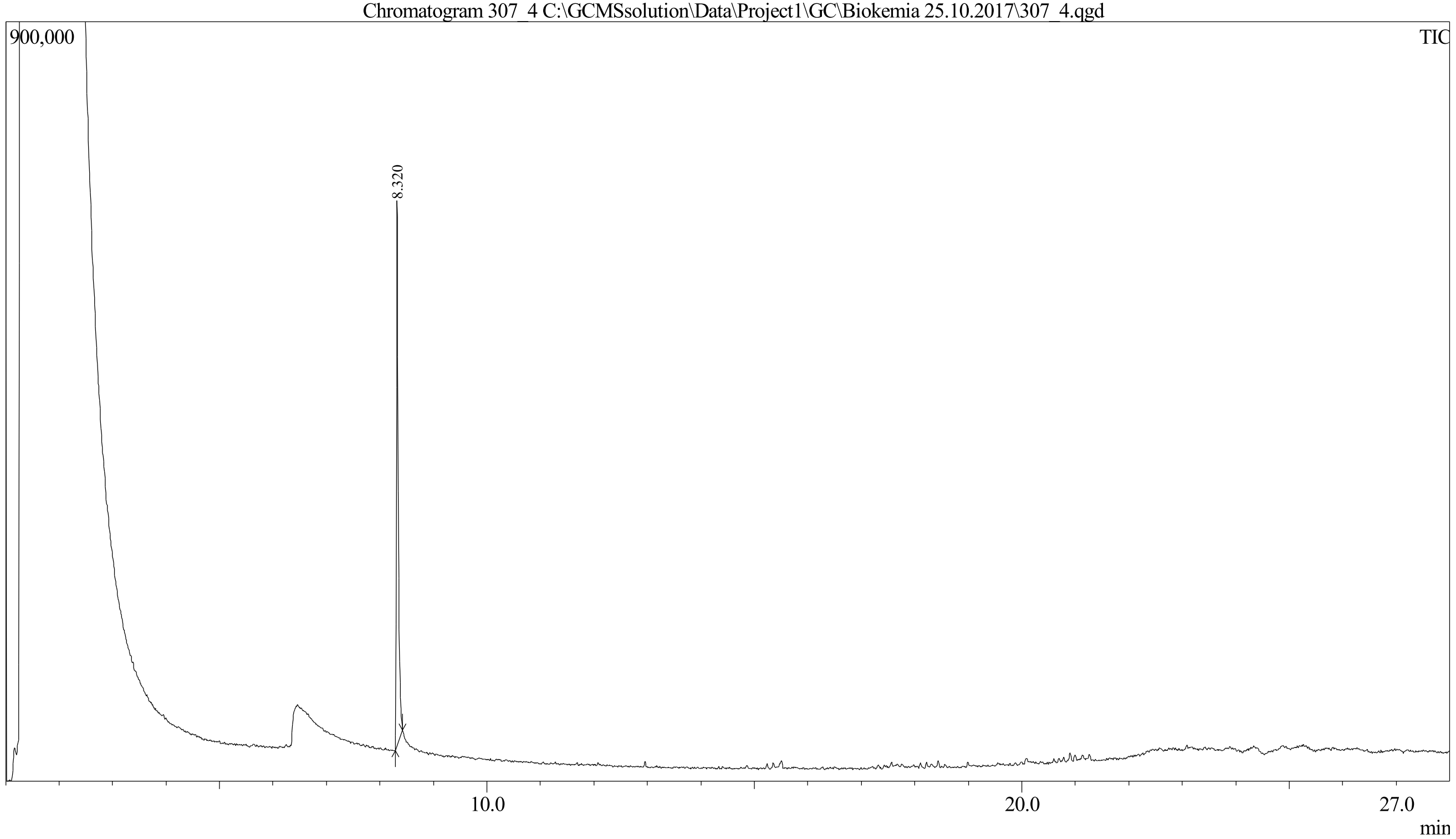


**Figure S43.** GC chromatogram of the styrene **2i** extracted with tert-butyl methyl ether


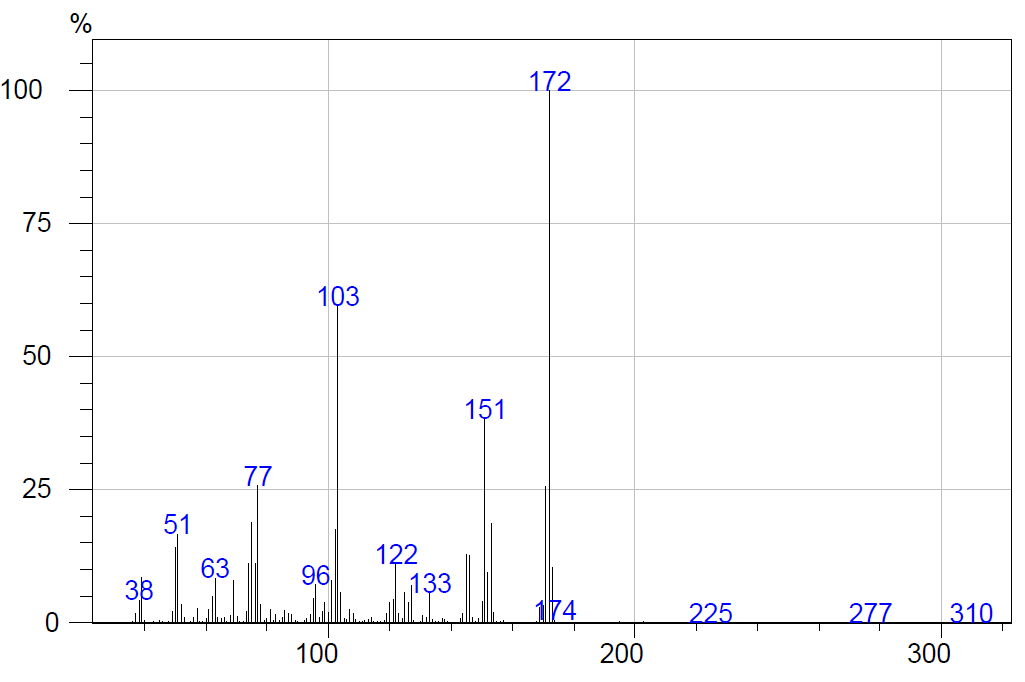

**Figure S44.** The MS spectrum for the product **2i**


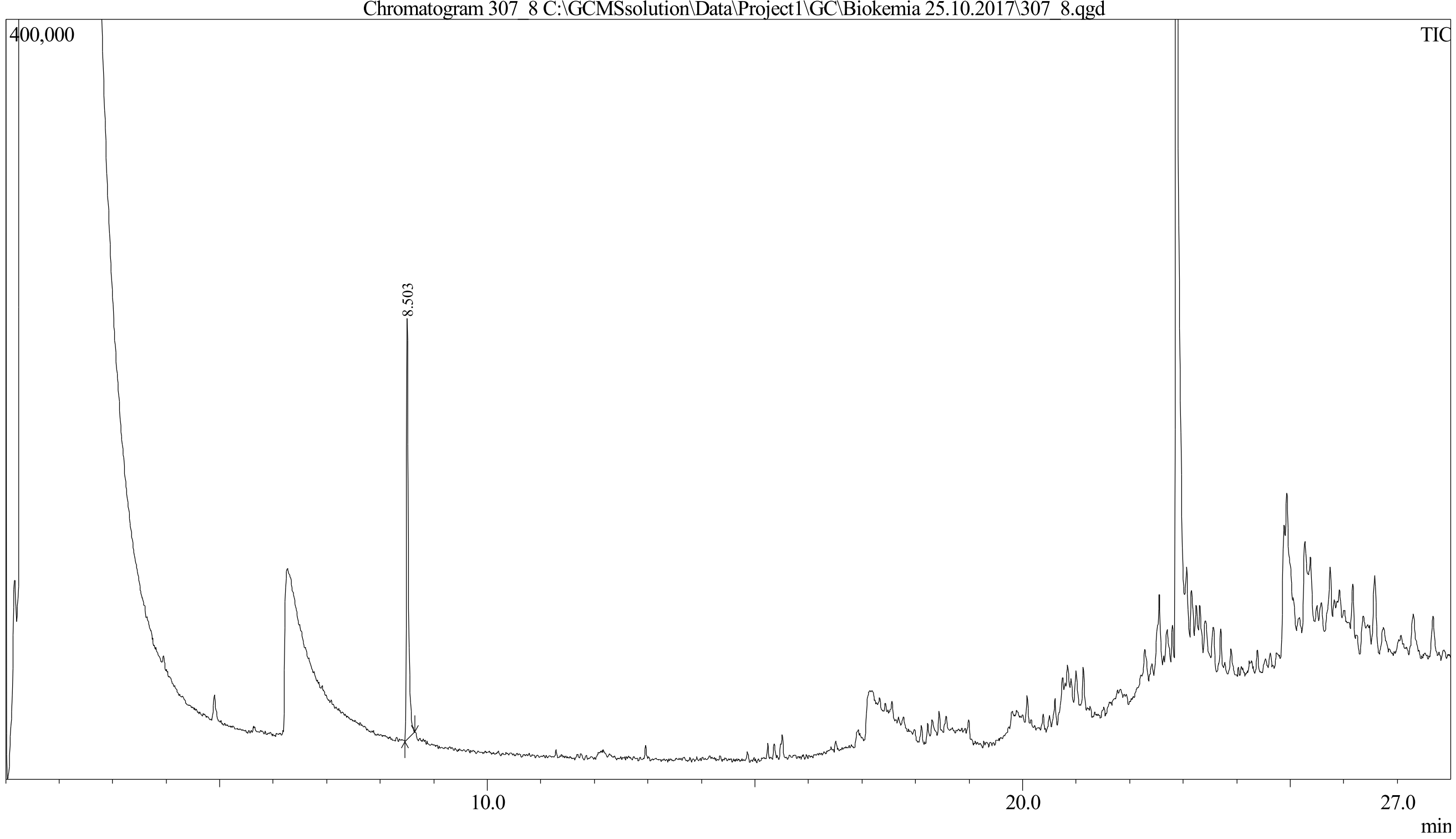


**Figure S45.** GC chromatogram of the styrene **2j** extracted with tert-butyl methyl ether


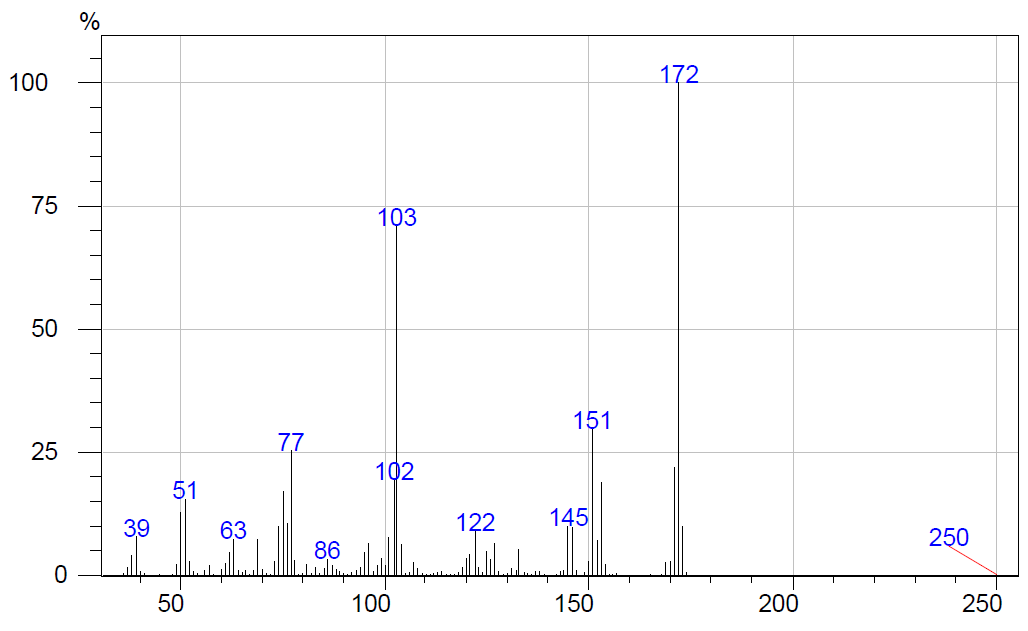

**Figure S46.** The MS spectrum for the product **2j**


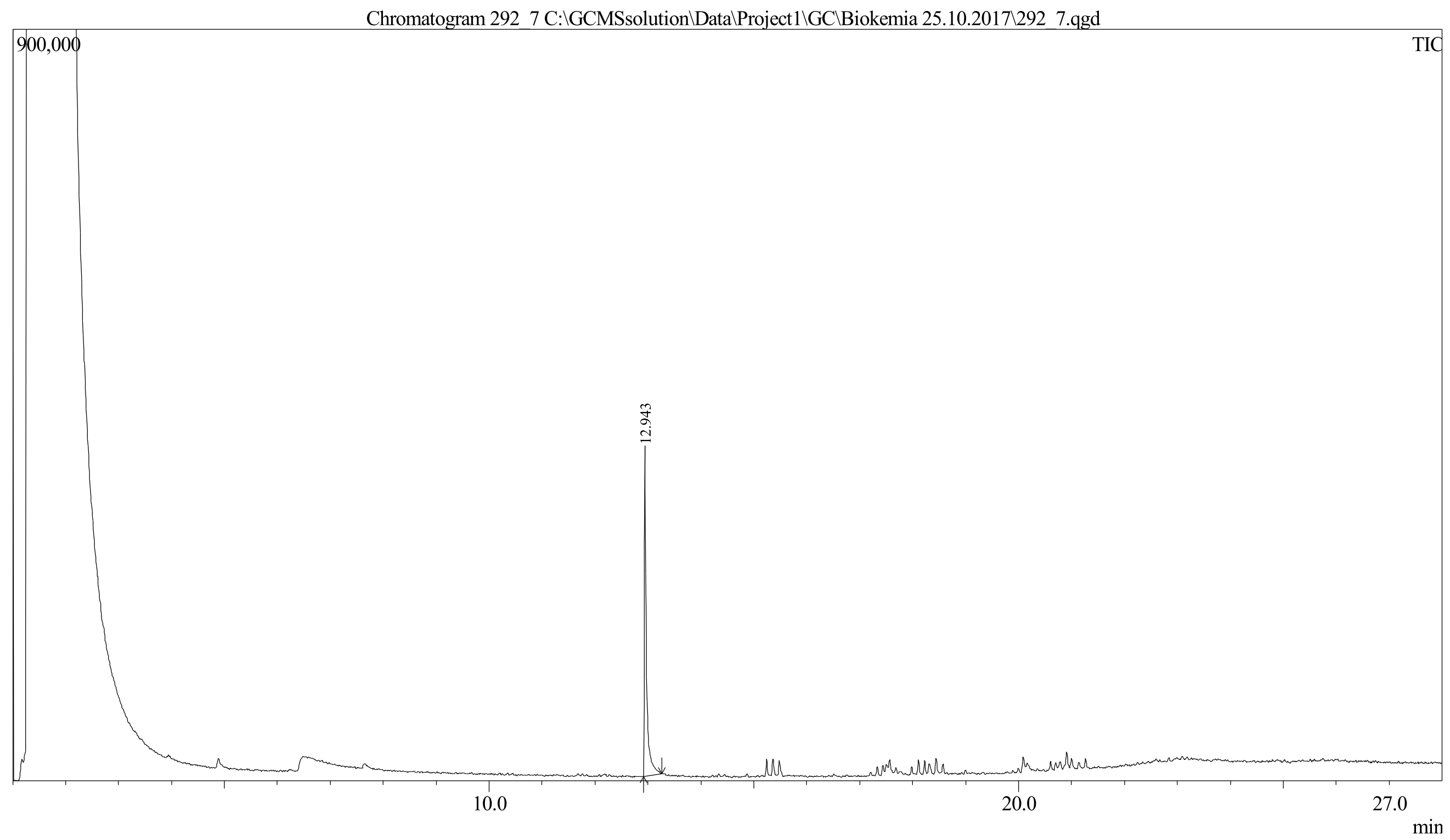


**Figure S47.** GC chromatogram of the styrene **2k** extracted with tert-butyl methyl ether


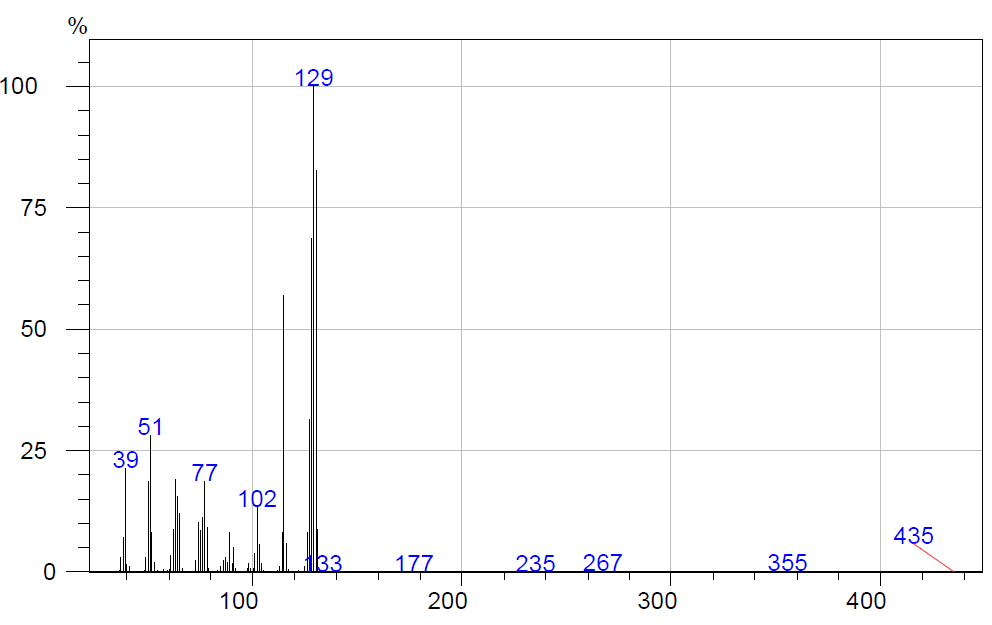

**Figure S48.** The MS spectrum for the product **2k**


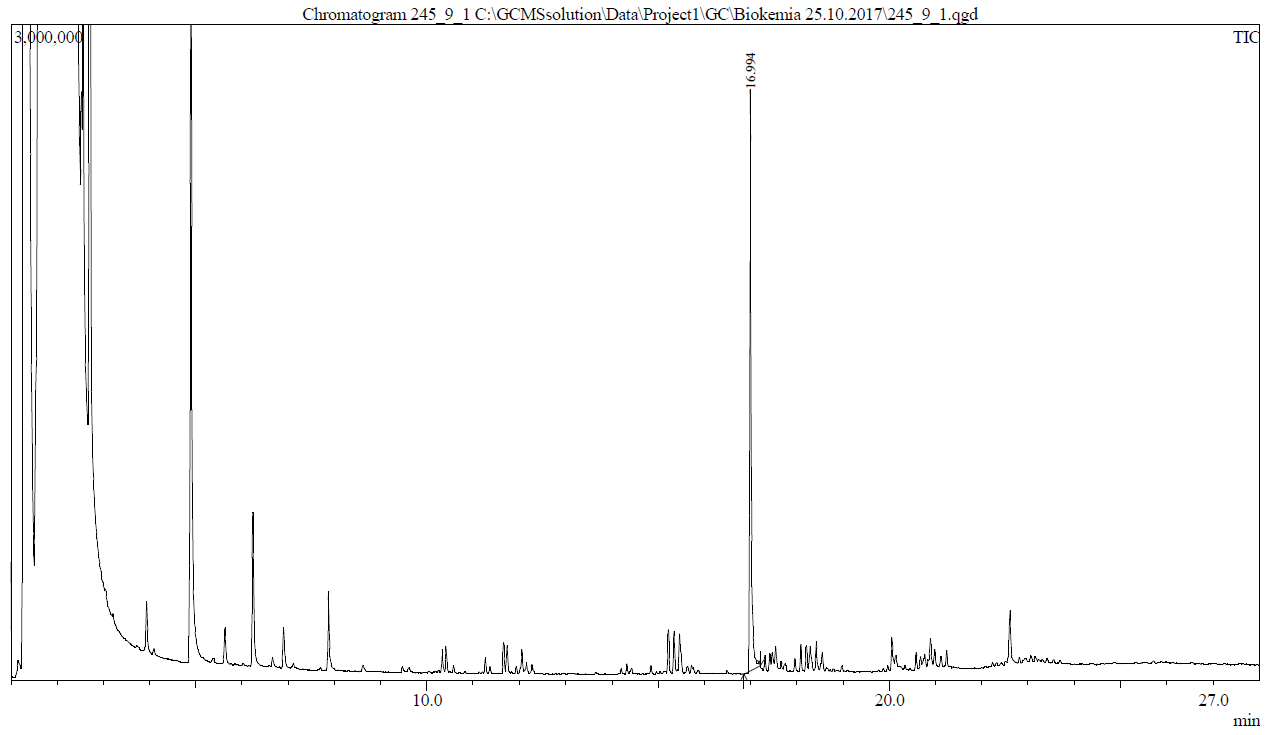


**Figure S49.** GC chromatogram of the styrene **2m** extracted with n-hexane


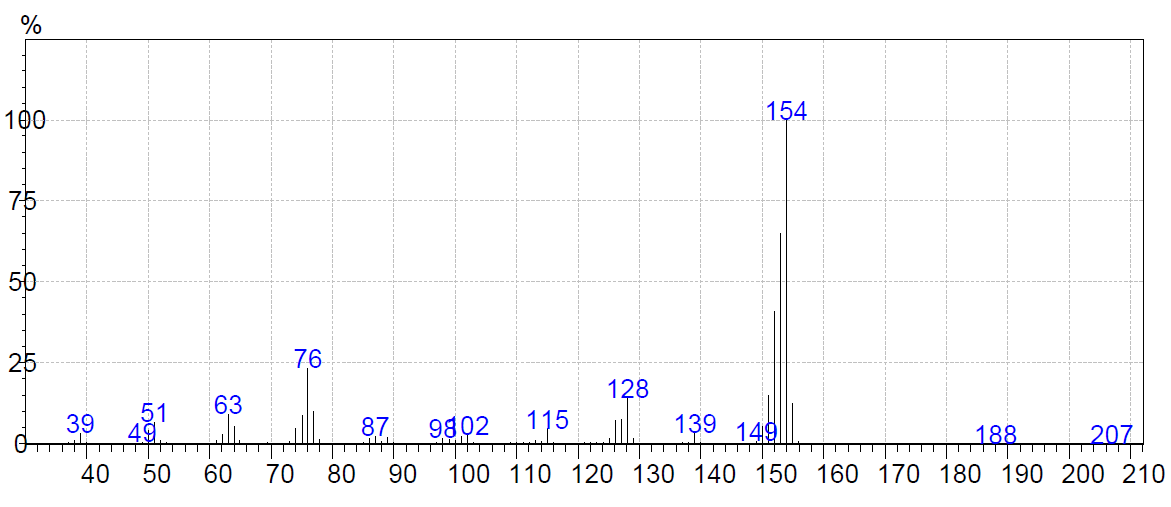

**Figure S50.** The MS spectrum for the product **2m**


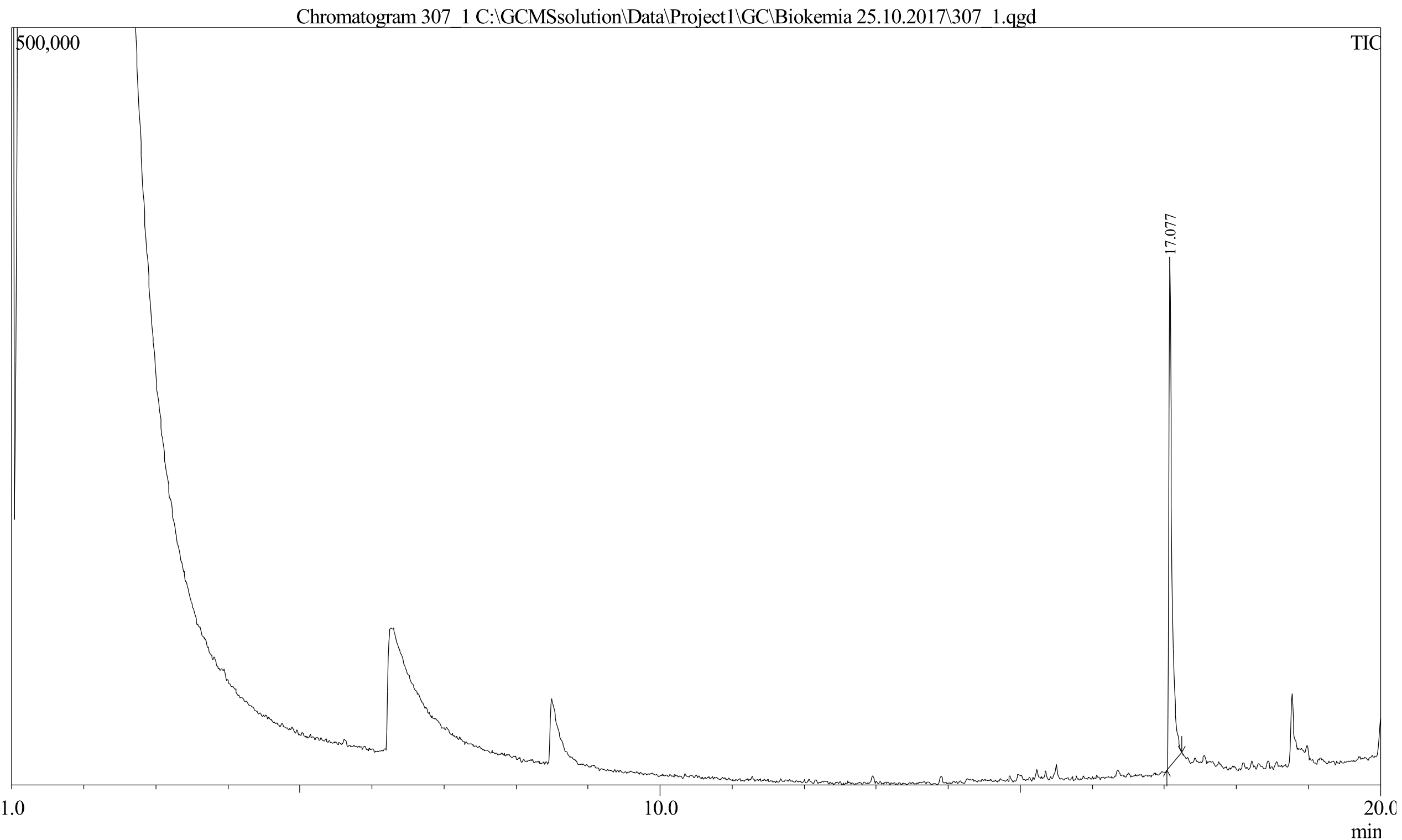


**Figure S51.** GC chromatogram of the styrene **2n** extracted with tert-butyl methyl ether


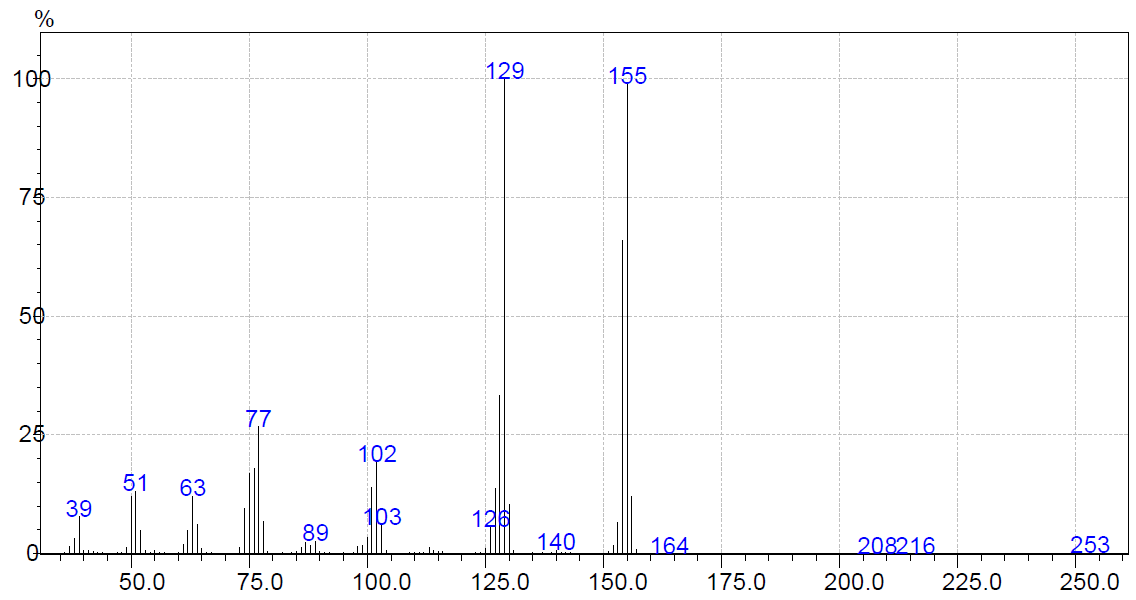

**Figure S52.** The MS spectrum for the product **2n**


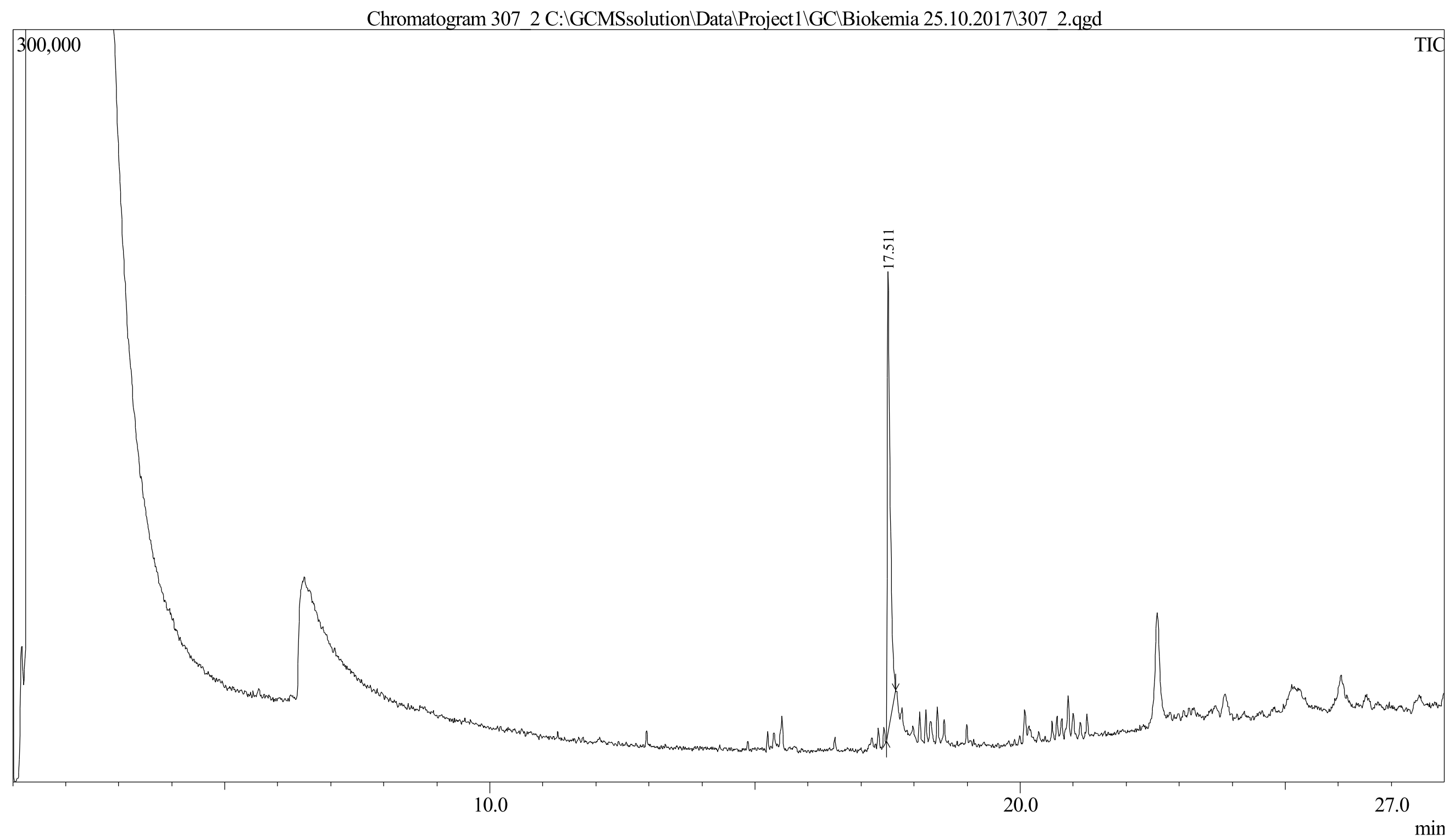


**Figure S53.** GC chromatogram of the styrene **2o** extracted with tert-butyl methyl ether


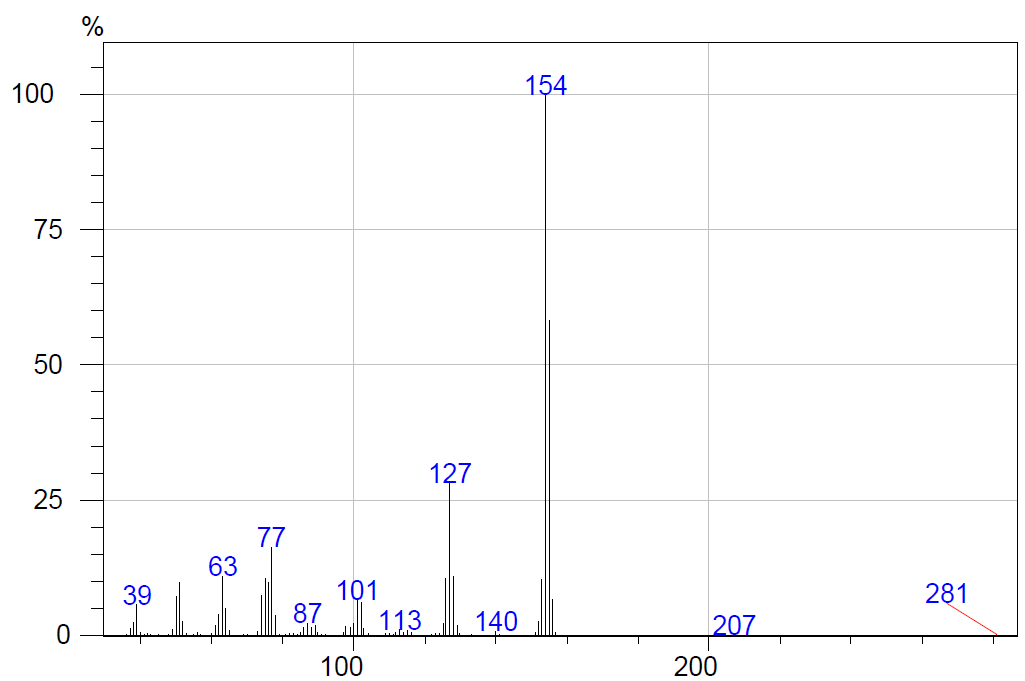

**Figure S54.** The MS spectrum for the product **2o**


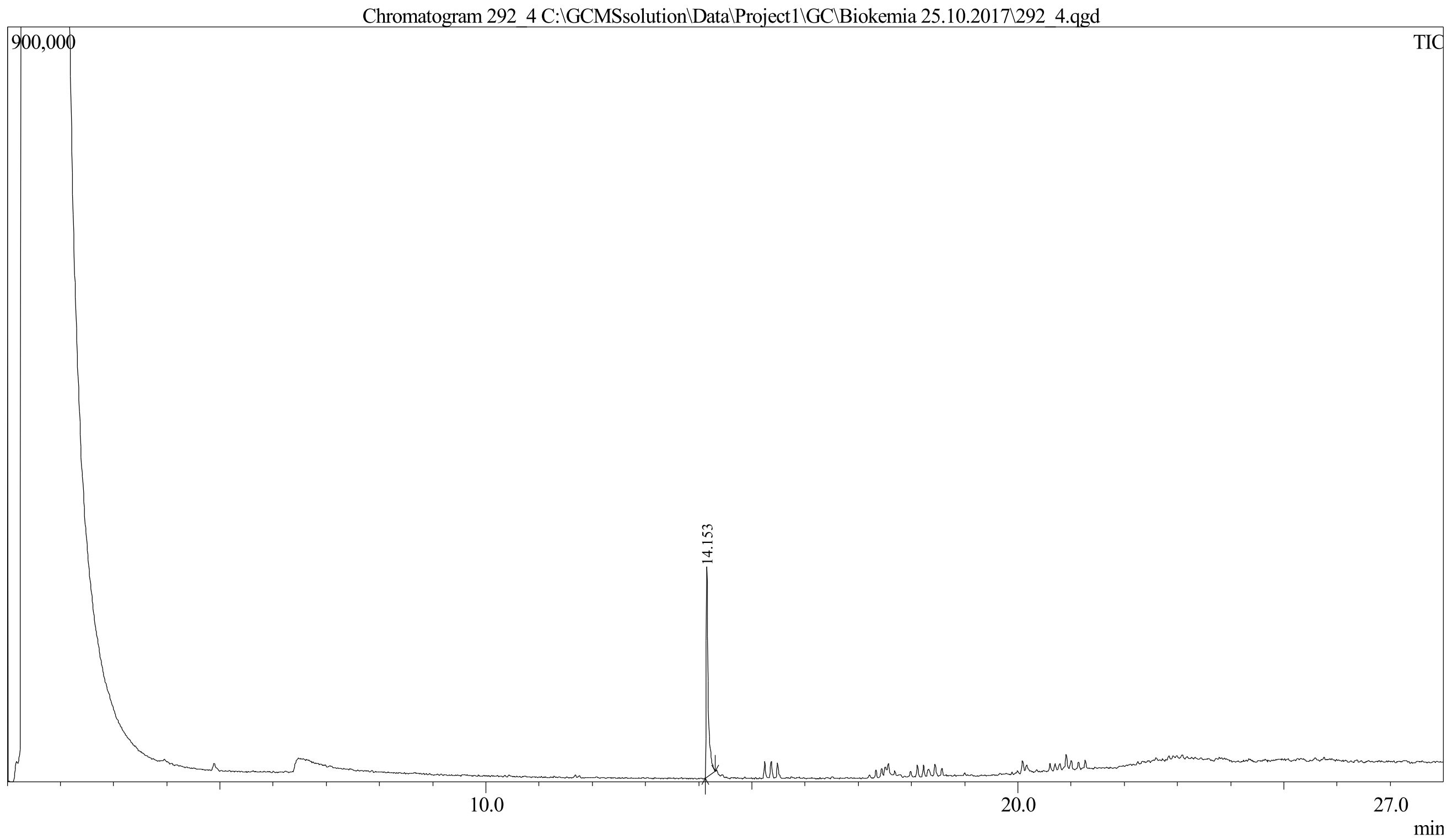


**Figure S55.** GC chromatogram of the styrene **2p** extracted with tert-butyl methyl ether


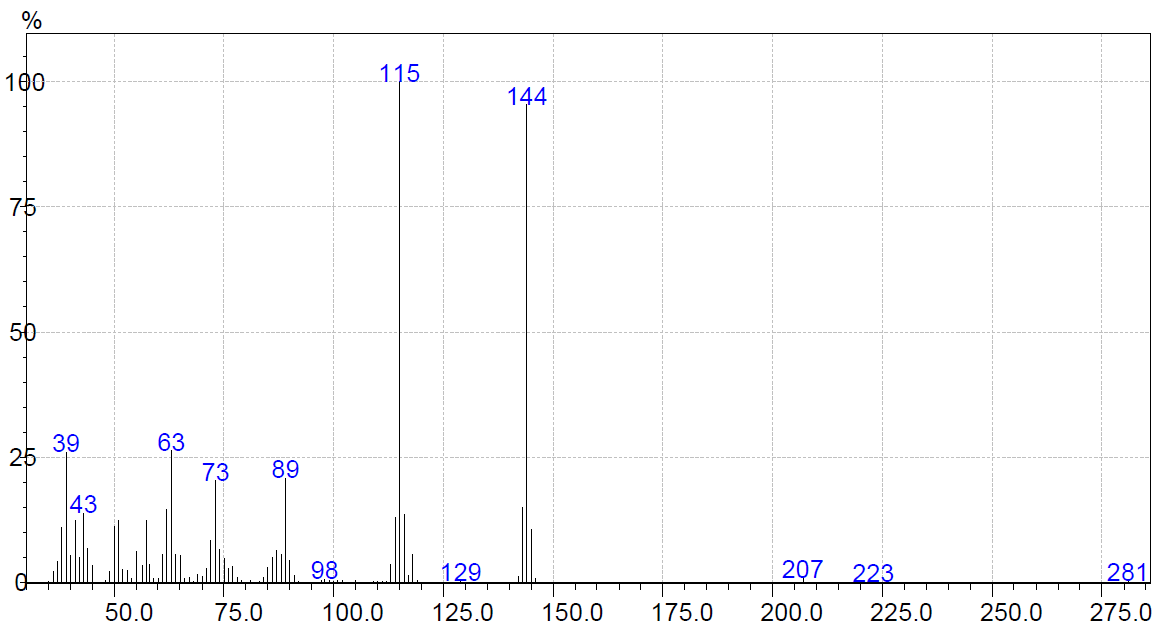

**Figure S56.** The MS spectrum for the product **2p**


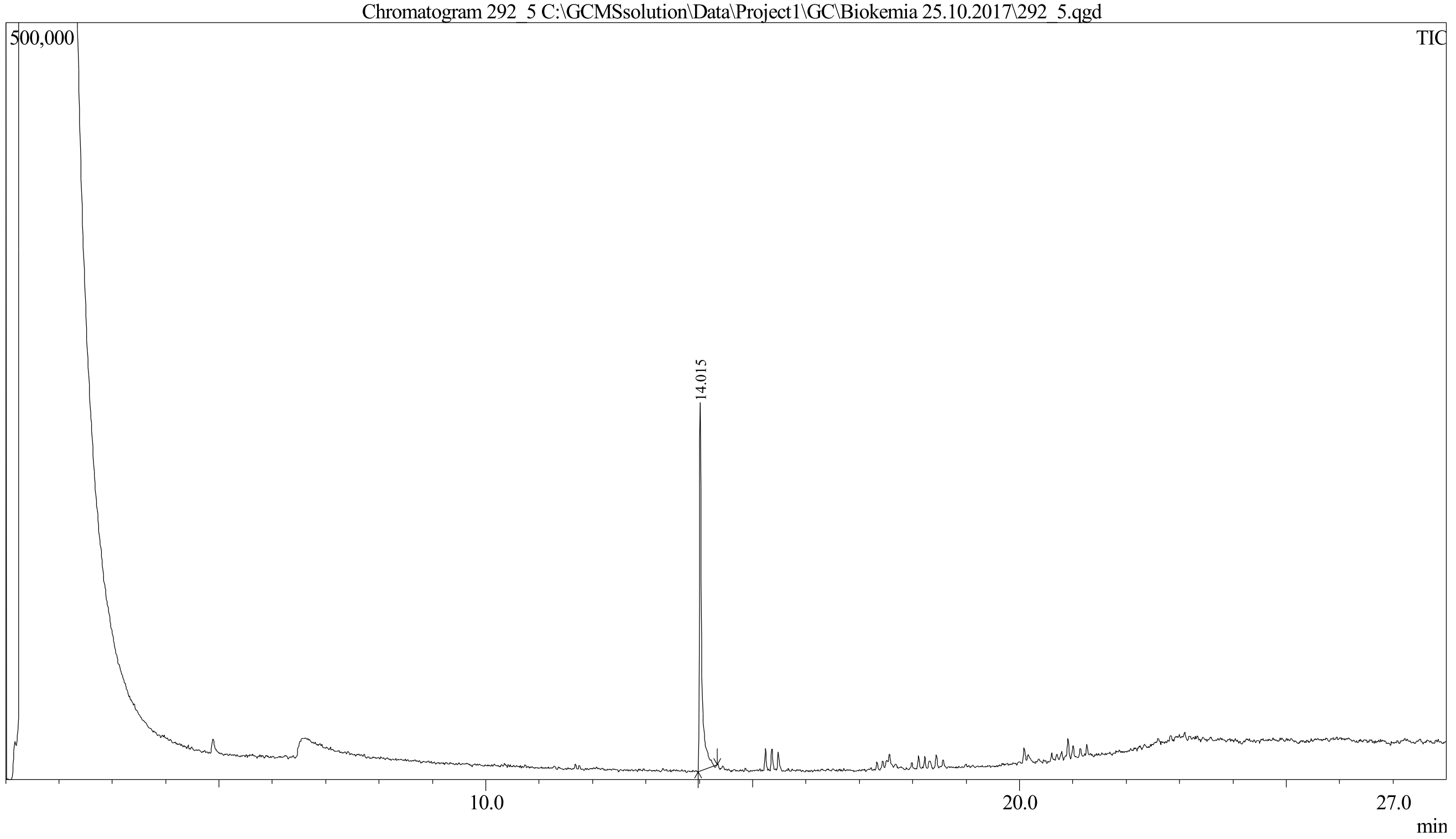


**Figure S57.** GC chromatogram of the styrene **2q** extracted with tert-butyl methyl ether


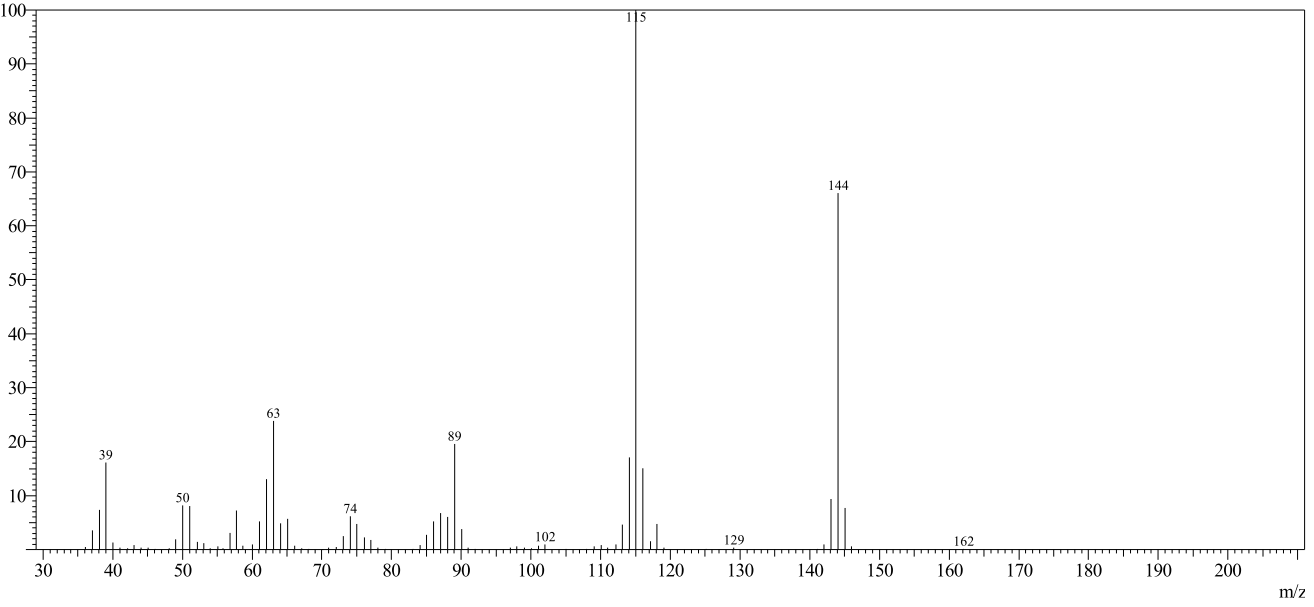

**Figure S58.** The MS spectrum for the product **2q**


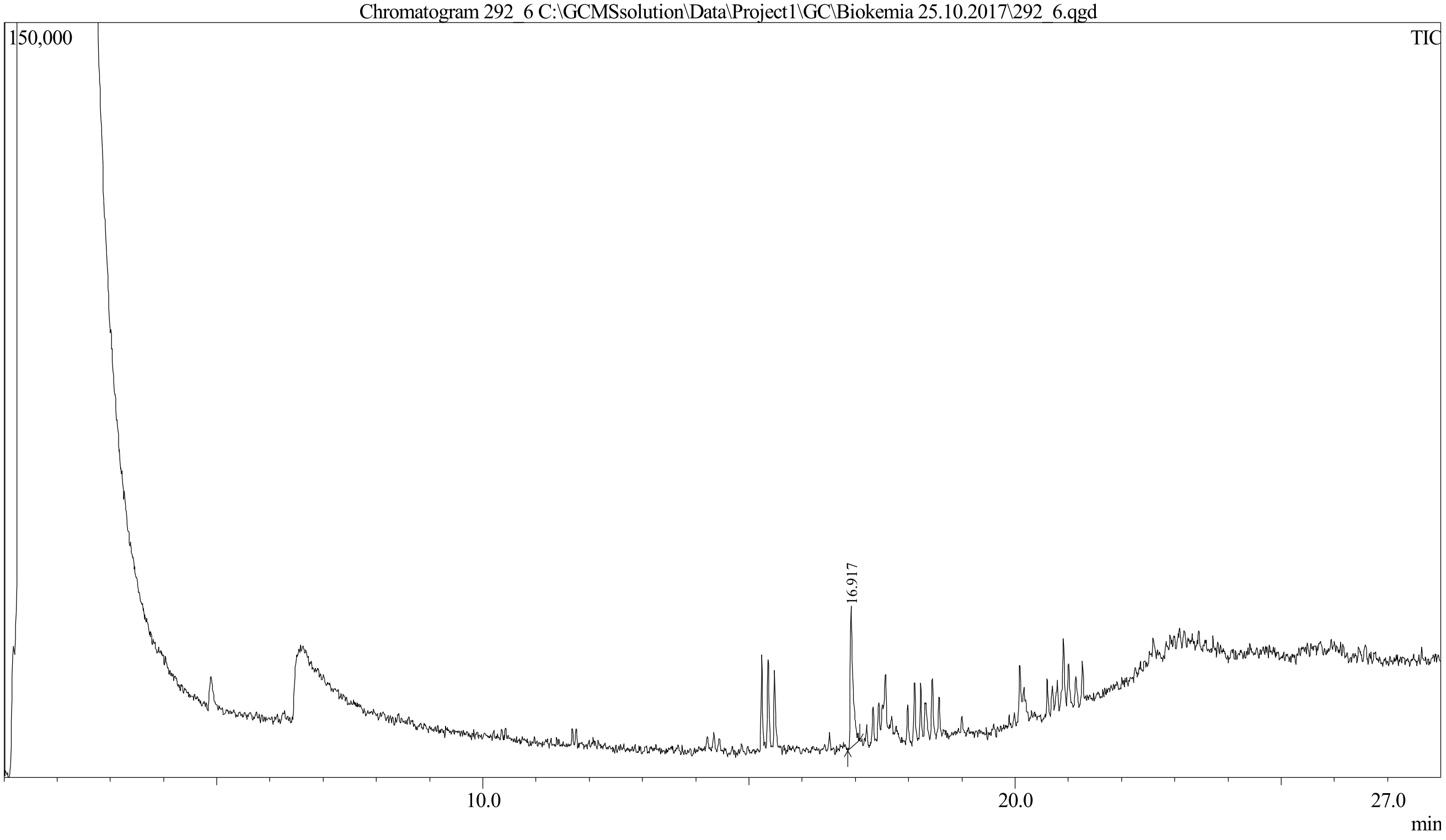


**Figure S59.** GC chromatogram of the styrene **2r** extracted with tert-butyl methyl ether


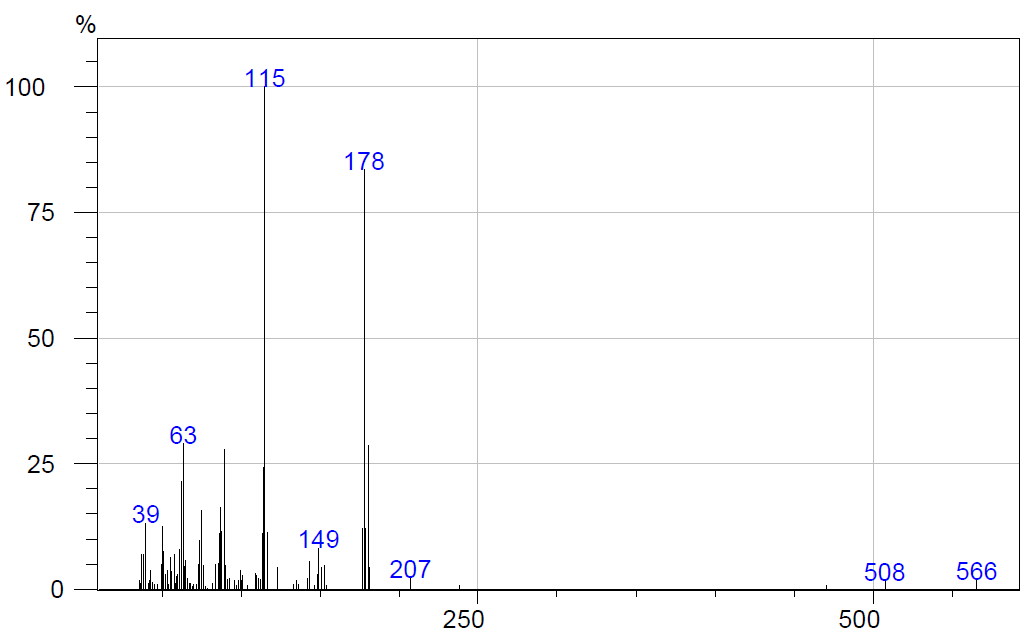

**Figure S60.** The MS spectrum for the product **2r**


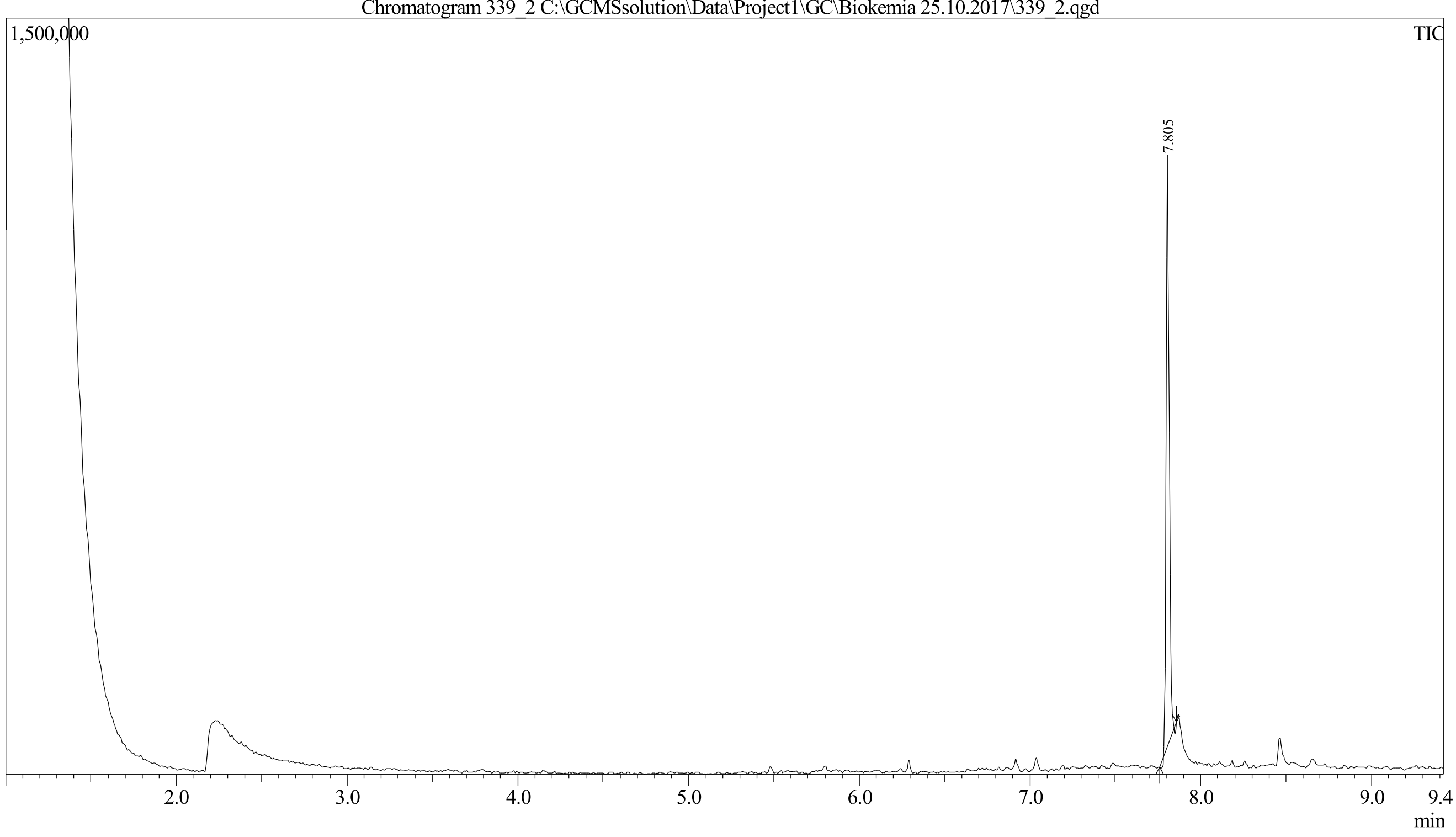


**Figure S61.** GC chromatogram of the styrene **2s** extracted with tert-butyl methyl ether


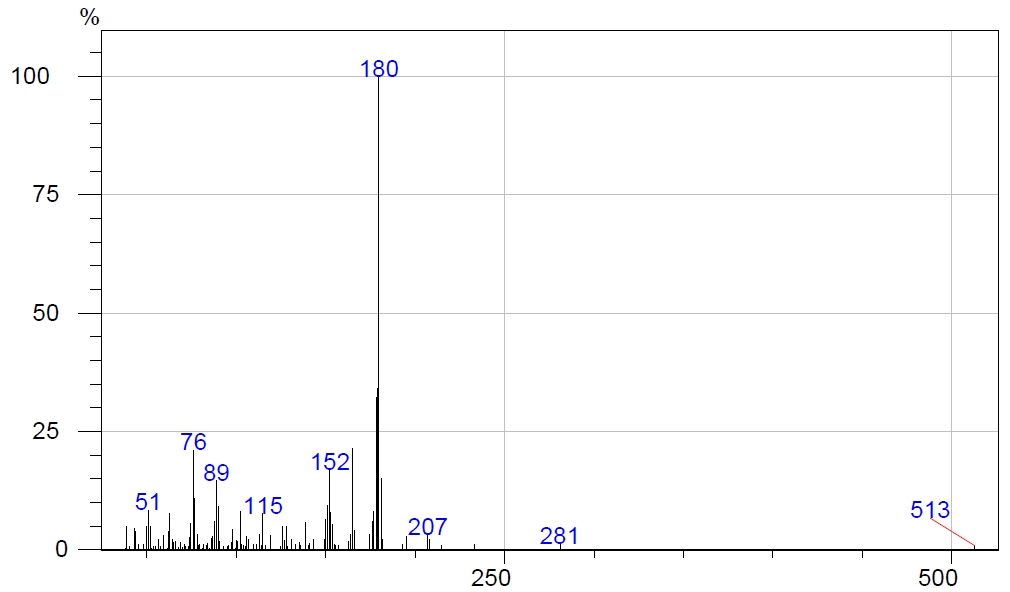

**Figure S62.** The MS spectrum for the product **2s**


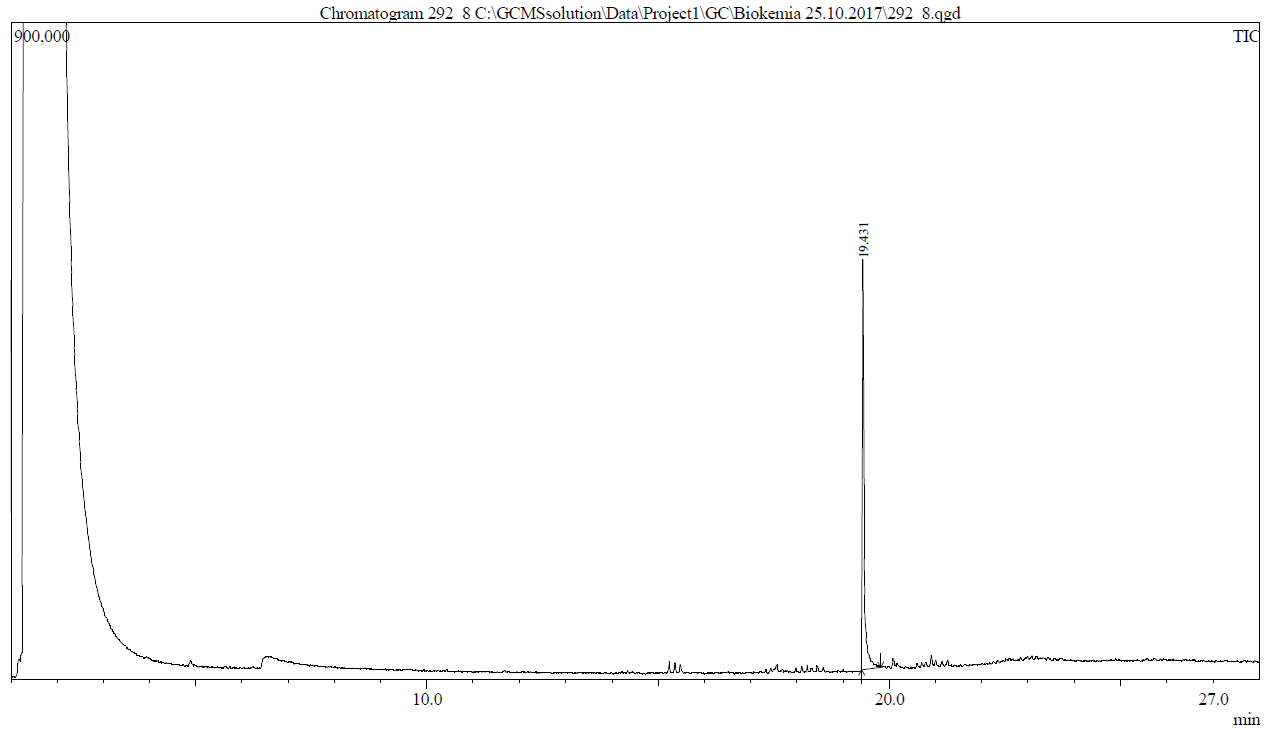


**Figure S63.** GC chromatogram of the styrene **2t** extracted with tert-butyl methyl ether


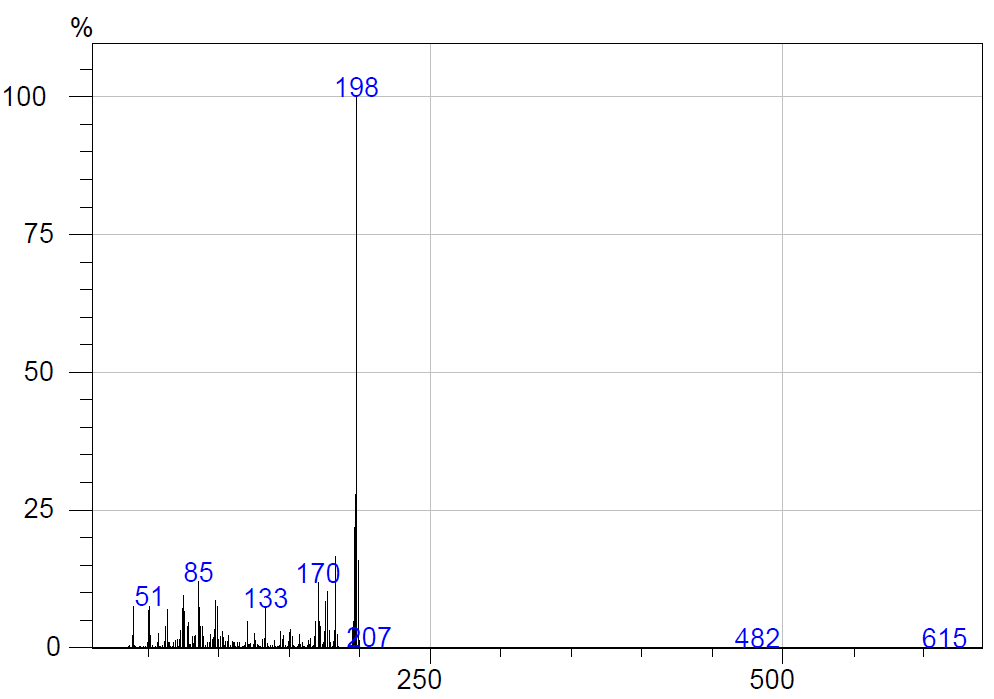

**Figure S64.** The MS spectrum for the product **2t**


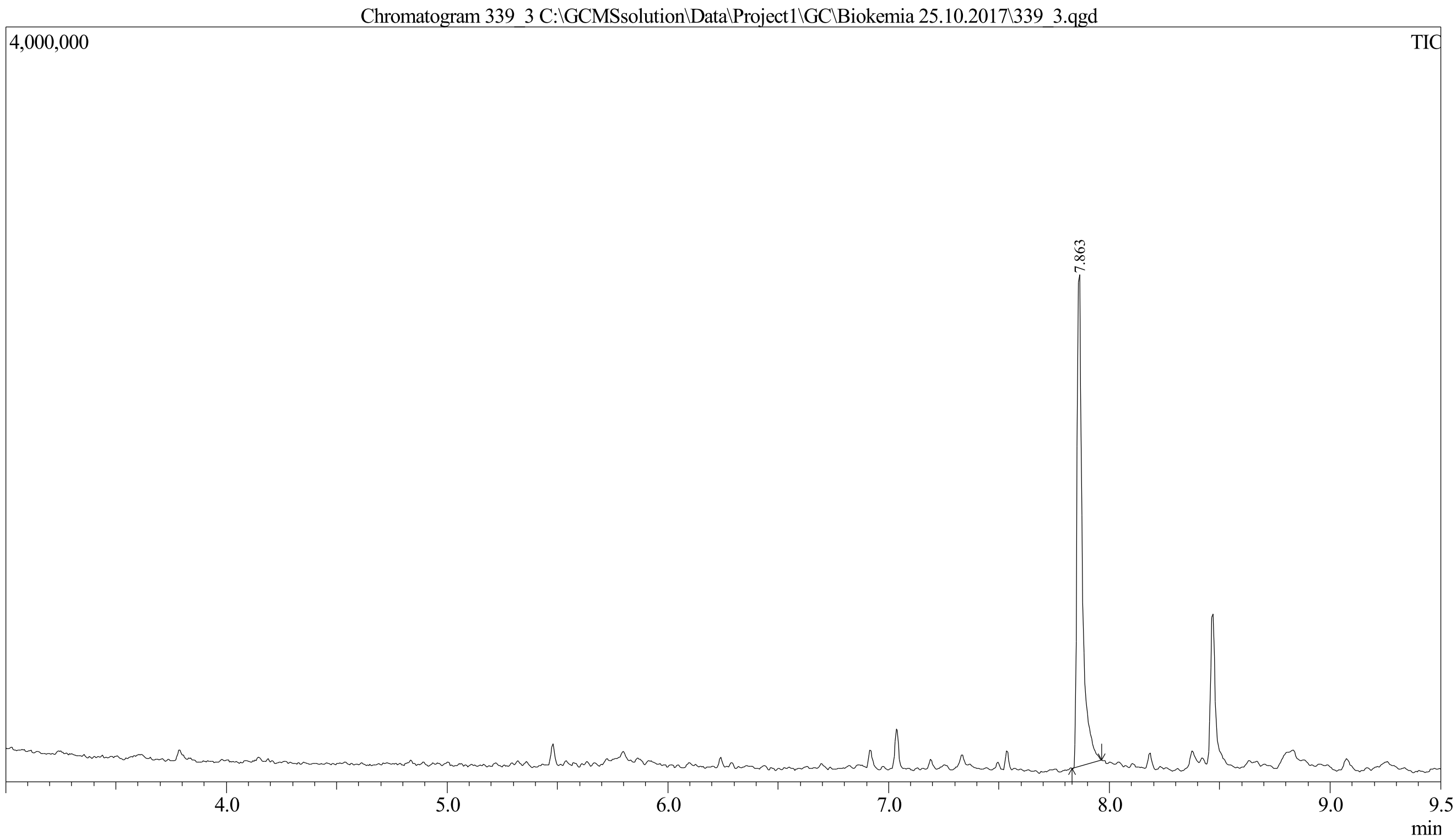


**Figure S65.** GC chromatogram of the styrene **2u** extracted with tert-butyl methyl ether


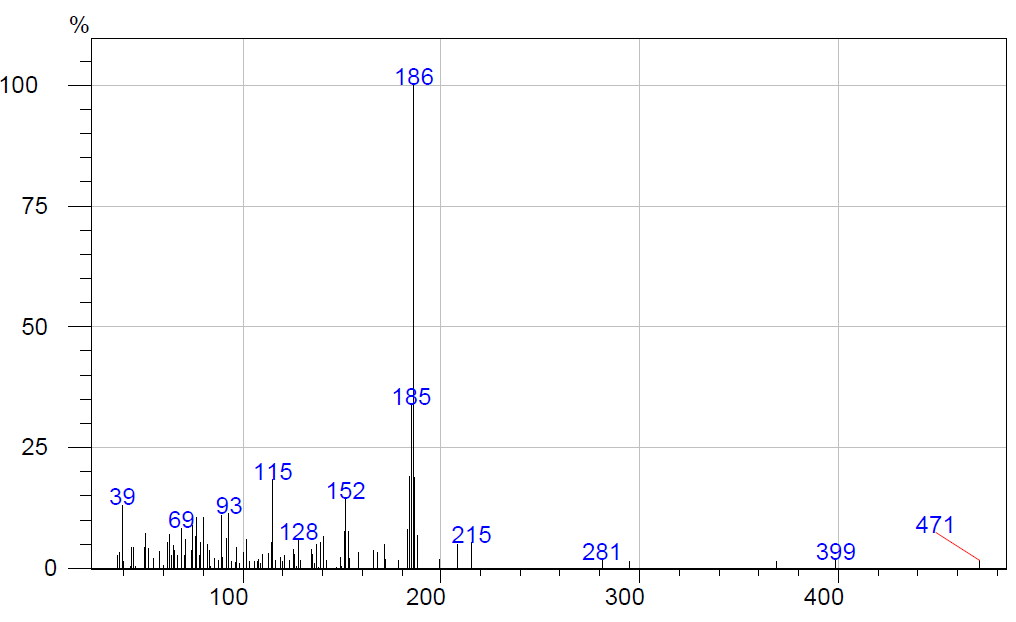

**Figure S66.** The MS spectrum for the product **2u**

**Figure S67.** GC chromatogram of the styrene **2w** extracted with tert-butyl methyl ether

**Figure S68.** The MS spectrum for the product **2w** obtained from the decarboxylation reaction of 1w with mutant variant I330A

# 3. Synthesis of acrylic acid derivatives 1a-x

**Figure S69.** Synthesis of the substrate library through the Knoevenagel-Doubner reaction.

*E)-3-phenylprop-2-enoic acid* (**1a**)

^1^H NMR (400 MHz, CD_3_OD) δ: 7.61 (d, *J* = 16.0 Hz, 1H), 7.56 – 7.49 (m, 2H), 7.33 (dd, *J* = 5.0, 1.9 Hz, 3H), 6.42 (d, *J* = 16.0 Hz, 1H); ^13^C NMR (101 MHz, CD_3_OD) δ: 170.31, 146.30, 135.80, 131.39, 129.99, 129.17, 119.32, 49.64, 49.43, 49.21, 49.00, 48.79, 48.57, 48.36.

*(E)-3-(2-bromophenyl)acrylic acid* (**1b**)

^1^H NMR (400 MHz, CD_3_OD) δ: 7.97 (d, *J* = 15.9 Hz, 1H), 7.69 (d, *J* = 7.8 Hz, 1H), 7.59 (d, *J* = 8.1 Hz, 1H), 7.33 (t, *J* = 7.1 Hz, 1H), 7.23 (t, *J* = 7.7 Hz, 1H), 6.41 (d, *J* = 15.9 Hz, 1H); ^13^C NMR (101 MHz, CD_3_OD) δ: 169.64, 144.15, 135.55, 134.46, 132.61, 129.15, 129.06, 126.04, 122.38, 49.64, 49.43, 49.21, 49.00, 48.79, 48.57, 48.36.

*(E)-3-(3-bromophenyl)acrylic acid* (**1c**)

^1^H NMR (400 MHz, CD_3_OD) δ: 7.65 (s, 1H), 7.61 – 7.31 (m, 3H), 7.21 (t, *J* = 7.8 Hz, 1H), 6.40 (d, *J* = 16.0 Hz, 1H); ^13^C NMR (101 MHz, CD_3_OD) δ: 169.81, 144.41, 138.15, 134.06, 131.85, 131.72, 127.84, 123.91, 121.10, 49.64, 49.43, 49.21, 49.00, 48.79, 48.57, 48.36.

*(E)-3-(4-bromophenyl)acrylic acid* (**1d**)

^1^H NMR (400 MHz, DMSO-*d*_6_) δ: 7.72 – 7.50 (m, 5H), 6.56 (d, *J* = 16.0 Hz, 1H); ^13^C NMR (101 MHz, DMSO-*d*_6_) δ: 167.49, 142.67, 133.57, 131.90, 130.21, 123.59, 120.17, 40.15, 39.94, 39.73, 39.52, 39.31, 39.10, 38.89.

*(E)-3-(2-methoxyphenyl)acrylic acid* (**1e**)

^1^H NMR (400 MHz, CD_3_OD) δ: 7.94 (d, *J* = 16.1 Hz, 1H), 7.53 (d, *J* = 7.8 Hz, 1H), 7.41 – 7.28 (m, 1H), 6.99 (d, *J* = 8.3 Hz, 1H), 6.93 (t, *J* = 7.5 Hz, 1H), 6.47 (d, *J* = 16.2 Hz, 1H), 3.86 (s, 3H); ^13^C NMR (101 MHz, CD_3_OD) δ: 170.92, 159.74, 141.62, 132.88, 129.66, 124.30, 121.80, 119.34, 112.39, 56.05, 49.64, 49.43, 49.21, 49.00, 48.79, 48.57, 48.36.

*(E)-3-(3-methoxyphenyl)acrylic acid* (**1f**)

^1^H NMR (400 MHz, CD_3_OD) δ: 7.53 (d, *J* = 16.0 Hz, 1H), 7.20 (t, *J* = 7.9 Hz, 1H), 7.11 – 6.96 (m, 2H), 6.86 (d, *J* = 8.3 Hz, 1H), 6.36 (d, *J* = 16.0 Hz, 1H), 3.71 (s, 3H); ^13^C NMR (101 MHz, CD_3_OD) δ: 170.28, 161.50, 146.25, 137.12, 130.95, 121.69, 119.56, 117.22, 114.00, 55.75, 49.64, 49.43, 49.21, 49.00, 48.79, 48.57, 48.36.

*(E)-3-(4-methoxyphenyl)acrylic acid* (**1g**)

^1^H NMR (600 MHz, CDCl_3_) δ: 7.75 (d, *J* = 14.9 Hz, 1H), 7.51 (d, *J* = 8.2 Hz, 1H), 7.26 (s, 2H), 6.92 (d, *J* = 8.3 Hz, 1H), 6.32 (d, *J* = 15.0 Hz, 1H), 3.85 (s, 3H); ^13^C NMR (151 MHz, CDCl_3_) δ: 172.05, 161.69, 146.61, 130.03, 126.77, 114.56, 114.35, 77.16, 76.95, 76.74, 55.35.

*(E)-3-(2-(trifluoromethyl)phenyl)acrylic acid* (**1h**)

^1^H NMR (600 MHz, CD_3_OD) δ: 7.93 (d, *J* = 15.7 Hz, 1H), 7.80 (d, *J* = 7.8 Hz, 1H), 7.66 (d, *J* = 7.8 Hz, 1H), 7.57 (t, *J* = 7.7 Hz, 1H), 7.47 (t, *J* = 7.7 Hz, 1H), 6.42 (d, *J* = 15.7 Hz, 1H); ^13^C NMR (151 MHz, CD_3_OD) δ: 169.29, 141.12, 141.10, 134.45, 133.72, 131.03, 129.27, 127.13, 127.09, 123.95, 49.43, 49.28, 49.14, 49.00, 48.86, 48.72, 48.57.

*(E)-3-(3-(trifluoromethyl)phenyl)acrylic acid* (**1i**)

^1^H NMR (600 MHz, CD_3_OD) δ: 7.78 (d, *J* = 6.2 Hz, 2H), 7.66 – 7.58 (m, 2H), 7.52 (t, *J* = 7.9 Hz, 1H), 6.51 (d, *J* = 16.1 Hz, 1H); ^13^C NMR (151 MHz, CD_3_OD) δ: 169.71, 144.25, 136.96, 132.41, 130.89, 127.60, 127.58, 125.79, 125.76, 121.68, 49.43, 49.28, 49.14, 49.00, 48.86, 48.72, 48.58.

*(E)-3-(4-(trifluoromethyl)phenyl)acrylic acid* (**1j**)

^1^H NMR (600 MHz, CD_3_OD) δ: 7.81 – 7.56 (m, 5H), 6.56 (d, *J* = 16.0 Hz, 1H); ^13^C NMR (151 MHz, CD_3_OD) δ: 168.23, 142.77, 138.25, 128.23, 125.44, 125.42, 120.98, 48.02, 47.88, 47.74, 47.60, 47.46, 47.31, 47.17.

*(2E,4E)-5-phenylpenta-2,4-dienoic acid* (**1k**)

^1^H NMR (400 MHz, CDCl_3_) δ: 7.55 (dd, *J* = 15.3, 9.7 Hz, 1H), 7.49 (d, *J* = 7.0 Hz, 2H), 7.35 (ddd, *J* = 10.7, 9.9, 5.3 Hz, 3H), 7.01–6.84 (m, 2H), 6.01 (d, *J* = 15.2 Hz, 1H); ^13^C NMR (100 MHz, CDCl_3_) δ: 172.55, 147.13, 141.80, 135.95, 129.47, 129.00, 127.50, 126.09, 120.41.

*(E)-5-phenylpent-2-enoic acid* (**1l**)

^1^H NMR (600 MHz, CD_3_OD) δ: 7.17 (t, *J* = 7.6 Hz, 2H), 7.11 – 7.05 (m, 3H), 6.87 (dt, *J* = 15.6, 6.9 Hz, 1H), 5.69 (d, *J* = 15.6 Hz, 1H), 2.67 (t, *J* = 7.6 Hz, 2H), 2.42 (q, *J* = 6.9 Hz, 2H); ^13^C NMR (151 MHz, CD_3_OD) δ: 169.96, 150.12, 142.21, 129.43, 127.10, 122.95, 49.43, 49.28, 49.14, 49.00, 48.86, 48.72, 48.57, 35.38, 35.00.

*(E)-3-(naphthalen-2-yl)acrylic acid* (**1m**)

^1^H NMR (600 MHz, DMSO-*d*_6_) δ: 8.02–7.76 (m, 4H), 7.71 (d, *J* = 8.3 Hz, 1H), 7.48 (t, *J* = 6.9 Hz, 2H), 7.27 (d, *J* = 15.8 Hz, 1H), 6.53 (d, *J* = 15.9 Hz, 1H); ^13^C NMR (151 MHz, D DMSO-*d*_6_) δ: 170.49, 135.35, 134.43, 133.26, 132.77, 130.91, 128.13, 127.98, 127.57, 126.92, 126.37, 126.07, 124.04.

*(E)-3-(quinolin-2-yl)acrylic acid* (**1n**)

^1^H NMR (400 MHz, DMSO-*d*_6_) δ: 9.41 – 7.25 (m, 7H), 7.02 (d, *J* = 16.0 Hz, 1H); ^13^C NMR (101 MHz, DMSO) δ: 167.23, 153.12, 147.49, 143.50, 137.09, 130.24, 129.20, 127.90, 127.79, 127.40, 124.60, 120.87, 40.15, 39.94, 39.73, 39.52, 39.31, 39.10, 38.89.

*(E)-3-(quinolin-4-yl)acrylic acid* (**1o**)

^1^H NMR (600 MHz, DMSO-d_6_) δ: 8.93 (d, *J* = 4.5 Hz, 1H), 8.32 (d, *J* = 15.8 Hz, 1H), 8.25 (d, *J* = 8.5 Hz, 1H), 8.08 (d, *J* = 8.4 Hz, 1H), 7.85 (d, *J* = 4.5 Hz, 1H), 7.82 (t, *J* = 7.6 Hz, 1H), 7.69 (t, J = 7.7 Hz, 1H), 6.80 (d, *J* = 15.8 Hz, 1H); ^13^C NMR (151 MHz, DMSO) δ: 166.95, 150.41, 148.15, 139.23, 137.85, 129.85, 129.72, 127.49, 126.00, 125.43, 123.55, 118.62, 39.94, 39.80, 39.66, 39.52, 39.38, 39.24, 39.10.

*(E)-3-(benzofuran-2-yl)acrylic acid* (**1p**)

^1^H NMR (600 MHz, CD_3_OD) δ: 7.53 – 7.42 (m, 2H), 7.38 (d, *J* = 8.4 Hz, 1H), 7.26 (t, *J* = 7.8 Hz, 1H), 7.13 (t, *J* = 7.5 Hz, 1H), 6.98 (s, 1H), 6.38 (d, *J* = 15.6 Hz, 1H); ^13^C NMR (151 MHz, CD_3_OD) δ: 169.78, 156.97, 153.63, 132.87, 129.76, 127.67, 124.50, 122.94, 119.85, 112.43, 112.19, 49.43, 49.28, 49.25, 49.14, 49.00, 48.92, 48.86, 48.72, 48.68, 48.57.

*(E)-3-(benzofuran-3-yl)acrylic acid* (**1q**)

^1^H NMR (600 MHz, CD_3_OD) δ: 8.02 (s, 1H), 7.75 (d, *J* = 7.5 Hz, 1H), 7.70 (d, *J* = 16.1 Hz, 1H), 7.42 (d, *J* = 8.0 Hz, 1H), 7.25 (dd, *J* = 11.8, 7.7 Hz, 2H), 6.47 (d, *J* = 16.1 Hz, 1H); ^13^C NMR (151 MHz, CD_3_OD) δ: 169.22, 157.55, 150.24, 136.18, 126.53, 125.81, 124.98, 121.95, 118.94, 118.55, 112.81, 49.43, 49.28, 49.14, 49.00, 48.86, 48.72, 48.57.

*(E)-3-(5-chlorobenzofuran-2-yl)acrylic acid* (**1r**)

^1^H NMR (400 MHz, DMSO-*d*_6_) δ: 7.78 (s, 1H), 7.64 (d, *J* = 8.8 Hz, 1H), 7.55 (d, *J* = 15.8 Hz, 1H), 7.41 (d, *J* = 8.8 Hz, 1H), 7.30 (s, 1H), 6.45 (d, *J* = 15.7 Hz, 1H); ^13^C NMR (101 MHz, DMSO) δ: 167.04, 153.75, 153.33, 130.33, 129.72, 127.87, 126.35, 121.42, 121.15, 112.96, 110.69, 40.15, 39.94, 39.73, 39.52, 39.31, 39.10, 38.89.

*(E)-3-([1,1'-biphenyl]-4-yl)acrylic acid* (**1s**)

^1^H NMR (400 MHz, DMSO-*d*_6_) δ: 7.81 (d, *J* = 8.1 Hz, 2H), 7.72 (m, 4H), 7.61 (d, *J* = 16 Hz, 1H), 7.47 (t, *J* = 8.1 Hz, 2H), 7.42 (t, J = 8.1 Hz, 1H), 6.65 (d, *J* = 16 Hz, 1H); ^13^C NMR (101 MHz, DMSO-*d*_6_) δ: 167.39, 143.49, 142.06, 139.81, 133.67, 129.57, 129.01, 128.15, 127.29, 126.71, 119.31

*(E)-3-(4'-fluoro-[1,1'-biphenyl]-4-yl)acrylic acid* (**1t**)

^1^H NMR (600 MHz, DMSO-*d*_6_) δ: 7.79 – 7.71 (m, 4H), 7.68 (d, *J* = 8.2 Hz, 2H), 7.61 (d, *J* = 16.0 Hz, 1H), 7.28 (t, *J* = 8.8 Hz, 2H), 6.55 (d, *J* = 16.0 Hz, 1H); ^13^C NMR (151 MHz, DMSO-*d*_6_) δ: 167.68, 163.05, 161.42, 143.47, 140.74, 135.83, 135.81, 133.44, 128.04 (d, ^1^*J*_13C-19F_ = 277 Hz), 128.85 (d, ^3^*J*_13C -19F_ = 7.55 Hz), , 119.31, 115.93 (d, ^2^*J*_13C-19F_ = 21.14 Hz)

*(E)-3-(5-phenylthiophen-2-yl)acrylic acid* (**1u**)

^1^H NMR (600 MHz, DMSO-*d*_6_) δ: 7.65 (d, *J* = 7.6 Hz, 2H), 7.56 – 7.34 (m, 3H), 7.30 (t, *J* = 7.3 Hz, 1H), 7.23 – 6.92 (m, 2H), 6.12 (d, *J* = 15.6 Hz, 1H); ^13^C NMR (151 MHz, DMSO-*d*_6_) δ: 169.25, 142.17, 141.51, 133.58, 130.71, 129.16, 129.04, 128.73, 127.95, 127.79, 127.76, 125.43, 125.27, 125.14, 124.33, 39.94, 39.80, 39.66, 39.52, 39.38, 39.24, 39.10.

*(E)-3-(5-(4-bromophenyl)furan-2-yl)acrylic acid* (**1v**)

^1^H NMR: (300 MHz, DMSO-*d*_6_) δ: 6.34 (d, *J* = 15.5 Hz, 1H); 6.40 (d, *J* = 3.8 Hz, 1H); 6.93 (d, *J* = 3.8 Hz, 1H); 7.39 (d, *J* = 15.5 Hz, 1H); 7.60 (d, *J* = 8.6 Hz, 2H); 7.80 (d, *J* = 8.6 Hz, 2H); ^13^C NMR: (75 MHz, DMSO-*d*_6_) δ: 106.3, 109.8, 120.0, 123.7, 126.1, 130.4, 131.7, 132.1, 149.0, 150.8, 170.3.

*(E)-3-(2-phenylthiazol-4-yl)acrylic acid* (**1w**)

^1^H NMR (400 MHz, DMSO-*d*_6_) δ: 8.55 (s, 1H), 8.41 (dd, *J* = 6.5, 2.9 Hz, 2H), 8.04 (d, *J* = 15.5 Hz, 1H), 8.00 – 7.91 (m, 3H), 7.10 (d, *J* = 15.5 Hz, 1H); ^13^C NMR (101 MHz, DMSO-*d*_6_) δ: 168.31, 168.01, 152.36, 136.70, 133.00, 131.22, 129.78, 126.89, 124.05, 121.42.

*(E)-3-(10-methyl-10H-phenothiazin-3-yl)acrylic acid* (**1x**)

^1^H NMR (400 MHz, DMSO-*d*_6_) δ: 7.73 – 6.65 (m, 8H), 6.43 (s, 1H), 2.50 (s, 3H); ^13^C NMR (101 MHz, DMSO-*d*_6_) δ: 167.79, 146.82, 144.47, 142.92, 128.70, 128.48, 127.93, 126.88, 126.18, 122.95, 122.49, 121.49, 117.07, 114.94, 114.66, 40.15, 39.94, 39.73, 39.52, 39.31, 39.10, 38.89, 35.36.

# 4. The effect of pH upon the conversion values of biotransformation of 1i

**
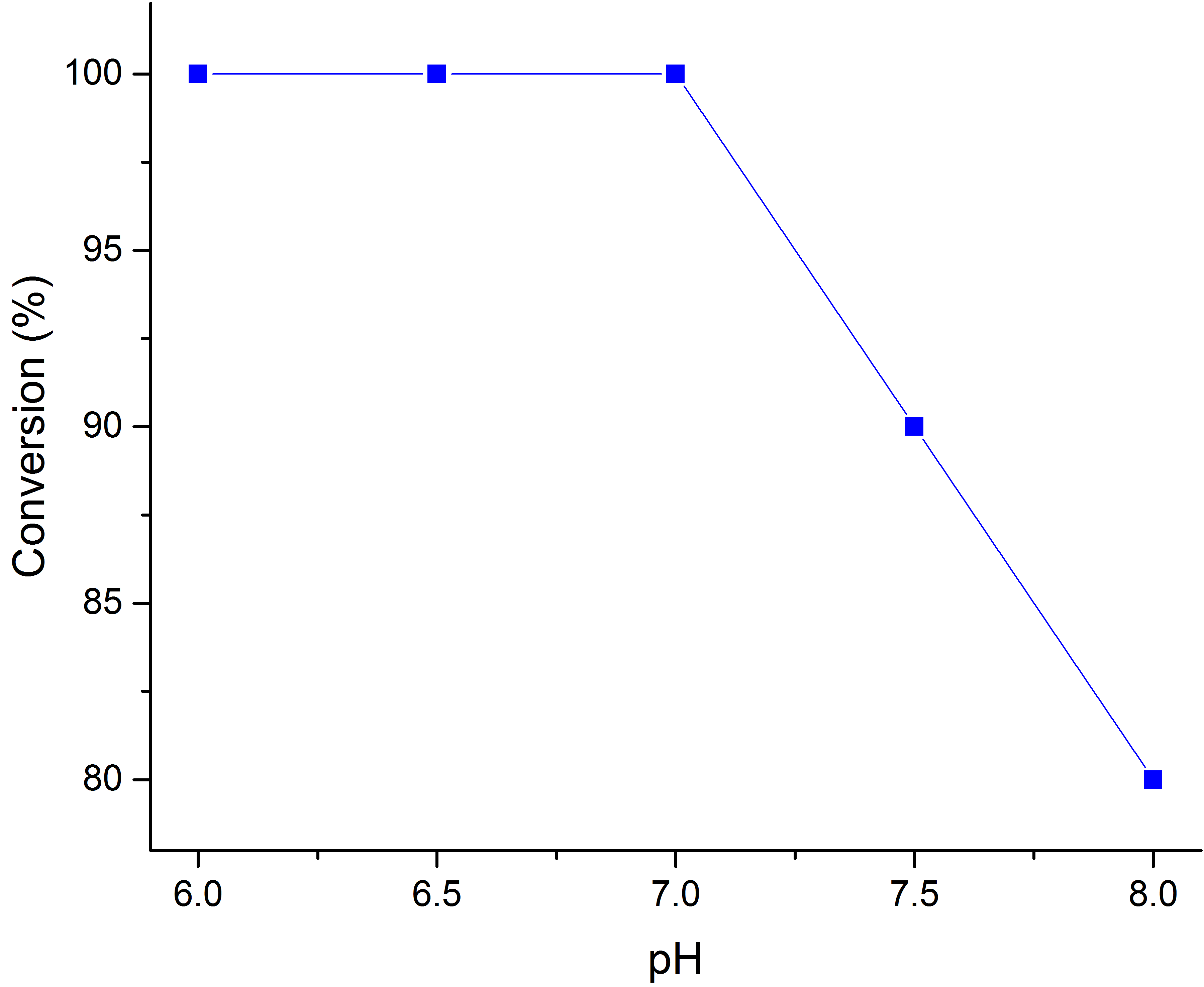
**

**Figure S70.** Conversion-pH profile for the FDC1 catalyzed decarboxylation of **1i**

# 5. Molecular docking

| **(a)** | **4ZA7**   \| 173 \| 185 \| 187 \| 190 \| 280 \| 281 \| 282 \| 283 \| 284 \| 316 \| 323 \| 327 \| 394 \| 395 \| 437 \| 439 \| 439 \| \| --- \| --- \| --- \| --- \| --- \| --- \| --- \| --- \| --- \| --- \| --- \| --- \| --- \| --- \| --- \| --- \| --- \| \| R \| L \| I \| Q \| F \| G \| E \| M \| H \| C \| T \| I \| Y \| T \| F \| L \| L \| |
| --- | --- | --- | --- | --- | --- | --- | --- | --- | --- | --- | --- | --- | --- | --- | --- | --- | --- | --- | --- | --- | --- | --- | --- | --- | --- | --- | --- | --- | --- | --- | --- | --- | --- | --- | --- |
|  | **4ZAC**   \| 175 \| 187 \| 189 \| 192 \| 283 \| 284 \| 285 \| 286 \| 287 \| 319 \| 326 \| 330 \| 397 \| 398 \| 440 \| 442 \| 442 \| \| --- \| --- \| --- \| --- \| --- \| --- \| --- \| --- \| --- \| --- \| --- \| --- \| --- \| --- \| --- \| --- \| --- \| \| R \| L \| I \| Q \| F \| G \| E \| M \| H \| P \| T \| I \| F \| I \| F \| L \| L \| |
|  |  |
| **(b)** | 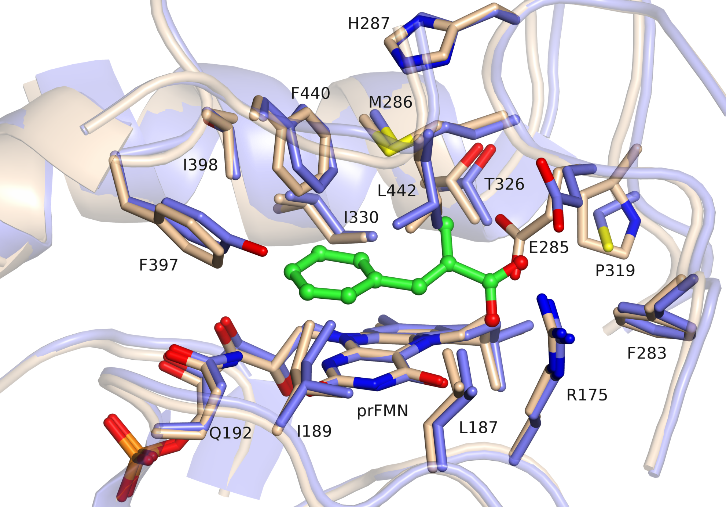 |

**Figure S71. a)** Sequence alignment of the active sites overlay from *An*FDC1 (PDB code: 4ZA7) and *Sc*FDC1 (PDB code: 4ZAC) . Active site residues were selected at 6 Å around the α-methyl-cinnamate ligand found in the structure of *An*FDC1. **b)** Superimposed active sites of FDC1 from *Aspergillus* *niger* binding α-methyl-cinnamate as ligand (blue-coloured, PDB code: 4ZA7) and *Sc*FDC1 (tan-coloured, PDB code: 4ZAC)

**
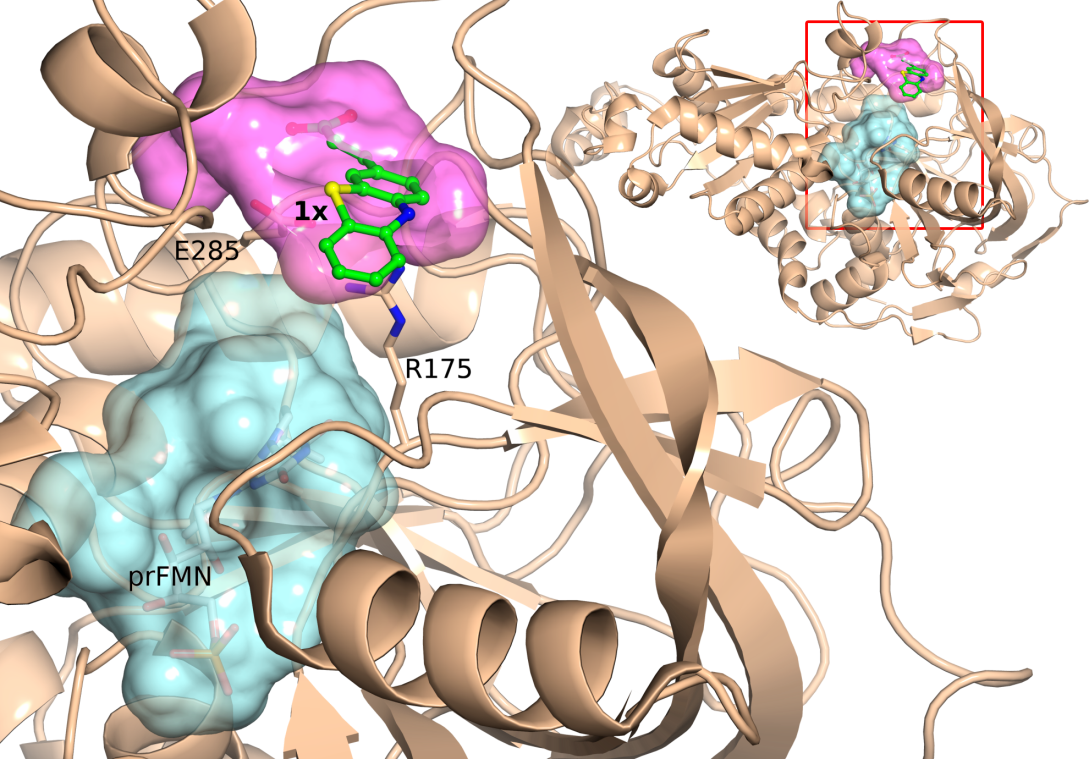
**

**Figure S72.** Cartoon representation of the overall FDC1 enzyme, the position of binding pocket is highlighted with blue surface representation. The zoom view shows the energetically favoured position of **1x** in the surface cavity (pink coloured) located above the catalytic site of FDC1.

| 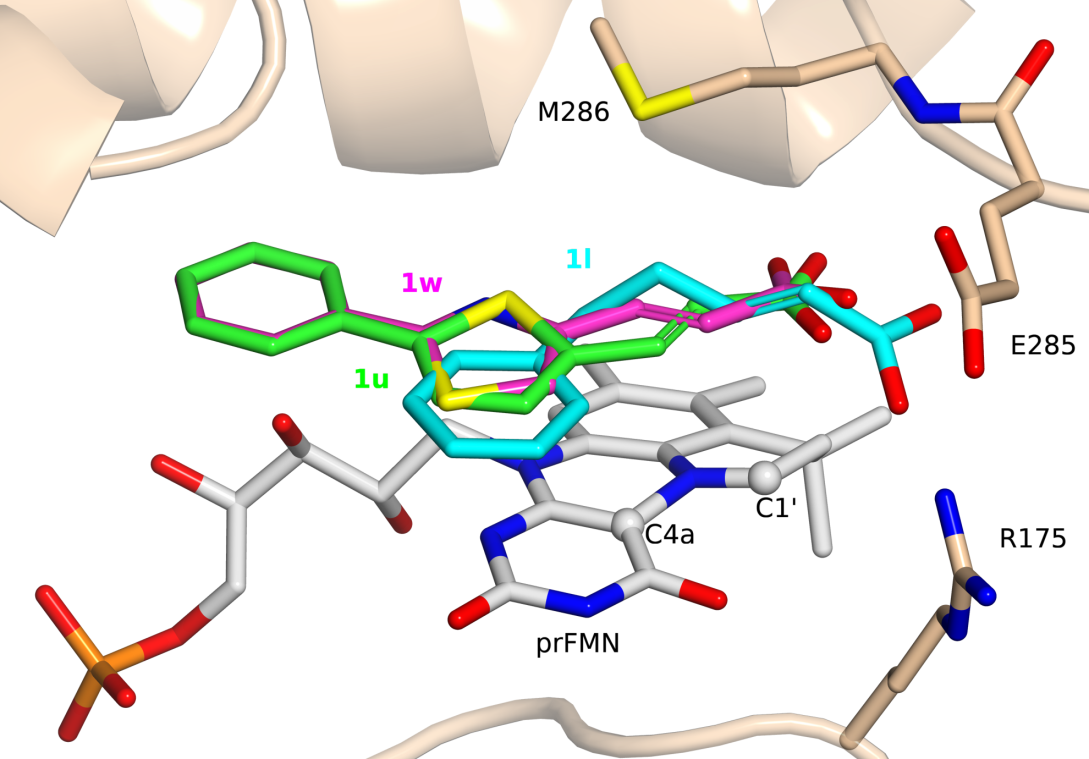 |
| --- |
| **Figure S73**. The inactive pose of **1w** (magenta) and **1l** (blue) within the catalytic site of FDC1, with the double bond located in an arrangement with respect to prFMN, which is unfavourable for the 1,3-cycloaddition in comparison with the productive orientation of bulky **1u** (green) |

| 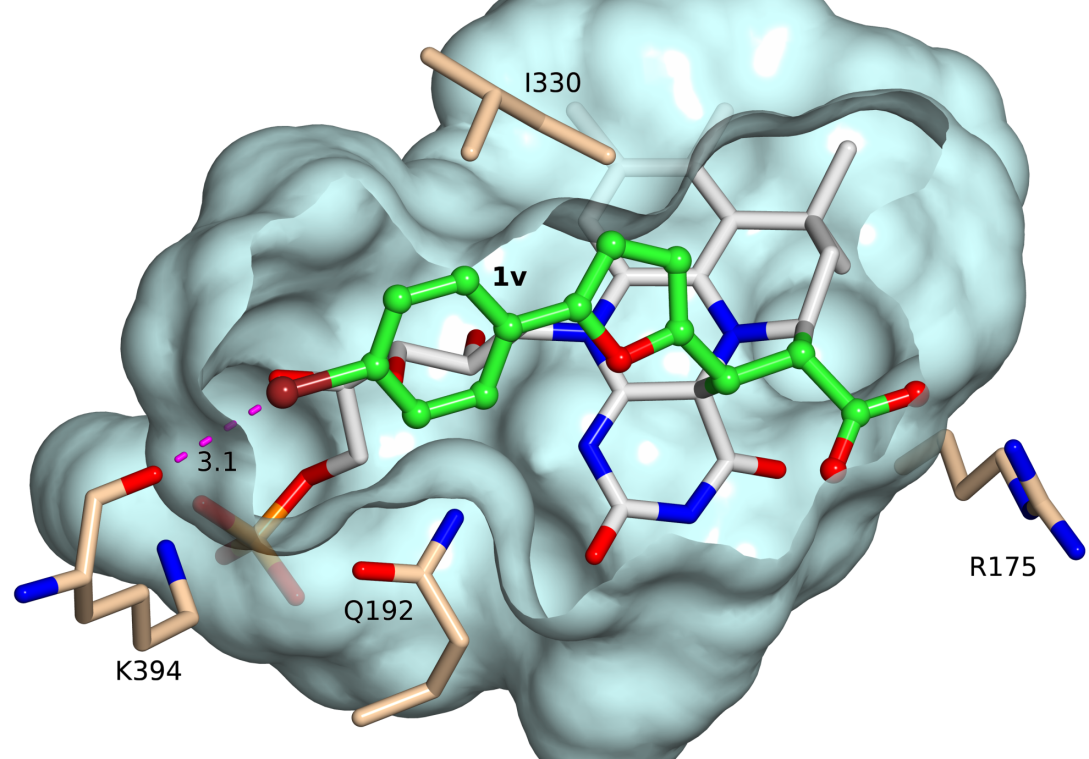 |
| --- |
| **Figure S74.** Ground state conformation of substrate **1v** in the cycloadduct is hindered by the proximity of the bromine atom and the backbone carbonyl of residue K394. |

| 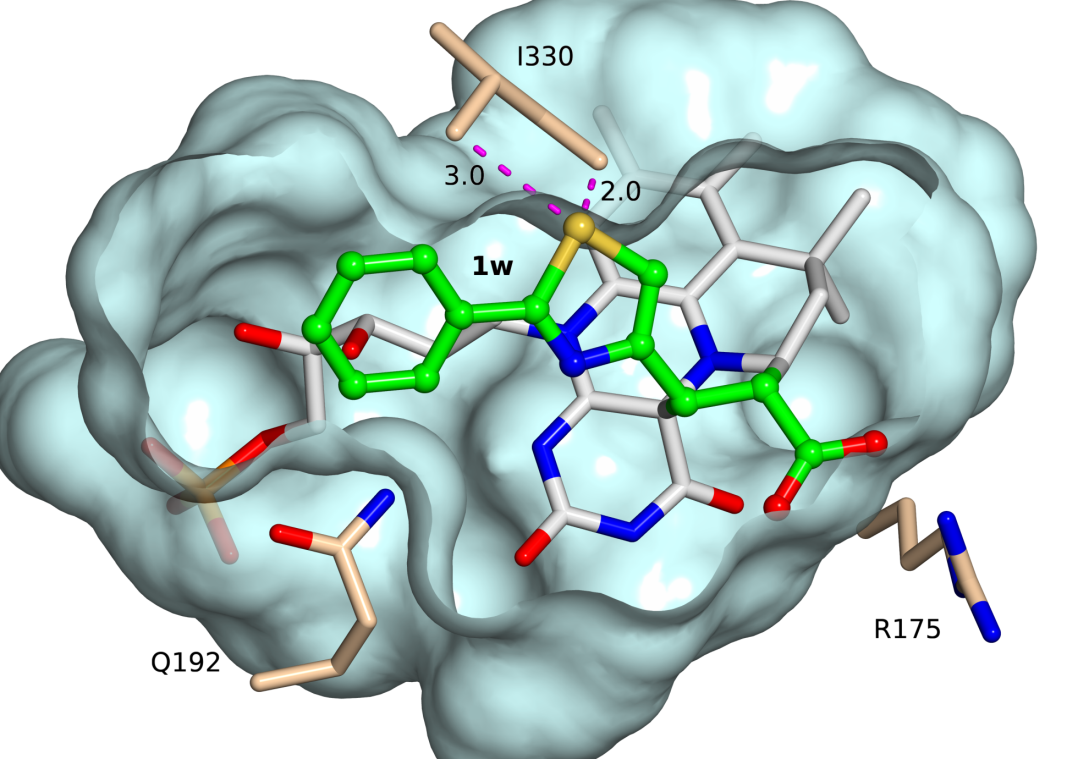 |
| --- |
| **Figure S75.** Steric clashes between the sulphur atom of covalent intermediate, formed by **1w** and prFMN, and residue I330 are highlighted by violet dotted lines. |

**
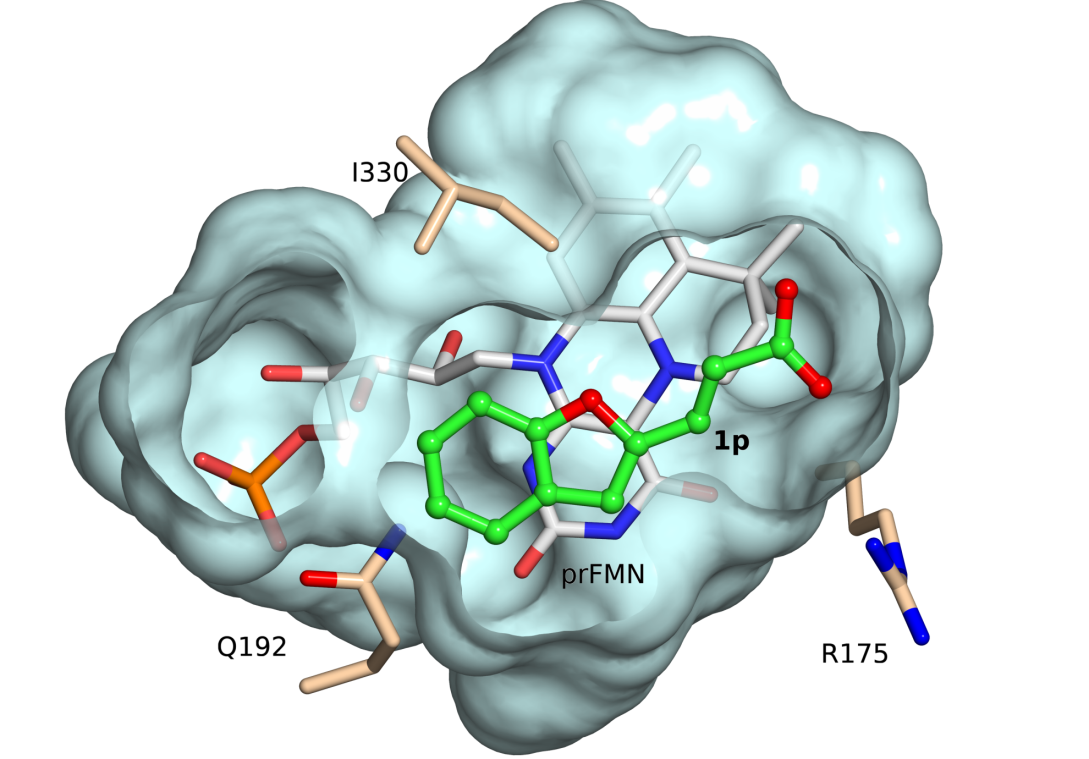
**

| **Figure S76.** Narrowing of the catalytic site (blue surface) by residues Q192 and I330 doesn't influence the proper binding of substrates containing condensed aromatic systems (e.g. **1p**). |
| --- |

| 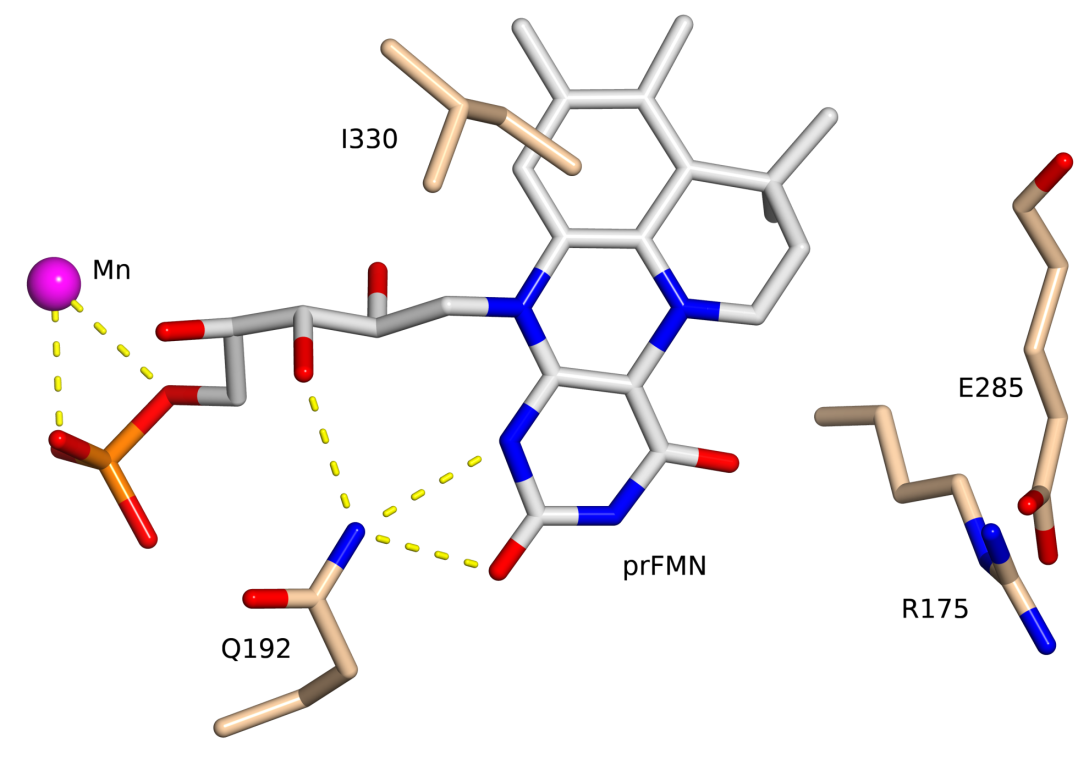 |
| --- |
| **Figure S77.** The role of residue Q192 in binding the ribitol tail of the prFMN cofactor |

**6. Expression levels of *Sc*FDC1- SDS-PAGE**

**
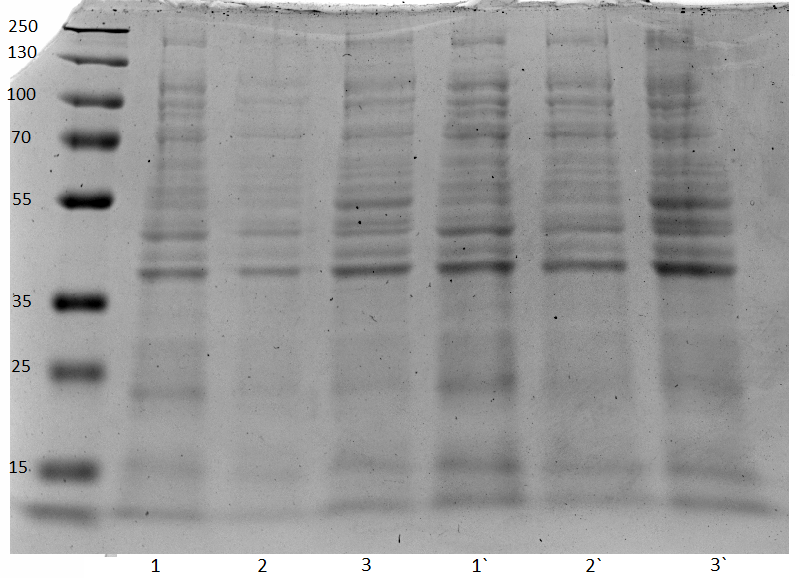
**

**Figure S78.** SDS-PAGE analysis of whole cell samples obtained from the expression of E.coli harbouring the recombinant plasmid of *Sc*FDC1. 1 – cells before induction at OD_600_ of 0.7; 2- cells grown additional 4.5 h without induction; 1`,2`,3`- samples 1,2,3 loaded in higher concentration. The band appearing in samples 3 and 3’ at 55 kDa corresponds to *Sc*FDC1 (56.1 kDa)

# 7. Site-directed mutagenesis

**Table S4.** List of the primers used for mutagenesis and sequencing

| **Entry** | **Primer** | | **sequence 5'-3'** | | **Tm _pp_** | **Tm _no_** |
| --- | --- | --- | --- | --- | --- | --- |
| 1 | FDC1/Q192N/FP | | 5'ttaaaccaaatcatattagacaaattgctgactcttggg3’ | | 53 | 60.8 |
| 2 | | FDC1/Q192N/RP | | 5’gtctaatatgatttggtttaattaccagaccagtgatatgc3’ | 53 | 60.5 |
| 3 | | FDC1/Q192A/FP | | 5’ttaaaccagcacatattagacaaattgctgactcttgg3’ | 53 | 60 |
| 4 | | FDC1/Q192A/RP | | 5’ctaatatgtgctggtttaattaccagaccagtgatatgct3’ | 53 | 61 |
| 5 | | FDC1/I330A/FP | | 5’ccttggctggttcactagtggctactgaggcc3’ | 53.8 | 61.4 |
| 6 | | FDC1/I330A/RP | | 5’gaaccagccaaggtatgtgtctcatccgtacaaag3’ | 53.8 | 61.9 |
| 7 | | FDC1/I330V/FP | | 5’ccttggttggttcactagtggctactgaggccaag3’ | 55.7 | 64.7 |
| 8 | | FDC1/I330V/RP | | 5’gtgaaccaaccaaggtatgtgtctcatccgtacaaagac3’ | 55.7 | 64.3 |
| 9 | | FDC1_seqM | | 5’ctgcaccttgtaaaacac3’ |  |  |
| 10 | | Duet_DOWN1 | | 5’gattatgcggccgtgtacaa3’ |  |  |
| 11 | | T7promoter | | custom primer |  |  |
